# Impact of CRM197-based conjugate vaccines, schedules, and regions on pneumococcal immunogenicity in young children: systematic review

**DOI:** 10.1038/s41541-026-01395-y

**Published:** 2026-03-06

**Authors:** Xinghui Chen, Sarah Tavlian, Kylie S. Carville, Nefel Tellioglu, Violeta Spirkoska, Natalie Carvalho, David J. Price, Patricia T. Campbell, Jodie McVernon

**Affiliations:** 1 Department of Infectious Diseases, The University of Melbourne, at the Peter Doherty Institute for Infection and Immunity, Melbourne, Australia; 2 Victorian Infectious Diseases Reference Laboratory, The Royal Melbourne Hospital, at the Peter Doherty Institute for Infection and Immunity, Melbourne, Australia; 3 Centre for Health Policy, Melbourne School of Population and Global Health, The University of Melbourne, Melbourne, Australia; 4 Centre for Epidemiology and Biostatistics, Melbourne School of Population and Global Health, The University of Melbourne, Melbourne, Australia

**Keywords:** Diseases, Immunology, Medical research, Microbiology

## Abstract

Population-level introduction of pneumococcal conjugate vaccines (PCVs) has increased non-vaccine serotype invasive pneumococcal disease (IPD) incidence in children. Higher-valency PCVs were developed to address shifting disease-causing serotypes. This systematic review and meta-analysis defines trends in CRM197-based PCV immunogenicity in children < 2 years. We searched five databases—EMBASE, MEDLINE, Web of Science Core Collection, Global Health, and Cochrane Central Register of Controlled Trials. Random-effects meta-analyses were conducted using log-transformed IgG GMCs and logit-transformed seroresponse rates to generate pooled estimates. We included 250 articles from 138 study groups involving 243 study arms. Vaccine immunogenicity varied by serotype, vaccine, schedule and region. Pooled IgG GMCs post-childhood-schedule were lowest for serotype 3-PCV20 (0.84 μg/mL; 95%CI: 0.60–1.17), and highest for 15B-PCV20 (16.00 μg/mL; 95%CI: 12.31–20.80). Post-childhood-schedule seroresponse rates were >95% for all serotypes except 3. IgG responses increased with primary-dose number, and were further enhanced by a booster, although magnitude varied by serotype and vaccine; for PCV20, IgG GMCs after two-primary doses were still below the 0.35 µg/mL threshold for six serotypes. A general downward trend in IgG GMCs was observed with increasing vaccine valency. Regional variation in post-childhood-schedule IgG GMCs was observed, with highest GMCs in the Western Pacific Region.

## Introduction

*Streptococcus pneumoniae* (the pneumococcus) is a multi-strain bacterial pathogen, with more than 100 immunologically distinct serotypes^1^, that causes a substantial global disease burden in children under 5 years^2^. Pneumococcal conjugate vaccines (PCVs) cover only a small proportion of disease-causing serotypes. Following widespread introduction of PCVs in several populations, immunological selection resulted in shifts in the pneumococcal serotype distribution^3^. Emergence of “non-vaccine” serotypes has been associated with replacement disease in many settings, a phenomenon termed “serotype replacement”, lessening vaccine impact^4^.

In response to changes in the distribution of disease-causing serotypes, higher-valency PCVs have been developed to target a broader spectrum of serotypes emerging as a cause of disease. Despite the transition from 7-valent PCV (PCV7) to 10-valent PCV (PCV10-GSK) and 13-valent PCV (PCV13), serotype replacement continues to occur^5^. Recently, newer PCVs with even higher valency such as 15-valent PCV (PCV15), 20-valent PCV (PCV20) and 21-valent PCV (V116, for use in adults only) have been licensed. In 2019, a novel 10-valent PCV (PCV10-SII), designed to protect against serotypes causing most diseases in low- and middle-income countries, received prequalification status by the World Health Organization (WHO).

However, these newer vaccines, starting with PCV10-GSK, are evaluated indirectly, using immunogenicity studies to infer vaccine efficacy. This process, known as immunobridging, compares immune responses from new candidate vaccines with those of licensed comparators whose efficacy has already been established^6,7^. WHO has defined a post-primary immunisation immunoglobulin G (IgG) geometric mean concentration (GMC) of 0.35 μg/mL as a putative correlate of protection against invasive pneumococcal disease (IPD)^8^. While this is an average measure that may differ for individual serotypes^9^, it remains a widely accepted threshold for regulatory approval. However, it is important to recognise that substantially higher levels of IgG are likely required to protect against colonization and other mucosal infections such as acute otitis media^10,11^.

Regulatory agencies such as the U.S. Food and Drug Administration^12^ and European Medicines Agency^13^ accept immunobridging for licensure in infants in lieu of efficacy studies if immune responses for shared serotypes meet non-inferiority criteria compared to licensed vaccines. These assessments are typically based on both IgG GMCs and seroresponse rates (i.e., proportion of participants meeting a serotype-specific protective threshold against IPD, usually 0.35 μg/mL)^14^. The downward trend in immune responses associated with expanded serotype coverage, and the successive use of non-inferiority comparisons, means that this “bridge to a bridge” process has sequentially lowered the bar needed to meet non-inferiority^15^. There is potential for real-world vaccine effectiveness to be compromised if antibody levels induced by approved vaccines do not maintain a protective threshold.

Previous studies have compared the immunogenicity of PCV7, PCV10-GSK, and PCV13^16–19^; however, none has comprehensively compared these vaccines alongside the more recently licensed higher-valency formulations and PCV10-SII, in combination with different dosing schedules and regional settings, which is important to inform effective pneumococcal vaccination strategies. In this study, we conducted a systematic review and meta-analysis of post-vaccination immune responses in infants by serotype, vaccine product, dosing schedule and geographic region to define trends in CRM197-based PCV immunogenicity in children <2 years.

## Results

**Fig. 1:**
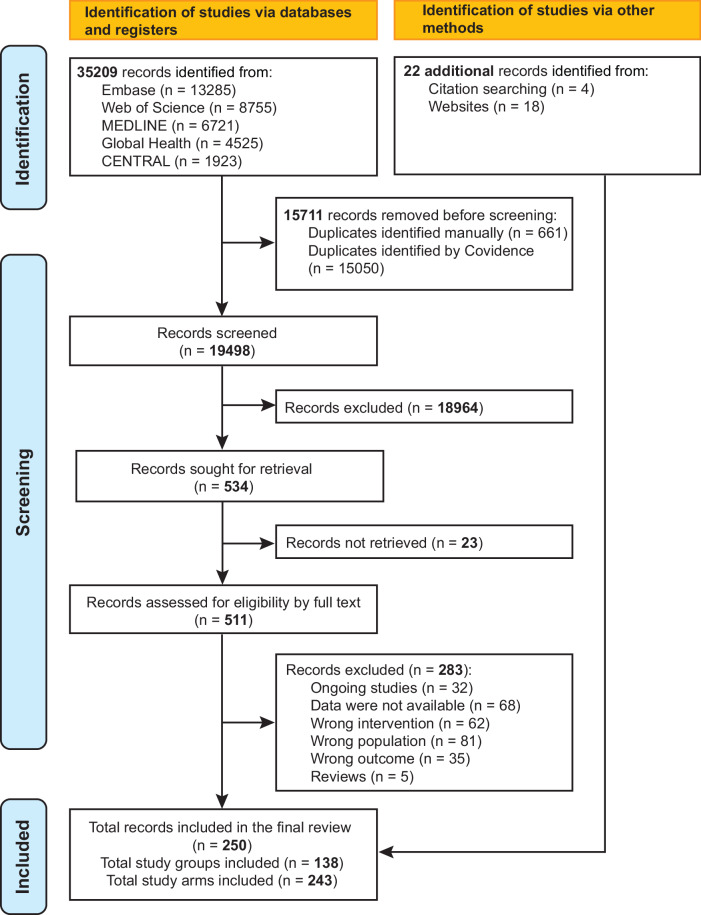
PRISMA flow diagram to show study selection process. The figure illustrates the process of identification, screening, and inclusion of study groups and study arms evaluating the immunogenicity of pneumococcal vaccination in children under 2 years of age, in accordance with the PRISMA 2020 guidelines.

After screening and full-text review, a total of 138 study groups from 250 articles were included (Fig. 1). The included study groups comprised 110 randomized controlled trials (RCTs), 14 quasi-experimental studies, and 14 prospective cohort studies. Risk of bias was low in 110 study groups, moderate in 12, and high in 16. The characteristics of the individual study groups and the modified risk of bias assessments are reported in the Supplementary Tables 12, 14–16.

These 138 study groups included 243 study arms. Their main characteristics and spatiotemporal distributions are summarized in Table 1, Supplementary Tables 17, 18 and Supplementary Figs. 1, 2. Most study arms investigated the immunogenicity after PCV13 (*n* = 122) and PCV7 (*n* = 101) vaccination, while fewer evaluated PCV15 (*n* = 12), PCV20 (*n* = 5) and PCV10-SII (*n* = 3). The most common vaccine schedules were the 3 + 1 (*n* = 167) and 2 + 1 schedule (*n* = 49), while 3 + 0 (*n* = 19), 1 + 1 (*n* = 7) and 0 + 1 (*n* = 1) schedules were only investigated in study arms using PCV7 and/or PCV13. Most study arms used either a 2, 4 and 6 (*n* = 103) or 2, 3, 4 months (*n* = 48) primary series, with booster most commonly administered at 12–15 months (*n* = 181). Despite the highest disease burden in African and Asian countries, most study arms were conducted in the European (*n* = 77) and Americas (*n* = 61) regions, particularly the United States (*n* = 42), with relatively few in the African (*n* = 20) and South-East Asia (*n* = 15) regions. An exception was PCV10-SII, all study arms were conducted in Gambia, African region (*n* = 3).

**Table 1:** Characteristics of all included study arms by vaccine product

Characteristics	Total (*n* = 243)	PCV7 (*n* = 101)	PCV13 (*n* = 122)	PCV15 (*n* = 12)	PCV20 (*n* = 5)	PCV10-SII (*n* = 3)
Study period (year)						
1995–1999	8/243 (3.3%)	8/101 (7.9%)	-	-	-	-
2000–2009	107/243 (44.0%)	83/101 (82.2%)	24/122 (19.7%)	-	-	-
2010–2019	105/243 (43.2%)	10/101 (9.9%)	84/122 (68.9%)	7/12 (58.3%)	1/5 (20.0%)	3/3 (100%)
2020–2025	23/243 (9.5%)	-	14/122 (11.5%)	5/12 (41.7%)	4/5 (80.0%)	-
WHO region						
Africa	20/243 (8.2%)	4/101 (4.0%)	13/122 (10.7%)	-	-	3/3 (100%)
Americas	61/243 (25.1%)	36/101 (35.6%)	22/122 (18.0%)	1/12 (8.3%)	2/5 (40.0%)	-
Europe	77/243 (31.7%)	38/101 (37.6%)	38/122 (31.1%)	1/12 (8.3%)	-	-
South-East Asia	15/243 (6.2%)	4/101 (4.0%)	11/122 (9.0%)	-	-	-
Western Pacific	41/243 (16.9%)	15/101 (14.9%)	20/122 (16.4%)	4/12 (33.3%)	2/5 (40.0%)	-
Multiple regions	29/243 (11.9%)	4/101 (4.0%)	18/122 (14.8%)	6/12 (50.0%)	1/5 (20.0%)	-
Income group						
Low income	11/243 (4.5%)	-	8/122 (6.6%)	-	-	3/3 (100%)
Lower middle income	18/243 (7.4%)	6/101 (5.9%)	12/122 (9.8%)	-	-	-
Upper middle income	32/243 (13.2%)	15/101 (14.9%)	17/122 (13.9%)	-	-	-
Upper middle income, High income	16/243 (6.6%)	5/101 (5.0%)	8/122 (6.6%)	3/12 (25.0%)	-	-
High income	166/243 (68.3%)	75/101 (74.3%)	77/122 (63.1%)	9/12 (75.0%)	5/5 (100%)	-
Population type						
General population	240/243 (98.8%)	100/101 (99.0%)	120/122 (98.4%)	12/12 (100%)	5/5 (100%)	3/3 (100%)
Indigenous peoples	3/243 (1.2%)	1/101 (1.0%)	2/122 (1.6%)	-	-	-
Vaccine schedule						
3 + 1	167/243 (68.7%)	82/101 (81.2%)	70/122 (57.4%)	9/12 (75.0%)	4/5 (80.0%)	2/3 (66.7%)
2 + 1	49/243 (20.2%)	11/101 (10.9%)	33/122 (27.0%)	3/12 (25.0%)	1/5 (20.0%)	1/3 (33.3%)
3 + 0	19/243 (7.8%)	8/101 (7.9%)	11/122 (9.0%)	-	-	-
1 + 1	7/243 (2.9%)	-	7/122 (5.7%)	-	-	-
0 + 1	1/243 (0.4%)	-	1/122 (0.8%)	-	-	-
Administration of vaccine						
Intramuscular injection	233/243 (95.9%)	99/101 (98.0%)	117/122 (95.9%)	10/12 (83.3%)	4/5 (80.0%)	3/3 (100%)
Subcutaneous injection	10/243 (4.1%)	2/101 (2.0%)	5/122 (4.1%)	2/12 (16.7%)	1/5 (20.0%)	-
Age of first dose (Month)						
<2	25/243 (10.3%)	7/101 (6.9%)	16/122 (13.1%)	-	-	2/3 (66.7%)
2-4	212/243 (87.2%)	92/101 (91.1%)	103/122 (84.4%)	11/12 (91.7%)	5/5 (100%)	1/3 (33.3%)
>4	5/243 (2.1%)	2/101 (2.0%)	2/122 (1.6%)	1/12 (8.3%)	-	-
No primary series	1/243 (0.4%)	-	1/122 (0.8%)	-	-	-
Interval between primary doses (Month)						
1	80/243 (32.9%)	34/101 (33.7%)	38/122 (31.1%)	4/12 (33.3%)	2/5 (40.0%)	2/3 (66.7%)
2^*^	155/243 (63.8%)	67/101 (66.3%)	76/122 (62.3%)	8/12 (66.7%)	3/5 (60.0%)	1/3 (33.3%)
No interval	8/243 (3.3%)	-	8/122 (6.6%)	-	-	-
Age at booster (Month)						
9–11	32/243 (13.2%)	10/101 (9.9%)	20/122 (16.4%)	-	-	2/3 (66.7%)
12–15	181/243 (74.5%)	76/101 (75.2%)	87/122 (71.3%)	12/12 (100%)	5/5 (100%)	1/3 (33.3%)
16–18	11/243 (4.5%)	7/101 (6.9%)	4/122 (3.3%)	-	-	-
No booster	19/243 (7.8%)	8/101 (7.9%)	11/122 (9.0%)	-	-	-
Laboratory method						
First-generation ELISA	2/243 (0.8%)	2/101 (2.0%)	-	-	-	-
Second-generation ELISA	14/243 (5.8%)	13/101 (12.9%)	1/122 (0.8%)	-	-	-
Third-generation ELISA	131/243 (53.9%)	57/101 (56.4%)	71/122 (58.2%)	-	-	3/3 (100%)
GSK 22F-ELISA	19/243 (7.8%)	11/101 (10.9%)	8/122 (6.6%)	-	-	-
Pfizer dLIA platform	14/243 (5.8%)	-	9/122 (7.4%)	-	5/5 (100%)	-
Pn ECL assay	32/243 (13.2%)	1/101 (1.0%)	19/122 (15.6%)	12/12 (100%)	-	-
FMIA	9/243 (3.7%)	-	9/122 (7.4%)	-	-	-
ELISA without detail	22/243 (9.1%)	17/101 (16.8%)	5/122 (4.1%)	-	-	-

^*^Interval between primary doses was 1.5 months for one study arm and was grouped into 2 months group.

**Fig. 2:**
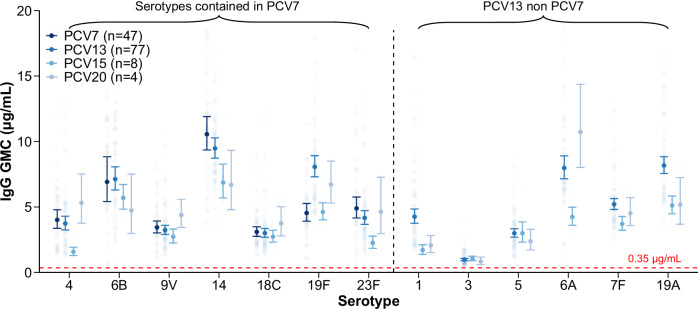
Pneumococcal IgG GMCs (μg/mL) post-childhood-schedule by serotype and vaccine product. Serotypes shared by most vaccine products are shown. The full panel of all included serotypes, as well as those unique to PCV15/20, is provided in Supplementary Table 12 and Supplementary Fig. 3. Numbers in parentheses following each vaccine name in the legend indicate the number of study arms included in the meta-analysis contributing to the pooled estimates. The horizontal line represents the 0.35 μg/mL WHO-defined putative protective threshold against IPD, which was developed for post-infant primary series responses and is not a defined correlate for booster doses or other pneumococcal endpoints; it is shown here as a reference line only, noting that serotype-specific protective thresholds may vary.

There were 139 study arms that reported IgG GMCs and 88 that reported IgG seroresponse rates post-childhood-schedule (Supplementary Table 19). Overall, pooled IgG GMCs for all vaccine-included serotypes (VTs) post-childhood-schedule were all above the WHO-defined putative protective threshold against IPD of 0.35 μg/mL (referred to hereafter as the protective threshold) (Fig. 2, Supplementary Table 23 and Supplementary Fig. 3). Pooled seroresponse rates were >95% for all VTs except serotype 3 (ranges of point estimates: 84–92%) (Supplementary Table 24 and Supplementary Fig. 4). However, pooled IgG GMCs varied considerably across serotypes, with serotypes 14 and 6B consistently high and serotype 3 consistently low across different vaccine products, with the lowest IgG GMC observed for serotype 3 (PCV20) at 0.84 μg/mL (95% confidence interval [CI]: 0.60–1.17). A general “downward trend” in IgG GMCs was observed with increasing vaccine valency. For PCV7-included serotypes, the IgG GMCs post PCV7 were generally higher than those post PCV13 and PCV15, except for serotype 6B and 19F. A similar trend of decreasing IgG GMCs with increasing valency was found for serotypes shared between PCV13 and higher-valency vaccines. In contrast, PCV20 had higher point estimates for serotypes 4, 9V, 18C, and 6A than other PCVs, noting that CIs were wide. After adjusting for assay differences, the expected trend of decreasing IgG levels with increasing valency became apparent: PCV20 showed lower point estimates than PCV13 for all shared serotypes, again with overlapping CIs (Supplementary Fig. 5). PCV10-SII initially appeared to be an exception, showing a distinct IgG response profile: serotypes 6B, 1, and 7F induced substantially higher IgG responses than other PCVs, whereas 9V and 5 were noticeably lower (Supplementary Table 23 and Supplementary Fig. 3). However, post-hoc analyses restricted to studies conducted in the African Region—where only PCV13 and PCV10-SII data were available—showed that its serotype-specific IgG responses were similar across shared serotypes between PCV10-SII and PCV13 (Supplementary Fig. 6).

**Fig. 3:**
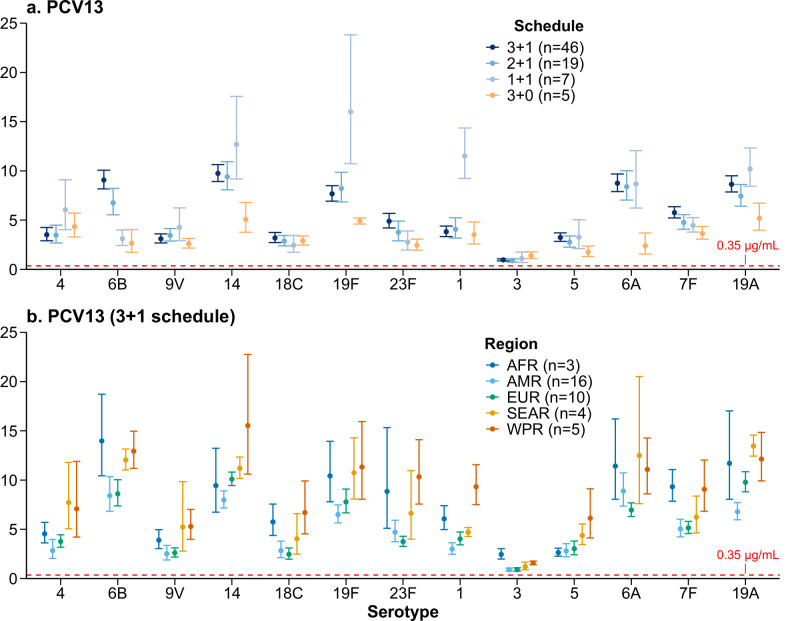
Exemplar serotype-specific pneumococcal IgG GMCs (μg/mL) post-childhood-schedule, by schedule and region. Panel (**a**) PCV13 stratified by schedule; (**b**) PCV13 stratified by region (3 + 1 schedule). Full panels are provided in the Supplementary Table 14–17 and Supplementary Figs. 7–10. Numbers in parentheses following each vaccine name in the legend indicate the number of study arms included in the meta-analysis contributing to the pooled estimates. The horizontal line in each panel represents the 0.35 μg/mL WHO-defined putative protective threshold against IPD, which was developed for post-infant primary series responses and is not a defined correlate for booster doses or other pneumococcal endpoints; it is shown here as a reference line only, noting that serotype-specific protective thresholds may vary. AFR African Region, AMR Region of the Americas, EUR European Region, SEAR South-East Asia Region, WPR Western Pacific Region.

Serotype-specific IgG GMCs and seroresponse rates measured post-childhood-schedule varied by vaccine schedule (see Supplementary Table 20 for study arm numbers by schedule). Overall, vaccine schedules that included a booster dose (3 + 1, 2 + 1 or 1 + 1) generally elicited higher post-childhood-schedule IgG responses (both IgG GMCs and seroresponse rates; hereafter IgG responses) compared to a primary-only (3 + 0) schedule (Fig. 3a, Supplementary Tables 25, 26 and Supplementary Figs. 7, 8). The 3 + 1 schedule generally induced immune responses that were higher than or comparable to those induced by the 2 + 1 schedule across all serotypes for every vaccine except PCV10-SII, for which the 2 + 1 schedule induced higher IgG response. The 1 + 1 schedule—although less commonly used and only tested in PCV13—produced even higher point estimates of IgG responses than 3 + 1/2 + 1 schedules for specific serotypes (e.g., serotypes 1, 14, 19F) following the booster dose.

Given the impact of vaccine schedules on immune responses, regional analyses were restricted to study arms administering a 3 + 1 schedule—the most extensively studied regimen—to reduce heterogeneity and improve comparability (see Supplementary Table 21 for study arm numbers by WHO region). We observed substantial regional variation of serotype-specific IgG GMCs post-childhood-schedule (Fig. 3b, Supplementary Tables 27, 28 and Supplementary Figs. 9, 10). IgG GMCs were generally highest in the Western Pacific Region (WPR), followed by the African Region (AFR) and South-East Asia Region (SEAR), and lowest in the Region of the Americas (AMR), and the European Region (EUR), regardless of vaccine product. Post-hoc analyses of PCV13 across all vaccine schedules showed that IgG responses in WPR using 2 + 1 schedule were higher than or comparable with those in the AMR or EUR using 3 + 1 for most VTs (Supplementary Fig. 11). For example, the IgG GMC for serotype 23F with a 2 + 1 schedule in the WPR was 7.73 (95% CI: 4.87–12.28), compared with 4.70 (95% CI: 3.74–5.91) for the 3 + 1 schedule in the AMR. In contrast, seroresponse rates were >95% for most serotypes, except serotype 3, with minimal regional differences (Supplementary Table 28 and Supplementary Figs. 10, 12).

**Fig. 4:**
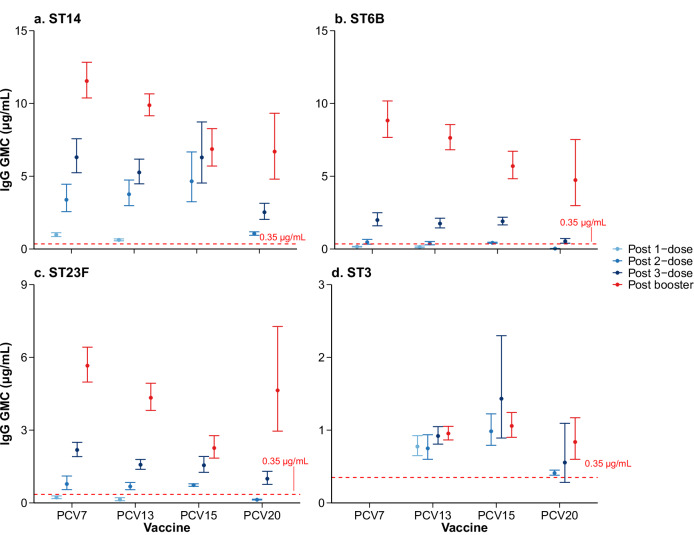
Exemplar serotype-specific pneumococcal IgG GMCs (μg/mL) by vaccine product and time point of blood sample collection. Panels depict the following serotypes: (**a**) serotype 14; (**b**) serotype 6B; (**c**) serotype 23F; and (**d**) serotype 3. The full panel is provided in the Supplementary Table 18 and Supplementary Fig. 13. The horizontal line in each panel represents the 0.35 μg/mL WHO-defined putative protective threshold against IPD, which was developed for post-infant primary series responses and is not a defined correlate for booster doses or other pneumococcal endpoints; it is shown here as a reference line only, noting that serotype-specific protective thresholds may vary.

The overall immune response post-childhood-schedule provides only part of the picture. We further assessed both serotype-specific IgG GMCs and seroresponse rates at post 1-, 2-, 3-primary doses, and post-booster (see Supplementary Table 22 for study arm numbers by timepoint). Overall, IgG responses generally increased with the number of primary doses, and were further enhanced by a booster, although magnitude of this increase varied by serotype and vaccine; downward trends in IgG GMC with increasing valency persisted across timepoints (Fig. 4, Supplementary Table 29–30 and Supplementary Figs. 13–15). Post 1-dose data (PCV7/PCV13 only) showed low IgG GMCs and seroresponse rates for most serotypes (<80%). For example, the post 1-primary dose IgG GMCs for 6B, 6A, 9V and 23F were low and in some cases overlapped with the protective threshold (Supplementary Figs. 13, 14). After 2-primary doses, PCV10-SII IgG GMCs exceeded the protective threshold for VTs, as did PCV7, PCV13, and PCV15 for most serotypes (except serotypes 6B/23F and PCV15-specific 6A/33F), while PCV20 remained low (six serotypes below protective threshold and 13 serotypes with seroresponse rates <80%). Post 3-dose primary vaccination, all PCVs achieved GMCs above protective threshold, with only serotype 3 showing seroresponse rate <80%. Post-booster IgG GMCs and seroresponse rates exceeded primary series levels for all serotypes (except serotype 3). We conducted post-hoc analyses to assess the effects of age at first dose and primary-series intervals on IgG responses, controlling for vaccine and schedule by restricting the analysis to study arms using the PCV13 “3 + 1” schedule (Supplementary Fig. 16–19). Administering the first dose at 3 months of age resulted in considerably higher post-primary IgG responses across all serotypes, compared with starting at 1.5 or 2 months; for example, serotype 14 IgG GMCs were 16.4 (95% CI: 14.8–18.1) at 3 months vs 1.7 (1.6–1.9) at 2 months and 0.8 (0.5–1.1) at 6 weeks. Following the booster dose, higher IgG levels were observed for 7 of 13 serotypes for participants receiving first dose at 3 months than 1.5 or 2 months. Increasing the interval between primary doses from 1 to 2 months had serotype-specific effects: IgG responses increased for serotypes 6B, 14, 5, and 6A; remained similar for 9V, 18C, 19F, 23F, 1, and 7F; and decreased for 4, 3, and 19A. Post-booster IgG responses were higher for all serotypes with the 1-month interval.

Pooled estimates across four sensitivity analyses were consistent with the main meta-analysis (Supplementary Figs. 20, 21), confirming the robustness of our findings. We found no evidence of publication bias (Supplementary Figs. 22, 23).

## Discussion

Our study is the largest study to systematically and quantitatively evaluate immune responses in young children for five commonly used CRM197-based PCVs following alternative dosing regimens across different populations and epidemiologic contexts. Our findings demonstrate that IgG responses post pneumococcal vaccination vary by serotype, CRM197-based PCV product, vaccine schedule, and WHO region. These immunogenicity differences should be incorporated into transmission models and health economic evaluations, especially as countries adapt vaccination programs based on their local vaccine choices, schedules, serotype distributions and disease burden.

Overall, pooled serotype-specific IgG GMCs post-childhood-schedule for all VTs were above the WHO-defined protective threshold against IPD, consistent with previous reported overall vaccine effectiveness estimates of 80.7–86.0% against VT-IPD^20–22^. However, IgG responses varied considerably across serotypes. For some serotypes, such as serotype 3, lower IgG responses may partly explain its over-representation among vaccine failure cases^23^, suggesting a higher correlate of protection may be required, as demonstrated by Andrews and colleagues^9^. Consistently, multiple real-world studies have concluded little to no impact of PCV13 on reducing IPD attributed to serotype 3^24,25^, with a pooled effectiveness estimated at 63.5% (95% CI: 37.3–89.7%)^25^. In contrast, serotypes 19A and 19F exhibited relatively high IgG levels that substantially exceed the correlates of protection proposed by Andrews and colleagues^9^, yet are still frequently linked with vaccine failure^23^, implying factors beyond antibody levels—such as functionality or bacterial virulence—may contribute to protection. This finding suggests that vaccine effectiveness against disease outcomes varies by serotype, requiring definition of serotype-specific protective thresholds to accurately infer vaccine effectiveness.

A downward trend in IgG GMCs was generally observed with increasing valency of vaccine formulations for most serotypes after adjusting for assay effects. However, the IgG responses for PCV10-SII and PCV13 were similar based on limited evidence from AFR; additional evidence is required to confirm this finding. Previous studies similarly found that, compared with PCV7, higher-valency PCVs (PCV10-GSK and PCV13) had lower GMCs for most serotypes^18,19^. Given that CPS amounts per serotype were largely consistent across vaccines, this pattern is unlikely to be explained by differences in antigen amounts. This trend may reflect a potential trade-off between serotype coverage and immunogenicity, potentially due to antigenic competition and carrier-induced epitope suppression^26^. However, as no studies used the same laboratory methods to measure immune responses after PCV15 and PCV20 vaccination, these comparisons should be interpreted with caution. Overall, this downward trend in IgG GMCs with increasing valency highlights the importance of balancing broader vaccine serotype coverage and the magnitude of vaccine-induced IgG responses at the population level.

Vaccine schedules that included a booster dose (3 + 1, 2 + 1 or 1 + 1) consistently elicited higher IgG GMCs and seroresponse rates following completion of the childhood vaccination compared to a primary-only (3 + 0) schedule, regardless of serotypes and vaccine products. Deloria and colleagues^17^ also reported that booster doses substantially increased GMCs for all serotypes, with 2 + 1 schedule yielding higher IgG response than 3 + 0. Serosurveillance studies in several high-burden settings shows that, even with high coverage, population-level immunity under a 3 + 0 schedule can wane below the WHO-defined protective threshold beyond the first year of life^27,28^. Also, increased rates of vaccine failures have been reported in primary-only cohorts compared with those receiving booster-containing schedules^23,29^. These finding underscores the critical role of a booster dose in achieving sustained vaccine-induced IgG responses during and beyond the second year of life. However, experience from several African countries shows that 3 + 0 schedules with reasonable coverage have achieved substantial population-level protection^30,31^, indicating that countries introducing PCVs should consider local pneumococcal epidemiology and programmatic feasibility when choosing between schedules.

Regarding the primary series, a 3-dose schedule generally induced higher IgG responses than 2- or 1-dose schedule, although the difference between 3-dose and 2-dose was generally small, but was substantial for PCV20 recipients. Our findings partly align with an earlier review favouring 3-doses^17^, however that review only included limited serotypes. We further included post 1-dose responses and found that a single dose of PCV7/PCV13 produced IgG GMCs lower than the protective threshold for most serotypes, such as 6B, 6A, 9V and 23F. Overall, at least a 2-dose primary schedule is likely needed to induce sufficient antibody levels for short-term protection against most VTs. Notably, the largest differences were observed in serotypes 6B and 23F, for which two primary doses may not provide sufficient protection during the first year of life based on observed post-immunisation IgG responses, consistent with previous reviews^32,33^. These findings suggest that a 3-dose primary series may be required in settings where these serotypes predominate. For PCV20 recipients, the difference between 2- and 3-doses was remarkable, indicating suboptimal immunogenicity with a 2-dose schedule and supporting the need for three doses to achieve optimal protection with PCV20.

We additional evaluated the effects of age at first dose and the primary-series interval. A previous review has reported that administering the first dose at younger ages did not substantially affect post-primary GMCs^17^. In contrast, our data showed higher IgG levels when the first dose was given at 3 months than 2 or 1.5 months for both primary and booster responses. Our analysis was limited to study arms using PCV13 with a 3 + 1 schedule, and should be interpreted with caution. For the interval between primary doses, our results partly aligned with an earlier review^17^ in showing higher responses for serotype 6B and 14 with a 2-month interval than 1-month. However, that review assessed only a few serotypes. We further expanded the analysis to a larger number of serotypes and found substantial heterogeneity by serotype. As some serotypes seemed to favour longer intervals and others shorter ones, it is not possible to achieve the optimal response for all serotypes. For the booster dose, all serotypes showed higher responses after the 1-month interval, although the CIs largely overlapped. This previously undescribed pattern may reflect regional differences in schedule and warrants further investigation.

The 1 + 1 schedule—one primary dose followed by a booster—presents a unique immunological profile. Our findings indicate that post-booster IgG responses following a 1 + 1 schedule were higher or equivalent to those after 2- or 3-dose primary series (3 + 1 or 2 + 1), except for serotype 6B, but only limited to few studies. Real-world studies have shown that dropping a primary dose from a 2 + 1 schedule did not increase VT carriage and vaccine failure in the second year of life^34–36^. However, IgG responses were low after a single primary dose for most serotypes based on PCV7 and PCV13 data. Given the observed “downward trend” in immunogenicity with higher valency PCVs, post-primary responses after 1 + 1 schedules with PCV15 and PCV20 may be even lower, this requires confirmation. This divergence—low early immunogenicity but strong booster responses—may partly result from insufficient early protection that allows continued pneumococcal carriage during infancy, thereby enhancing the subsequent booster response. While the 1 + 1 schedule is promising in terms of simplification and affordability, its suitability should be carefully evaluated using transmission models that incorporate immunological parameters^37^, particularly in settings where early-life pneumococcal exposure is common.

We observed substantial regional variation of serotype-specific IgG responses post-childhood-schedule. IgG responses were generally highest in the WPR, followed by AFR and SEAR, and lowest in AMR and EUR, regardless of vaccine product. These findings are consistent with previous studies assessing the impact of geographic region on post-primary immunogenicity: Park and colleagues^19^ reported higher GMCs in Asia, Africa, and Latin America, while Choe and colleagues^16^ similarly found enhanced responses in WPR, SEAR, and AFR. Our study extends this evidence by including newer PCVs—PCV15, PCV20, and PCV10-SII—and observed similar trends, although evidence is limited to certain regions, with PCV10-SII only being evaluated in AFR. The regional variations may reflect differences in circulating serotypes and force of infection^2^. These findings suggest that regions with high IgG GMCs, such as WPR, may require fewer vaccine doses to achieve an equivalent level of population immunity to AMR or EUR, potentially resulting in substantial cost savings. The observed regional differences also highlight the importance of reviewing immunogenicity studies in the context of their specific settings as findings may not be routinely generalisable across all global regions.

Several limitations should be noted. First, PCV10-GSK was not included because it has different carrier proteins (Protein D, tetanus toxoid, and diphtheria toxoid), in contrast to all other PCVs in this review that use CRM197 exclusively. Although restricting analyses to CRM197-based PCVs enhances internal comparability, it limits the generalizability to all globally used PCV products. Second, functional OPA, a potentially better predictor of protection than serum IgG, was not assessed in our study because substantial variability in laboratory methods across studies would make direct comparisons unreliable^38^. In addition, OPA measurements generally require larger blood volumes and are therefore typically performed only in a subset of young children, resulting in limited and non-representative datasets across studies. Third, in our study, antibody levels were compared mainly against the WHO-defined IgG GMC putative protective threshold of 0.35 μg/mL, which serves as a regulator benchmark for protection against IPD following the post-primary infant series. Although this infant-derived threshold is widely used to report seroresponse rates and to facilitate comparison across PCV schedules, it should be interpreted with caution for booster-dose responses, as post-booster immunogenicity cannot be evaluated solely on this reference value. Also, because higher IgG levels are likely needed to protect against pneumococcal colonization and mucosal diseases, our findings should be interpreted accordingly. Moreover, given large number of studies included, full text review and data extraction were performed by a single reviewer and independently checked by a second reviewer, which may introduce a small risk of error but was considered a practical and commonly accepted approach. Finally, only English-language studies were included to ensure accurate data extraction, as English is the common language used by our team. This may have excluded relevant studies published in other languages, particularly from Asia and Africa where pneumococcal burden is highest, potentially introducing language bias.

Despite these limitations, our systematic review provided quantitative estimates of variations in vaccine immunogenicity by serotype, vaccine type, schedule and WHO region. These differences should be appropriately considered when evaluating vaccine programs. The observed downward trends in both IgG GMCs and seroresponse rates with increasing vaccine valency challenge the rationale of continually expanding PCV valency. Rather than accepting suboptimal immune responses as an inevitable trade-off, future vaccine development could benefit from novel approaches, such as optimizing conjugation strategies to enhance immunogenicity. Developing a standalone vaccine targeting problematic serotypes (e.g., serotypes 3 and 19A) for use alongside routine immunization schedules may also be a feasible strategy in settings with a substantial disease burden of these problematic serotypes.

## Methods

This systematic review and meta-analysis, reported in accordance with Preferred Reporting Items for Systematic Review and Meta-Analysis (PRISMA) 2020 guidelines (see Supplementary Table 1)^39^, investigated the immunogenicity of pneumococcal vaccination in children under 2 years. The study protocol was registered in the International Prospective Register of Systematic Reviews (PROSPERO) database (ID CRD42024484824).

### Search strategy and selection criteria

We formulated a search strategy using relevant keywords and medical subject heading terms for pneumococcal vaccination and immunogenicity. Five databases—EMBASE, MEDLINE, Web of Science Core Collection, Global Health, and Cochrane Central Register of Controlled Trials—were searched on May 09, 2024, with no date or study design restrictions, except for English language. An updated search was performed on Jan 07, 2025. Reference lists of included articles were screened manually for relevant studies. The full search strategy and results are provided in the Supplementary Tables 2–6.

All identified citations were imported to online systematic review software Covidence, and duplicate removal was performed. Title and abstract screening were undertaken independently by two reviewers (XC and ST) for assessment against the eligibility criteria. Only licensed PCVs using CRM197 as the carrier protein (PCV7, PCV13, PCV15, PCV20 and PCV10-SII) were eligible for inclusion. Given that carrier type and pre-existing anti-carrier immunity can substantially alter anti-CPS responses^26,40^, vaccines using different carrier proteins such as PCV10-GSK were excluded to avoid carrier-related immunologic heterogeneity. In addition, opsonophagocytic activity (OPA) data were excluded because cross-study methodological variability and the limited availability of OPA measurements within studies—typically restricted to small, randomly selected subsets due to serum volume requirements—precluded reliable comparison^38^. The full texts of potentially eligible studies were retrieved, and the articles were uploaded to Covidence. Full texts were assessed by one reviewer (XC) according to the predefined eligibility criteria, and a second reviewer (ST) independently checked a random subset of the full-text articles to ensure consistency. Differences at any stage were resolved through discussion with a third reviewer (PTC, DJP or JMcV).

Eligible studies met the following inclusion criteria: (1) Studies investigating any of the five licensed pneumococcal CRM197-based conjugate vaccines (PCV7, PCV13, PCV10-SII, PCV15, and PCV20; detailed characteristics are provided in the Supplementary Table 7) in healthy paediatric populations aged 2 years and under; (2) Studies reporting either antibody responses assessed by anti-polysaccharide IgG GMC and 95% confidence intervals (CIs) in micrograms per millilitre (µg/mL) or IgG seroresponse rates for at least one time point of a) between 4 and 6 weeks after the primary vaccination series, b) and/or 4 and 6 weeks after a booster vaccination. Immunoassays used for the quantitation of pneumococcal IgG antibodies in the included studies are detailed in Supplementary Table 8. Studies that measured antibody responses by radioimmunoassay or hemagglutination, studies focused on immunocompromised patients, studies in preterm infants only, studies related to maternal vaccination and related infant immune response, studies on infants born to HIV-infected mothers, whether prenatally infected or exposed but uninfected, studies with only OPA results, and studies with only salivary IgG response results were excluded.

Citations meeting inclusion criteria were grouped into “study groups”, defined as publications or abstracts arising from a single protocol, population, or surveillance system. Within each study group, we defined “study arms” as cohorts of children receiving a distinct immunisation schedule and/or PCV product. A study group could therefore include one or multiple study arms (see Supplementary Table 12 for full listings).

### Data extraction, outcomes, and quality analysis

A standardized data extraction form was developed to collect study and participant characteristics, vaccine product, schedule-related information, laboratory assay, and immunogenicity outcomes from each included study arm (see Supplementary Table 13 for the full variable list and completeness analysis). One reviewer (XC) extracted data for each study arm, a second reviewer (ST) checked a random subset for accuracy; and discrepancies were resolved through discussion or consultation with a third reviewer (PTC, DJP or JMcV). Authors were contacted to clarify any ambiguities or discrepancies.

In our study, vaccination schedules were classified as follows: “1 + 1” referred to one primary dose with one PCV booster dose, respectively; “2 + 1” referred to two primary doses with a booster; “3 + 0” and “3 + 1” referred to three primary doses without and with a booster; “0 + 1” represented one included study arm with only a single PCV dose given at 12 months of age. Additionally, “1-dose”, “2-dose”, and “3-dose” primary schedules referred to vaccination schedules consisting of one, two, and three primary doses, respectively. A booster dose was defined as a PCV dose administered between 9 and 18 months of age in infants who had completed a 1-, 2-, or 3-dose primary series.

Immunogenicity data were categorised by blood sample collection time in relation to administered vaccine doses. Our primary outcome was serotype-specific IgG GMCs at 30 days after completion of the childhood vaccination schedule (“post-childhood-schedule”). The childhood schedule is defined as the completion of the full infant/toddler PCV vaccination series, either with or without booster, depending on the assigned schedule. Additionally, serotype-specific IgG GMCs at 30 days post-primary series (“post 1-, 2-, 3-primary dose”) and at 30 days post-booster (“post-booster”) were also collected to allow comparisons across different vaccine schedules. Each study arm could have multiple immunogenicity outcomes if data were reported at more than one timepoint. The secondary outcome was serotype-specific IgG seroresponse rates at 30 days post-childhood-schedule, post-primary series, and post-booster, defined as the proportion of participants meeting the assay-specific protective threshold against IPD. To ensure consistency across results, 95% CIs for seroresponse rates were calculated using Fisher’s exact test, based on the reported number of seropositive participants and the total number of participants.

Each unique study group and study arm were assigned a distinct identifier to facilitate data processing and analysis. The WHO region codelist used in our study was retrieved from WHO Global Health Observatory metadata, and the country income group was retrieved from The World Bank. The schedule-related information was extracted directly from the study when available. If the age at first dose was not reported, we inferred the timing of each dose using the stated dosing schedule. For example, for participants receiving a “3 + 1” schedule with doses administered at 2, 4, 6, and 12 months, the age at first dose was assumed to be 2 months. The interval between primary doses was determined based on the vaccine dosing schedule and defined as the number of months between the first and second primary doses or between the second and third primary doses. When these intervals differed, their average was used. To standardise categorisation across studies, the timing of each dose and the interval between primary doses were rounded to the nearest two weeks.

The quality of included studies was assessed by two reviewers (XC and PC) jointly using modified versions of the Joanna Briggs Institute (JBI) critical appraisal tools for randomized controlled trials^41^, quasi-experimental studies^42^, and cohort studies^43^ according to the type of study (Supplementary Tables 9–11). Each study was assessed and classified as low-, moderate- or high-risk of bias; disagreements were solved by discussion (Supplementary Tables 14–16).

### Data analysis

Study arms were included in the main analysis if they met the following criteria: participants received their first primary dose at ≤4 months of age and last primary dose at ≤6 months of age; vaccines were administered intramuscularly; outcomes were measured using second- or third-generation ELISA (WHO ELISA), or assays bridged to WHO ELISA (see Supplementary Table 8); study arms were from study groups with low or moderate risk of bias.

Pooled mean serotype-specific IgG GMCs with 95% CIs and seroresponse rates were estimated across vaccine products, schedules and WHO regions. Random-effects models were used to analyse log-transformed (base-2) serotype-specific IgG GMCs and logit-transformed seroresponse rates, and weights were assigned to each study arm based on the inverse variance weighting method. Between-study variance was estimated using the Paule-Mandel method, with 95% CIs obtained by the Q-profile method. For the overall random effects estimates, 95% CIs were calculated using a Wald-type normal distribution. Heterogeneity between study arms was assessed using the inconsistency index (*I*^*2*^) statistic^44^. Four sensitivity analyses were conducted to evaluate the robustness of the results: 1) including study arms from studies with a high risk of bias; 2) including study arms that used first-generation ELISA, GSK’s 22F ELISA, ELISA without details and fluorescent multiplex immunoassay (FMIA); 3) including study arms with subcutaneous vaccine administration; 4) including study arms with late primary dosing (>4 months for first dose or >6 months for last dose). Publication bias was assessed using funnel plots. Data were analysed with R statistical software (version 4.3.3)^45^.

## Supplementary information

Supplementary information

## Data Availability

All data used in this study were obtained from publicly available sources, including population-level immune response data. No new individual participant data were collected for this research. Since the data are publicly accessible, any qualified researcher can access the datasets directly from the original sources. All data and full analysis code supporting the findings of this study is publicly available at the following repository: https://github.com/XINGHUI-CHEN/CRM-bases-PCV-infant-immunity-study.git. The repository provides the complete R code and data required to reproduce the analyses and figures reported in the manuscript.
