# Supplementary material for: Impact of CRM197-based conjugate vaccines, schedules, and regions on pneumococcal immunogenicity in young children: systematic review
Source: NPJ Vaccines. 2026 Mar 6;11:87. doi: 10.1038/s41541-026-01395-y (PMC13096133; doi:10.1038/s41541-026-01395-y)
Supplement: Supplementary file 1 — Supplementary information [file 41541_2026_1395_MOESM1_ESM.pdf]

**Supplementary appendix:**

# Impact of CRM197-based conjugate vaccines, schedules, and regions on pneumococcal immunogenicity in young children: systematic review

Xinghui Chen[a,*], Sarah Tavlian[a], Kylie S. Carville[a,b], Nefel Tellioglu[a], Violeta Spirkoska[a], Natalie Carvalho[d], David J. Price[a,c], Patricia T. Campbell[a], and Jodie McVernon[a,b,*]

[a]Department of Infectious Diseases, University of Melbourne, at the Peter Doherty Institute for Infection and Immunity, Melbourne, Australia

[b]Victorian Infectious Diseases Reference Laboratory, The Royal Melbourne Hospital, at the Peter Doherty Institute for Infection and Immunity, Melbourne, Australia

[c]Centre for Epidemiology and Biostatistics, Melbourne School of Population and Global Health, The University of Melbourne, Melbourne, Australia

[d]Centre for Health Policy, Melbourne School of Population and Global Health, The University of Melbourne, Melbourne, Australia

*Corresponding authors: Xinghui Chen (xinghuichen.research@gmail.com) and Jodie McVernon (j.mcvernon@unimelb.edu.au)

## Contents


Supplementary Tables ........ 1

Supplementary Table 1. PRISMA 2020 Checklist ........ 1

Supplementary Table 2. Search strategy and results in Embase ........ 1

Supplementary Table 3. Search strategy and results in Medline ........ 2

Supplementary Table 4. Search strategy and results in Web of Science ........ 3

Supplementary Table 5. Search strategy and results in Global Health ........ 4

Supplementary Table 6. Search strategy and results in Cochrane Central Register of Controlled Trials ........ 5

Supplementary Table 7. Included pneumococcal conjugate vaccines and their characteristics ........ 6

Supplementary Table 8. Included assays for the quantitation of pneumococcal IgG antibody and assay-specific protective thresholds ........ 6

Supplementary Table 9. The original and modified JBI tools for randomized clinical trials (RCTs) ........ 7

Supplementary Table 10. The original and modified JBI tools for quasi experimental study ........ 7

Supplementary Table 11. The original and modified JBI tools for cohort study ........ 7

Supplementary Table 12. Summary of studies included in immunogenicity analysis ........ 9

Supplementary Table 13. Variable list and completeness analysis ........ 22

Supplementary Table 14. Risk of bias assessment according to the modified JBI tool for randomized controlled trial (RCT) ........ 23

Supplementary Table 15. Risk of bias assessment according to the modified JBI tool for quasi experimental study ........ 25

Supplementary Table 16. Risk of bias assessment according to the modified JBI tool for cohort study ........ 26

Supplementary Table 17. Distribution of included study arms by vaccine products and countries conducted 27

Supplementary Table 18. Detailed breakdown of countries involved in “Multi-countries” studies and corresponding number of study arms ........ 27

Supplementary Table 19. Summary of study arm inclusion and exclusion for the primary analysis ........ 28

Supplementary Table 20. Number of study arms reporting IgG geometric mean concentrations (IgG GMCs) and seroresponse rates post-childhood-schedule by vaccine product ........ 28

Supplementary Table 21. Number of study arms reporting IgG geometric mean concentrations (IgG GMCs) and seroresponse rates post-childhood-schedule by vaccine product and schedule ........ 28

Supplementary Table 22. Number of study arms reporting IgG geometric mean concentrations (IgG GMCs) and seroresponse rates post “3+1” vaccination schedule by vaccine product and WHO region ........ 29

Supplementary Table 23. Number of study arms reporting IgG geometric mean concentrations (IgG GMCs) and seroresponse rates post different timepoints by vaccine product ........ 29

Supplementary Table 24. Summary of the meta-analysis results of anti-pneumococcal IgG geometric mean concentrations (GMCs, μg/mL) post-childhood-schedule by vaccine product and serotype ........ 30

Supplementary Table 25. Summary of the meta-analysis results of anti-pneumococcal IgG seroresponse rates (%) post-childhood-schedule by vaccine product ........ 30

Supplementary Table 26. Summary of the meta-analysis results of anti-pneumococcal IgG geometric mean concentrations (IgG GMCs, μg/mL) post-childhood-schedule by vaccine product and schedule ........ 31

Supplementary Table 27. Summary of the meta-analysis results of anti-pneumococcal IgG seroresponse rates (%) post-childhood-schedule by vaccine product and schedule ........ 32

Supplementary Table 28. Summary of the meta-analysis results of anti-pneumococcal IgG geometric mean concentrations (IgG GMCs, μg/mL) post “3+1” vaccination schedule by vaccine product and region ........ 33

Supplementary Table 29. Summary of the meta-analysis results of anti-pneumococcal IgG seroresponse rates (%) post "3+1" vaccination schedule by vaccine product and region ..... 36

Supplementary Table 30. Summary of the meta-analysis results of anti-pneumococcal IgG geometric mean concentrations (IgG GMCs, μg/mL) by vaccine product and timepoint ..... 37

Supplementary Table 31. Summary of the meta-analysis results of anti-pneumococcal IgG seroresponse rates (%) by vaccine product and timepoint..... 39

Supplementary Figures ..... 41

Supplementary Figure 1. Temporal distribution of study arms based on study start year ..... 41

Supplementary Figure 2. World map of study sites by number of study arms ..... 41

Supplementary Figure 3. Pneumococcal post-childhood-schedule IgG GMCs (μg/mL) by serotype and vaccine product for all vaccine-covered serotypes ..... 42

Supplementary Figure 4. Pneumococcal post-childhood-schedule seroresponse rates (%) by serotype and vaccine product ..... 42

Supplementary Figure 5. Pneumococcal post-childhood-schedule IgG GMCs (μg/mL) by serotype and vaccine product for different assays ..... 43

Supplementary Figure 6. Pneumococcal post-childhood-schedule IgG GMCs (μg/mL) for PCV10-SII vs PCV13 by serotype in African Region ..... 44

Supplementary Figure 7. Pneumococcal post-childhood-schedule IgG GMC (μg/mL) by serotype, vaccine product and vaccine schedule ..... 45

Supplementary Figure 8. Pneumococcal post-childhood-schedule seroresponse rates (%) by serotype, vaccine product and vaccine schedule ..... 46

Supplementary Figure 9. Pneumococcal IgG GMC (μg/mL) post "3+1" vaccination schedule by serotype, vaccine product and region ..... 47

Supplementary Figure 10. Pneumococcal seroresponse rates (%) post "3+1" vaccination schedule by serotype, vaccine product and region ..... 48

Supplementary Figure 11. Serotype-specific pneumococcal IgG GMC (μg/mL) post PCV13 vaccination by vaccine schedule and region ..... 49

Supplementary Figure 12. Serotype-specific pneumococcal seroresponse rates (%) post PCV13 vaccination by vaccine schedule and region ..... 50

Supplementary Figure 13. Serotype-specific pneumococcal IgG GMCs (μg/mL) by vaccine product and timepoint ..... 51

Supplementary Figure 14. Post-primary series serotype-specific pneumococcal IgG GMCs (μg/mL) by vaccine product and timepoint ..... 52

Supplementary Figure 15. Serotype-specific pneumococcal seroresponse rates (%) by vaccine product and timepoint ..... 53

Supplementary Figure 16. Serotype-specific pneumococcal IgG GMCs (μg/mL) post PCV13 vaccination with "3+1" schedule by timepoint and age of first dose ..... 54

Supplementary Figure 17. Serotype-specific pneumococcal seroresponse rates (%) post PCV13 vaccination with "3+1" schedule by timepoint and age of first dose ..... 55

Supplementary Figure 18. Serotype-specific pneumococcal IgG GMCs (μg/mL) post PCV13 vaccination with "3+1" schedule by timepoint and interval between primary doses ..... 56

Supplementary Figure 19. Serotype-specific pneumococcal seroresponse rates (%) post PCV13 vaccination with "3+1" schedule by timepoint and interval between primary doses ..... 57

Supplementary Figure 20. Sensitivity analysis of pneumococcal post-childhood-schedule IgG GMCs (μg/mL) by serotype and vaccine product (c.f. Fig 2 and Fig S3 which excludes these studies) ..... 58

Supplementary Figure 21. Sensitivity analysis of pneumococcal post-childhood-schedule seroresponse rates (%) by serotype and vaccine product (c.f. Fig S5 which excludes these studies) ........ 59
Supplementary Figure 22. The funnel plot analysis for PCV7 ........ 60
Supplementary Figure 23. The funnel plot analysis for PCV13 ........ 61
Supplementary References ........ 62

## Supplementary Tables

### Supplementary Table 1. PRISMA 2020 Checklist

| Section and Topic | Item # | Checklist item | Location where item is reported |
|---|---|---|---|
| **TITLE** | | | |
| Title | 1 | Identify the report as a systematic review. | Describe in the title |
| **ABSTRACT** | | | |
| Abstract | 2 | See the PRISMA 2020 for Abstracts checklist. | The eligibility criteria, risk of bias and limitations are not well described in abstract section.<br>Background, objectives, information source, synthesis methods, number of included studies, results for main outcomes and interpretation have described well. |
| **INTRODUCTION** | | | |
| Rationale | 3 | Describe the rationale for the review in the context of existing knowledge. | Well describe in introduction |
| Objectives | 4 | Provide an explicit statement of the objective(s) or question(s) the review addresses. | Well describe in introduction |
| **METHODS** | | | |
| Eligibility criteria | 5 | Specify the inclusion and exclusion criteria for the review and how studies were grouped for the syntheses. | Well describe in method section (in Search strategy and selection criteria part) |
| Information sources | 6 | Specify all databases, registers, websites, organisations, reference lists and other sources searched or consulted to identify studies. Specify the date when each source was last searched or consulted. | Well describe in method section (in Search strategy and selection criteria part) and Supplementary Table 1-5) |
| Search strategy | 7 | Present the full search strategies for all databases, registers and websites, including any filters and limits used. | Well describe in method section (in Search strategy and selection criteria part) and Supplementary Table 1-5) |
| Selection process | 8 | Specify the methods used to decide whether a study met the inclusion criteria of the review, including how many reviewers screened each record and each report retrieved, whether they worked independently, and if applicable, details of automation tools used in the process. | Well describe in method section (in Search strategy and selection criteria part) |
| Data collection process | 9 | Specify the methods used to collect data from reports, including how many reviewers collected data from each report, whether they worked independently, any processes for obtaining or confirming data from study investigators, and if applicable, details of automation tools used in the process. | Well describe in method section (in Data extraction, outcomes, and quality analysis part) |
| Data items | 10a | List and define all outcomes for which data were sought. Specify whether all results that were compatible with each outcome domain in each study were sought (e.g. for all measures, time points, analyses), and if not, the methods used to decide which results to collect. | Well describe in method section (in Data extraction, outcomes, and quality analysis part) |
| | 10b | List and define all other variables for which data were sought (e.g. participant and intervention characteristics, funding sources). Describe any assumptions made about any missing or unclear information. | Well describe in method section (in Data extraction, outcomes, and quality analysis part) and Supplementary Table 14 |
| Study risk of bias assessment | 11 | Specify the methods used to assess risk of bias in the included studies, including details of the tool(s) used, how many reviewers assessed each study and whether they worked independently, and if applicable, details of automation tools used in the process. | Well describe in method section and Supplementary Table 8-10 |
| Effect measures | 12 | Specify for each outcome the effect measure(s) (e.g. risk ratio, mean difference) used in the synthesis or presentation of results. | Well describe in method section (Data Analysis part) |

| Section and Topic | Item # | Checklist item | Location where item is reported |
|---|---|---|---|
| Synthesis methods | 13a | Describe the processes used to decide which studies were eligible for each synthesis (e.g. tabulating the study intervention characteristics and comparing against the planned groups for each synthesis (item #5)). | Well describe in method section (Data Analysis part) the criteria of study arms included in the main analysis |
| | 13b | Describe any methods required to prepare the data for presentation or synthesis, such as handling of missing summary statistics, or data conversions. | Well describe in method section (Data Analysis part) |
| | 13c | Describe any methods used to tabulate or visually display results of individual studies and syntheses. | N/A, but all individual studies were all listed in Supplementary Table 12 |
| | 13d | Describe any methods used to synthesize results and provide a rationale for the choice(s). If meta-analysis was performed, describe the model(s), method(s) to identify the presence and extent of statistical heterogeneity, and software package(s) used. | Well describe in method section (Data Analysis part) |
| | 13e | Describe any methods used to explore possible causes of heterogeneity among study results (e.g. subgroup analysis, meta-regression). | Well describe in method section (Data Analysis part) |
| | 13f | Describe any sensitivity analyses conducted to assess robustness of the synthesized results. | Well describe in method section about four specific sensitivity analysis that were conducted |
| Reporting bias assessment | 14 | Describe any methods used to assess risk of bias due to missing results in a synthesis (arising from reporting biases). | Publication bias assessment was clearly described |
| Certainty assessment | 15 | Describe any methods used to assess certainty (or confidence) in the body of evidence for an outcome. | Well describe in method section (Data Analysis part) |
| **RESULTS** | | | |
| Study selection | 16a | Describe the results of the search and selection process, from the number of records identified in the search to the number of studies included in the review, ideally using a flow diagram. | Well describe in result section and in flow chart |
| | 16b | Cite studies that might appear to meet the inclusion criteria, but which were excluded, and explain why they were excluded. | Well describe in result section and in flow chart |
| Study characteristics | 17 | Cite each included study and present its characteristics. | Well describe in Supplementary Table 12 |
| Risk of bias in studies | 18 | Present assessments of risk of bias for each included study. | Well describe in Supplementary Table 12 and 14-16 |
| Results of individual studies | 19 | For all outcomes, present, for each study: (a) summary statistics for each group (where appropriate) and (b) an effect estimate and its precision (e.g. confidence/credible interval), ideally using structured tables or plots. | Well describe in Supplementary Table 12 |
| Results of syntheses | 20a | For each synthesis, briefly summarise the characteristics and risk of bias among contributing studies. | Well describe in Table 1 and Supplementary Table 12 |
| | 20b | Present results of all statistical syntheses conducted. If meta-analysis was done, present for each the summary estimate and its precision (e.g. confidence/credible interval) and measures of statistical heterogeneity. If comparing groups, describe the direction of the effect. | Well describe for both point estimates and 95% confidence intervals and also the measures of statistical heterogeneity |
| | 20c | Present results of all investigations of possible causes of heterogeneity among study results. | Subgroup analysis to explore the possible causes of heterogeneity among study results |
| | 20d | Present results of all sensitivity analyses conducted to assess the robustness of the synthesized results. | Well describe in result section and results for sensitivity analyses were provided in Supplementary figure 20-21 |
| Reporting biases | 21 | Present assessments of risk of bias due to missing results (arising from reporting biases) for each synthesis assessed. | Funnel plots |
| Certainty of evidence | 22 | Present assessments of certainty (or confidence) in the body of evidence for each outcome assessed. | Well describe in result section, figure 2-4 |
| **DISCUSSION** | | | |

| Section and Topic | Item # | Checklist item | Location where item is reported |
|---|---|---|---|
| Discussion | 23a | Provide a general interpretation of the results in the context of other evidence. | Well describe in Discussion section |
| | 23b | Discuss any limitations of the evidence included in the review. | Well describe in Discussion section |
| | 23c | Discuss any limitations of the review processes used. | Well describe in Discussion section |
| | 23d | Discuss implications of the results for practice, policy, and future research. | Well describe in Discussion section |
| **OTHER INFORMATION** | | | |
| Registration and protocol | 24a | Provide registration information for the review, including register name and registration number, or state that the review was not registered. | Well describe in method section. The study protocol was registered in the International Prospective Register of Systematic Reviews (PROSPERO) database (ID CRD42024484824). |
| | 24b | Indicate where the review protocol can be accessed, or state that a protocol was not prepared. | Well describe in method section |
| | 24c | Describe and explain any amendments to information provided at registration or in the protocol. | N/A |
| Support | 25 | Describe sources of financial or non-financial support for the review, and the role of the funders or sponsors in the review. | Well describe in Declaration statements (Acknowledgements) |
| Competing interests | 26 | Declare any competing interests of review authors. | Well describe in Declaration statements (Competing Interests) |
| Availability of data, code and other materials | 27 | Report which of the following are publicly available and where they can be found: template data collection forms; data extracted from included studies; data used for all analyses; analytic code; any other materials used in the review. | Well describe in Declaration statements (Data Availability and Code Availability) |

## Supplementary Table 2. Search strategy and results in Embase

| ID | Embase | Results up to 2024-05-09 | Results up to 2025-01-07 |
|---|---|---|---|
| 1 | Streptococcus pneumoniae/ or pneumococcal infection/ or ("streptococcus pneumonia*" or pneumococc* or "s pneumonia*" or "strep pneumonia*" or "streptococcal pneumonia*").ti,ab,kf. | 85476 | 88173 |
| 2 | exp vaccine/ or exp immunization/ or (vaccin* or immuniz* or immunis*).ti,ab,kf. | 842499 | 876156 |
| 3 | **1 and 2** | 28767 | 29916 |
| 4 | Pneumococcus vaccine/ or (pneumococc* adj5 vaccin*).ti,ab,kf. | 28822 | 29865 |
| 5 | (7vpcv or "7v pcv" or pcv7 or "pcv 7" or heptavalent or "7 valent" or 7valent or pncrm7 or "pncrm 7" or 7vpnc or 7vcrm or "seven valent" or prevnar or prevenar or prevnar7 or prevenar7).ti,ab,kf. | 3894 | 3965 |
| 6 | (10vpcv or "10v pcv" or pcv10 or "pcv 10" or "10 valent" or 10valent or "ten valent" or pneumosil or "SIIPL PCV" or "SII Pneumosil").ti,ab,kf. | 1144 | 1192 |
| 7 | (13vpcv or "13v pcv" or pcv13 or "pcv 13" or 13vcrm or "13 valent" or 13valent or "thirteen valent" or prevenar13 or prevnar13).ti,ab,kf. | 4049 | 4269 |
| 8 | (15vpcv or "15v pcv" or pcv15 or "pcv 15" or "15 valent" or 15valent or "fifteen valent" or vaxneuvance or v114).ti,ab,kf. | 318 | 385 |
| 9 | (20vpcv or "20v pcv" or pcv20 or "pcv 20" or "20 valent" or 20valent or "twenty valent" or prevnar20 or prevenar20 or apexxnar).ti,ab,kf. | 237 | 340 |
| 10 | (21vpcv or "21v pcv" or pcv21 or "pcv 21" or "21 valent" or 21valent or "twenty one valent" or v116).ti,ab,kf. | 57 | 85 |
| 11 | (ppv23 or "ppv 23" or 23vppv or "23v ppv" or ppsv23 or "ppsv 23" or "23 valent" or 23valent or pneumovax or "pneumovax 23" or pneumovax23 or "pneumo 23").ti,ab,kf. | 3184 | 3286 |
| 12 | **3 or 4 or 5 or 6 or 7 or 8 or 9 or 10 or 11** | 35885 | 37277 |
| 13 | exp immunity/ or antibody/ or immunoglobulin G/ or (immun* or antibod* or "immunoglobulin G" or IgG or opsonophagocyt* or OPA).ti,ab,kf. | 5228712 | 5423693 |
| 14 | **12 and 13** | 19951 | 20743 |
| 15 | (exp in vitro study/ or exp animal experiment/ or exp animal/ or exp juvenile animal/ or adult animal/ or animal cell/ or animal tissue/ or nonhuman/ or animal model/ or exp invertebrate/ or exp plant/ or exp fungus/) not (exp human/ or human experiment/) | 9195544 | 9406874 |
| 16 | (("in vitro" or animal or animals or canine* or dog or dogs or cat or cats or feline or hamster* or lamb or lambs or mice or ferret* or primate* or macaque* or monkey or monkeys or mouse or murine or swine or pig or pigs or piglet* or porcine or rabbit* or rat or rats or rodent* or sheep* or bovine or cow or cows or horse or horses or poultry or chick or chicken* or turkey* or avian* or vertebrate* or veterinary* or plant* or fung*) not (human* or patient* or people or mankind*)).ti,ab,kf. | 6175695 | 6325725 |
| 17 | **15 or 16** | 10347740 | 10601649 |
| 18 | **14 not 17** | 17417 | 18143 |
| 19 | limit 18 to ("review" or editorial) | 3891 | 3993 |
| 20 | **18 not 19** | 13526 | 14150 |
| 21 | Language: English | 12702 | 13316 |
| | limit 21 to dc=20240510-20250107 (for update search only) | - | 583 |

## Supplementary Table 3. Search strategy and results in Medline

| ID | Medline (Ovid) | Results up to 2024-05-09 | Results up to 2025-01-07 |
|---|---|---|---|
| 1 | Streptococcus pneumoniae/ or Pneumococcal Infections/ or ("streptococcus pneumonia*" or pneumococc* or "s pneumonia*" or "strep pneumonia*" or "streptococcal pneumonia*").ti,ab,kf. | 53363 | 54390 |
| 2 | exp Vaccines/ or exp Immunization/ or (vaccin* or immuniz* or immunis*).ti,ab,kf. | 630284 | 650985 |
| 3 | **1 and 2** | 18252 | 18767 |
| 4 | Pneumococcal Vaccines/ or (pneumococc* adj5 vaccin*).ti,ab,kf. | 14557 | 14971 |
| 5 | (7vpcv or "7v pcv" or pcv7 or "pcv 7" or heptavalent or "7 valent" or 7valent or pncrm7 or "pncrm 7" or 7vpnc or 7vcrm or "seven valent" or prevnar or prevenar or prevnar7 or prevenar7).ti,ab,kf. | 2938 | 2971 |
| 6 | (10vpcv or "10v pcv" or pcv10 or "pcv 10" or "10 valent" or 10valent or "ten valent" or pneumosil or "SIIPL PCV" or "SII Pneumosil").ti,ab,kf. | 893 | 929 |
| 7 | (13vpcv or "13v pcv" or pcv13 or "pcv 13" or 13vcrm or "13 valent" or 13valent or "thirteen valent" or prevenar13 or prevnar13).ti,ab,kf. | 2823 | 2955 |
| 8 | (15vpcv or "15v pcv" or pcv15 or "pcv 15" or "15 valent" or 15valent or "fifteen valent" or vaxneuvance or v114).ti,ab,kf. | 216 | 259 |
| 9 | (20vpcv or "20v pcv" or pcv20 or "pcv 20" or "20 valent" or 20valent or "twenty valent" or prevnar20 or prevenar20 or apexxnar).ti,ab,kf. | 180 | 231 |
| 10 | (21vpcv or "21v pcv" or pcv21 or "pcv 21" or "21 valent" or 21valent or "twenty one valent" or v116).ti,ab,kf. | 30 | 47 |
| 11 | (ppv23 or "ppv 23" or 23vppv or "23v ppv" or ppsv23 or "ppsv 23" or "23 valent" or 23valent or pneumovax or "pneumovax 23" or pneumovax23 or "pneumo 23").ti,ab,kf. | 2075 | 2138 |
| 12 | **3 or 4 or 5 or 6 or 7 or 8 or 9 or 10 or 11** | 19162 | 19693 |
| 13 | exp Immunity/ or Immunoglobulin G/ or Antibodies/ or (immun* or antibod* or "immunoglobulin G" or IgG or opsonophagocyt* or OPA).ti,ab,kf. | 3545895 | 3663361 |
| 14 | **12 and 13** | 10196 | 10488 |
| 15 | (exp Animals/ or Disease Models, Animal/ or exp Animal Experimentation/ or exp Plants/ or exp Fungi/) not (exp Humans/ or Human Experimentation/) | 5659448 | 5746803 |
| 16 | (("in vitro" or animal or animals or canine* or dog or dogs or cat or cats or feline or hamster* or lamb or lambs or mice or ferret* or primate* or macaque* or monkey or monkeys or mouse or murine or swine or pig or pigs or piglet* or porcine or rabbit* or rat or rats or rodent* or sheep* or bovine or cow or cows or horse or horses or poultry or chick or chicken* or turkey* or avian* or vertebrate* or veterinary* or plant* or fung*) not (human* or patient* or people or mankind*)).ti,ab,kf. | 5135215 | 5261822 |
| 17 | **15 or 16** | 7264974 | 7425755 |
| 18 | **14 not 17** | 8560 | 8830 |
| 19 | limit 18 to ("review" or editorial) | 1643 | 1690 |
| 20 | **18 not 19** | 6917 | 7140 |
| 21 | limit 20 to english language | 6507 | 6721 |
| | limit 21 to dt=20240510-20250107 (for update search only) | - | 214 |

## Supplementary Table 4. Search strategy and results in Web of Science

| ID | Web of Science | Results up to 2024-05-13 | Results up to 2025-01-07 |
|---|---|---|---|
| 1 | TS=("streptococcus pneumonia*" or pneumococc* or "s pneumonia*" or "strep pneumonia*" or "streptococcal pneumonia*") | 60335 | 62504 |
| 2 | TS=(vaccin* or immuniz* or immunis*) | 598169 | 632305 |
| 3 | **#1 and #2** | 20934 | 21774 |
| 4 | TS=(pneumococc* NEAR/5 vaccin*) | 15298 | 15900 |
| 5 | TS=(7vpcv or "7v pcv" or pcv7 or "pcv 7" or heptavalent or "7 valent" or 7valent or pncrm7 or "pncrm 7" or 7vpnc or 7vcrm or "seven valent" or prevnar or prevenar or prevnar7 or prevenar7) | 3122 | 3164 |
| 6 | TS=(10vpcv or "10v pcv" or pcv10 or "pcv 10" or "10 valent" or 10valent or "ten valent" or pneumosil or "SIIPL PCV" or "SII Pneumosil") | 920 | 965 |
| 7 | TS=(13vpcv or "13v pcv" or pcv13 or "pcv 13" or 13vcrm or "13 valent" or 13valent or "thirteen valent" or prevenar13 or prevnar13) | 2971 | 3126 |
| 8 | TS=(15vpcv or "15v pcv" or pcv15 or "pcv 15" or "15 valent" or 15valent or "fifteen valent" or vaxneuvance or v114) | 222 | 263 |
| 9 | TS=(20vpcv or "20v pcv" or pcv20 or "pcv 20" or "20 valent" or 20valent or "twenty valent" or prevnar20 or prevenar20 or apexxnar) | 180 | 236 |
| 10 | TS=(21vpcv or "21v pcv" or pcv21 or "pcv 21" or "21 valent" or 21valent or "twenty one valent" or v116) | 27 | 41 |
| 11 | TS=(ppv23 or "ppv 23" or 23vppv or "23v ppv" or ppsv23 or "ppsv 23" or "23 valent" or 23valent or pneumovax or "pneumovax 23" or pneumovax23 or "pneumo 23") | 2047 | 2146 |
| 12 | **#3 or #4 or #5 or #6 or #7 or #8 or #9 or #10 or #11** | 21608 | 22480 |
| 13 | TS=(immun* or antibod* or "immunoglobulin G" or IgG or opsonophagocyt* or OPA) | 3885130 | 4101149 |
| 14 | **#12 and #13** | 12309 | 12844 |
| 15 | TS=(("in vitro" or animal or animals or canine* or dog or dogs or cat or cats or feline or hamster* or lamb or lambs or mice or ferret* or primate* or macaque* or monkey or monkeys or mouse or murine or swine or pig or pigs or piglet* or porcine or rabbit* or rat or rats or rodent* or sheep* or bovine or cow or cows or horse or horses or poultry or chick or chicken* or turkey* or avian* or vertebrate* or veterinary* or plant* or fung*) not (human* or patient* or people or mankind*)) | 8352238 | 8729305 |
| 16 | **#14 not #15** | 10742 | 11198 |
| 17 | **#14 not #15** and **Review Article** or **Editorial Material** (Exclude – Document Types) and **English** (Languages) | 8498 | 8905 |
| | **#17 and LD=(2024-05-14 to 2025-01-07)** (for update search only) | - | 257 |

## Supplementary Table 5. Search strategy and results in Global Health

| ID | Global Health (since 1973) | Results up to 2024-05-13 | Results up to 2025-01-07 |
|---|---|---|---|
| 1 | Streptococcus pneumoniae/ or ("streptococcus pneumonia*" or pneumococc* or "s pneumonia*" or "strep pneumonia*" or "streptococcal pneumonia*").ti,ab,id. | 21041 | 22042 |
| 2 | exp vaccines/ or exp immunization/ or (vaccin* or immuniz* or immunis*).ti,ab,id. | 208091 | 209029 |
| 3 | **1 and 2** | 9489 | 9512 |
| 4 | (pneumococc* adj5 vaccin*).ti,ab,id. | 7157 | 7227 |
| 5 | (7vpcv or "7v pcv" or pcv7 or "pcv 7" or heptavalent or "7 valent" or 7valent or pncrm7 or "pncrm 7" or 7vpnc or 7vcrm or "seven valent" or prevnar or prevenar or prevnar7 or prevenar7).ti,ab,id. | 2198 | 2224 |
| 6 | (10vpcv or "10v pcv" or pcv10 or "pcv 10" or "10 valent" or 10valent or "ten valent" or pneumosil or "SIIPL PCV" or "SII Pneumosil").ti,ab,id. | 642 | 683 |
| 7 | (13vpcv or "13v pcv" or pcv13 or "pcv 13" or 13vcrm or "13 valent" or 13valent or "thirteen valent" or prevenar13 or prevnar13).ti,ab,id. | 1982 | 2082 |
| 8 | (15vpcv or "15v pcv" or pcv15 or "pcv 15" or "15 valent" or 15valent or "fifteen valent" or vaxneuvance or v114).ti,ab,id. | 118 | 138 |
| 9 | (20vpcv or "20v pcv" or pcv20 or "pcv 20" or "20 valent" or 20valent or "twenty valent" or prevnar20 or prevenar20 or apexxnar).ti,ab,id. | 109 | 138 |
| 10 | (21vpcv or "21v pcv" or pcv21 or "pcv 21" or "21 valent" or 21valent or "twenty one valent" or v116).ti,ab,id. | 13 | 17 |
| 11 | (ppv23 or "ppv 23" or 23vppv or "23v ppv" or ppsv23 or "ppsv 23" or "23 valent" or 23valent or pneumovax or "pneumovax 23" or pneumovax23 or "pneumo 23").ti,ab,id. | 1198 | 1233 |
| 12 | **3 or 4 or 5 or 6 or 7 or 8 or 9 or 10 or 11** | 9517 | 9637 |
| 13 | exp immunity/ or antibodies/ or IgG/ or (immun* or antibod* or "immunoglobulin G" or IgG or opsonophagocyt* or OPA).ti,ab,id. | 670571 | 671674 |
| 14 | **12 and 13** | 5049 | 5143 |
| 15 | (in vitro/ or animal experiments/ or animals/ or animal tissues/ or animal models/ or invertebrates/ or plants/ or fungi/) not (man/ or human diseases/) | 1272514 | 1274215 |
| 16 | (("in vitro" or animal or animals or canine* or dog or dogs or cat or cats or feline or hamster* or lamb or lambs or mice or ferret* or primate* or macaque* or monkey or monkeys or mouse or murine or swine or pig or pigs or piglet* or porcine or rabbit* or rat or rats or rodent* or sheep* or bovine or cow or cows or horse or horses or poultry or chick or chicken* or turkey* or avian* or vertebrate* or veterinary* or plant* or fung*) not (human* or patient* or people or mankind*)).ti,ab,id. | 1003594 | 1005224 |
| 17 | **15 or 16** | 1563115 | 1564815 |
| 18 | **14 not 17** | 4703 | 4812 |
| 19 | limit 18 to editorial | 7 | 3 |
| 20 | **18 not 19** | 4696 | 4809 |
| 21 | limit 20 to english language | 4425 | 4525 |
| | limit 21 to yr="2024 -Current" (for update search only) | - | 115 |

## Supplementary Table 6. Search strategy and results in Cochrane Central Register of Controlled Trials

| ID | Cochrane Central Register of Controlled Trials | Results up to 2024-05-13 | Results up to 2025-01-07 |
|---|---|---|---|
| 1 | Streptococcus pneumoniae/ or Pneumococcal Infections/ or ("streptococcus pneumonia*" or pneumococc* or "s pneumonia*" or "strep pneumonia*" or "streptococcal pneumonia*").ti,ab,kf. | 3627 | 3751 |
| 2 | exp Vaccines/ or exp Immunization/ or (vaccin* or immuniz* or immunis*).ti,ab,kf. | 34575 | 36105 |
| 3 | **1 and 2** | 2340 | 2443 |
| 4 | Pneumococcal Vaccines/ or (pneumococc* adj5 vaccin*).ti,ab,kf. | 2105 | 2192 |
| 5 | (7vpcv or "7v pcv" or pcv7 or "pcv 7" or heptavalent or "7 valent" or 7valent or pncrm7 or "pncrm 7" or 7vpnc or 7vcrm or "seven valent" or prevnar or prevenar or prevnar7 or prevenar7).ti,ab,kf. | 681 | 696 |
| 6 | (10vpcv or "10v pcv" or pcv10 or "pcv 10" or "10 valent" or 10valent or "ten valent" or pneumosil or "SIIPL PCV" or "SII Pneumosil").ti,ab,kf. | 203 | 213 |
| 7 | (13vpcv or "13v pcv" or pcv13 or "pcv 13" or 13vcrm or "13 valent" or 13valent or "thirteen valent" or prevenar13 or prevnar13).ti,ab,kf. | 580 | 610 |
| 8 | (15vpcv or "15v pcv" or pcv15 or "pcv 15" or "15 valent" or 15valent or "fifteen valent" or vaxneuvance or v114).ti,ab,kf. | 118 | 125 |
| 9 | (20vpcv or "20v pcv" or pcv20 or "pcv 20" or "20 valent" or 20valent or "twenty valent" or prevnar20 or prevenar20 or apexxnar).ti,ab,kf. | 50 | 65 |
| 10 | (21vpcv or "21v pcv" or pcv21 or "pcv 21" or "21 valent" or 21valent or "twenty one valent" or V116).ti,ab,kf. | 23 | 30 |
| 11 | (ppv23 or "ppv 23" or 23vppv or "23v ppv" or ppsv23 or "ppsv 23" or "23 valent" or 23valent or pneumovax or "pneumovax 23" or pneumovax23 or "pneumo 23").ti,ab,kf. | 544 | 562 |
| 12 | **3 or 4 or 5 or 6 or 7 or 8 or 9 or 10 or 11** | 2487 | 2594 |
| 13 | exp Immunity/ or Antibodies/ or Immunoglobulin G/ or (immun* or antibod* or "immunoglobulin G" or IgG or opsonophagocyt* or OPA).ti,ab,kf. | 138967 | 146528 |
| 14 | **12 and 13** | 1898 | 1982 |
| 15 | (exp Animals/ or Disease Models, Animal/ or exp Animal Experimentation/ or exp Plants/ or exp Fungi/) not (exp Humans/ or Human Experimentation/) | 3871 | 3871 |
| 16 | (("in vitro" or animal or animals or canine* or dog or dogs or cat or cats or feline or hamster* or lamb or lambs or mice or ferret* or primate* or macaque* or monkey or monkeys or mouse or murine or swine or pig or pigs or piglet* or porcine or rabbit* or rat or rats or rodent* or sheep* or bovine or cow or cows or horse or horses or poultry or chick or chicken* or turkey* or avian* or vertebrate* or veterinary* or plant* or fung*) not (human* or patient* or people or mankind*)).ti,ab,kf. | 26716 | 28034 |
| 17 | **15 or 16** | 28331 | 29650 |
| 18 | **14 not 17** | 1869 | 1953 |
| 19 | Language: English | 1851 | 1935 |
| | limit 19 to yr="2024 -Current" (for update search only) | | 72 |

### Supplementary Table 7. Included pneumococcal conjugate vaccines and their characteristics

| Vaccine | Licensed (FDA) | Serotypes contained in the vaccine | Carrier protein | Amounts of antigens (components) |
|---|---|---|---|---|
| PCV7 | February 2000 | 4, 6B, 9V, 14, 18C, 19F, 23F | Diphtheria toxoid-derived recombinant Cross-Reactive-Material 197 (CRM197) | Each 0.5mL dose contains 2 µg of pneumococcal purified capsular polysaccharides (CPS) for serotypes (STs) 4, 9V, 14, 18C, 19F, 23F, 4 µg of pneumococcal purified CPS for ST 6B; 20 µg CRM197; adsorbed on aluminium phosphate adjuvant (0.125 mg) |
| PCV13 | February 2010 | 4, 6B, 9V, 14, 18C, 19F, 23F, 1, 3, 5, 6A, 7F, 19A | CRM197 | Each 0.5mL dose contains 2.2 µg of pneumococcal purified CPS for STs 1, 3, 4, 5, 6A, 7F, 9V, 14, 18C, 19A, 19F, and 23F, 4.4 µg of pneumococcal purified CPS for ST 6B; 32 µg CRM197; adsorbed on aluminium phosphate adjuvant (0.565 mg) |
| PCV10-SII* | December 2019□ | 6B, 9V, 14, 19F, 23F, 1, 5, 6A, 7F, 19A | CRM197 | Each 0.5mL dose contains 2 µg of pneumococcal purified CPS for STs 1, 5, 9V, 14, 19A, 19F, 23F, 7F, and 6A, and 4 µg of pneumococcal purified CPS for ST 6B; each ST is individually conjugated to the CRM197 (19 to 48 µg); adsorbed on aluminium phosphate adjuvant (0.125 mg) |
| PCV20 | June 2021 | 4, 6B, 9V, 14, 18C, 19F, 23F, 1, 3, 5, 6A, 7F, 19A, 22F, 33F, 8, 10A, 11A, 12F, 15B | CRM197 | Each 0.5mL dose contains 2.2 µg of pneumococcal purified capsular polysaccharides (CPS) for serotypes (STs) 1, 3, 4, 5, 8, 6A, 7F, 9V, 10A, 11A, 12F, 15B, 14, 18C, 19A, 19F 22F,23F, 33F, 4.4 µg of pneumococcal purified CPS for ST 6B; 65 µg CRM197; adsorbed on aluminium phosphate adjuvant (0.565 mg) |
| PCV15 | July 2021 | 4, 6B, 9V, 14, 18C, 19F, 23F, 1, 3, 5, 6A, 7F, 19A, 22F, 33F | CRM197 | Each 0.5mL dose contains 2 µg of pneumococcal purified CPS for STs 1, 3, 4, 5, 6A, 7F, 9V, 14, 18C, 19A, 19F, 22F, 23F, and 33F, 4 µg of pneumococcal purified CPS for ST 6B; 30 µg CRM197; adsorbed on aluminium phosphate adjuvant (0.125 mg) |

*PCV10-SII (PNEUMOSIL) received the World Health Organization (WHO) prequalification in December 2019.

### Supplementary Table 8. Included assays for the quantitation of pneumococcal IgG antibody and assay-specific protective thresholds

| Assay | Preadsorption | Aligned with WHO protective threshold* | Main analysis | Key features |
|---|---|---|---|---|
| First-generation ELISA[1] | None | - | No | Overestimates antibody levels due to detection of both anti-capsular and C-PS antibodies |
| Second-generation ELISA[2] | Pneumococcal C-PS | Yes, 0.35 µg/mL | Yes | Used in PCV7 pivotal trials<br>Specificity improved vs 1st-gen ELISA, but still suboptimal |
| Third-generation ELISA (WHO reference ELISA)[3-7] | C-PS and 22F PS | 0.35 µg/mL | Yes | WHO gold standard assay |
| GSK 22F-ELISA[8] | C-PS and 22F PS | No, 0.20 µg/mL | No | Used 0.20 µg/mL cut-off (vs WHO's 0.35 µg/mL) |
| Pfizer direct Luminex-based immunoassay (dLIA)[9,10] | C-PS and 22F PS | Partial yes. Serotype-specific: mostly 0.35 µg/mL; 5 = 0.23, 6B = 0.10, 19A = 0.12 | Yes (bridged to WHO ELISA) | - |
| Pneumococcal electrochemiluminescence (Pn ECL) assay[11,12] | C-PS and serotypes 25 and 72 | Yes, 0.35 µg/mL | Yes (bridged to WHO ELISA) | - |
| Fluorescent multiplex immunoassay (FMIA)[13-15] | C-PS and 22F PS | No, 0.35 µg/mL (threshold not formally validated) | No | Good agreement with WHO ELISA ($R^2 > 0.8$); 0.35 µg/mL threshold not formally validated for FMIA<br>Cross-reactivity present |

*The WHO has defined a protective IgG concentration cut-off against IPD of 0.35 µg/mL for all serotypes based on model results from three major clinical trials. The WHO-defined protective threshold of 0.35 µg/mL for all

serotypes was used for second-generation ELISA, third-generation ELISA (WHO ELISA was derived from third-generation ELISA), corresponding to 0.20 µg/mL for GSK's 22F-ELISA. For newly developed assays, a threshold of 0.35 µg/mL was applied for both the Pneumococcal (Pn) electrochemiluminescence (ECL)-based detection assay and FMIA methods. For the Pfizer Luminex-based direct immunoassay (dLIA), most serotypes used the 0.35 µg/mL cut-off, except for serotype 5 (0.23 µg/mL), 6B (0.10 µg/mL), and 19A (0.12 µg/mL), based on bridging studies to the WHO reference ELISA.

### Supplementary Table 9. The original and modified JBI tools for randomized clinical trials (RCTs)

| Domains | Questions in JBI tool | Questions in modified JBI tool |
|---|---|---|
| Bias related to selection and allocation | Q1: Was true randomization used for assignment of participants to treatment groups? | - |
| | Q2: Was allocation to treatment groups concealed? | - |
| | Q3: Were treatment groups similar at the baseline? | Yes |
| Bias related to administration of intervention/exposure | Q4: Were participants blind to treatment assignment? | - |
| | Q5: Were those delivering the treatment blind to treatment assignment? | - |
| | Q6: Were treatment groups treated identically other than the intervention of interest? | Yes |
| Bias related to assessment, detection, and measurement of the outcome | Q7: Were outcome assessors blind to treatment assignment? | - |
| | Q8: Were outcomes measured in the same way for treatment groups? | Yes |
| | Q9: Were outcomes measured in a reliable way? | Yes |
| Bias related to participant retention | Q10: Was follow-up complete and, if not, were differences between groups in terms of their follow-up adequately described and analyzed? | Yes |
| Statistical conclusion validity | Q11: Were participants analyzed in the groups to which they were randomized? | Yes, with modified Q11: Were participants analyzed according to the vaccines they received?”. |
| | Q12: Was appropriate statistical analysis used? | Yes |
| | Q13: Was the trial design appropriate and any deviations from the standard RCT design (individual randomization, parallel groups) accounted for in the conduct and analysis of the trial? | - |

### Supplementary Table 10. The original and modified JBI tools for quasi experimental study

| Domains | Questions in JBI tool | Questions in modified JBI tool |
|---|---|---|
| Bias related to temporal precedence | Q1: Is it clear in the study what is the “cause” and what is the “effect” (ie, there is no confusion about which variable comes first)? | - |
| Bias related to selection and allocation | Q2: Was there a control group? | - |
| Bias related to confounding factors | Q3: Were participants included in any comparisons similar? | Yes |
| Bias related to administration of intervention/exposure | Q4: Were the participants included in any comparisons receiving similar treatment/care, other than the exposure or intervention of interest? | Yes |
| Bias related to assessment, detection, and measurement of the outcome | Q5: Were there multiple measurements of the outcome, both pre and post the intervention/exposure? | - |
| | Q6: Were the outcomes of participants included in any comparisons measured in the same way? | Yes |
| | Q7: Were outcomes measured in a reliable way? | Yes |
| Bias related to participant retention | Q8: Was follow-up complete and, if not, were differences between groups in terms of their follow-up adequately described and analyzed? | Yes |
| Statistical conclusion validity | Q9: Was appropriate statistical analysis used? | Yes |

### Supplementary Table 11. The original and modified JBI tools for cohort study

| Domains | Questions in JBI tool | Questions in modified JBI tool |
|---|---|---|
| Bias related to selection and allocation | Q1: Were the two groups similar and recruited from the same population? | Yes |
| Bias related to classification of the exposure | Q2: Were the exposures measured similarly to assign people to both the exposed and unexposed groups? | Yes |

| Domains | Questions in JBI tool | Questions in modified JBI tool |
|---|---|---|
| | Q3: Were the exposures measured similarly to assign people to both the exposed and unexposed groups? | - |
| Bias related to confounding factors | Q4: Were confounding factors identified? | - |
| | Q5: Were strategies to deal with confounding factors stated? | - |
| Bias related to temporal precedence | Q6: Were the groups/participants free of the outcome at the start of the study (or at the moment of the exposure)? | - |
| Bias related to assessment, detection and measurement of the outcome | Q7: Were the outcomes measured in a valid and reliable way? | Yes |
| | Q8: Was the follow-up time reported and sufficient to be long enough for outcomes to occur? | Yes |
| Bias related to participant retention | Q9: Was follow up complete, and if not, were the reasons to loss to follow-up described and explored? | Yes |
| | Q10: Were strategies to address incomplete follow-up utilized? | - |
| Statistical conclusion validity | Q11: Was appropriate statistical analysis used? | Yes |

## Supplementary Table 12. Summary of studies included in immunogenicity analysis

| ID | Author & Year | Study design | Vaccine type | Study arm | Setting | | | Outcome reported | | | |
|---|---|---|---|---|---|---|---|---|---|---|---|
| | | | | | Population | Country/region | Schedule | Type† | Time points* | Assay | Serotype |
| Randomized controlled trials (RCTs) | | | | | | | | | | | |
| 1 | Rennels, 1998[16] | Phase III RCT | PCV7 | 1 | Infants | United States | 3+1 (2-4-6-12 months) | GMC | post dose 2, post dose 3, post-booster | 2nd-gen ELISA | 4, 6B, 9V, 14, 18C, 19F, 23F |
| 2 | Shinefield, 1999[17] | Phase III RCT | PCV7 | 1 | Infants | United States | 3+1 (2-4-6-12 months) | GMC | post dose 3, post-booster | 2nd-gen ELISA | 4, 6B, 9V, 14, 18C, 19F, 23F |
| 3 | Black, 2000[18], US FDA Package Insert: Prevnar for Prevnar Study D118-P8[19] | Phase III RCT | PCV7 | 1 | Infants | United States | 3+1 (2-4-6-12 months) | GMC | Post dose 3, post-booster | 2nd-gen ELISA | 4, 6B, 9V, 14, 18C, 19F, 23F |
| 4 | Eskola, 2001[20], Ekström, 2005[21], Ekström, 2007[22] | Phase III RCT | PCV7 | 1 | Infants | Finland | 3+1 (2-4-6-12 months) | GMC | Post dose 3, post-booster | 1st-gen ELISA | 4, 6B, 9V, 14, 18C, 19F, 23F |
| 5 | Tichmann-Schumann, 2005[23] | Open-labeled RCT | PCV7 | 1 | Infants | Germany | 3+1 (2-3-4-13 months) | GMC | Post dose 3, post-booster | 2nd-gen ELISA | 4, 6B, 9V, 14, 18C, 19F, 23F |
| 6 | Scheifele, 2006[24], Scheifele, 2007[25] | Phase IV RCT | PCV7 | 3 | Infants | Canada | 3+1 (2-4-6-15 or 2-4-6-18 or 3-5-7-15 months) | GMC | post dose 3, post-booster | ELISA without details, conducted in Wyeth Lab using published ELISA | 4, 6B, 9V, 14, 18C, 19F, 23F |
| 7 | Knuf, 2006[26] | Open-labeled RCT | PCV7 | 1 | Infants | Germany | 3+1 (2-3-4-12~15 months) | GMC | post dose 3, post-booster | 2nd-gen ELISA | 4, 6B, 9V, 14, 18C, 19F, 23F |
| 8 | Pichichero, 2007[27] | Phase III RCT | PCV7 | 3 | Infants | United States | 3+0 (2-4-6 or 2.5-4.5-6.5 months) | GMC | Post dose 3 | 3rd-gen ELISA (WHO reference ELISA) | 4, 6B, 9V, 14, 18C, 19F, 23F |
| 9 | O'Brien, 2007[28], Millar, 2007[29] | Phase III RCT | PCV7 | 1 | Navajo and White Mountain Apache children | United States | 3+1 (2-4-6-12 months) | GMC | post dose 1, post dose 2, post dose 3, post-booster | 2nd-gen ELISA | 4, 6B, 9V, 14, 18C, 19F, 23F |
| 10 | Li, 2008[30], NCT00488826[31], Li, 2016[32] | Phase III RCT | PCV7 | 2 | Infants | China | 3+1 (3-4-5-12~15 months) | Both | post dose 3, post-booster | 3rd-gen ELISA (WHO reference ELISA) | 4, 6B, 9V, 14, 18C, 19F, 23F |
| 11 | Olivier, 2008[33] | Phase III RCT | PCV7 | 1 | Infants | France, Germany | 3+1 (2-3-4-12~15 months) | GMC | post dose 3, post-booster | 3rd-gen ELISA (WHO reference ELISA) | 4, 6B, 9V, 14, 18C, 19F, 23F |
| 12 | Dennehy, 2008[34] | Phase III RCT | PCV7 | 2 | Infants | United States | 3+1 (2-4-6-12~15 months) | GMC | Post dose 3 | 3rd-gen ELISA (WHO reference ELISA) | 4, 6B, 9V, 14, 18C, 19F, 23F |
| 13 | Trofa, 2008[35], NCT00197002[36] | Phase III RCT | PCV7 | 2 | Infants | United States | 3+1 (2-4-6-12~15 months) | GMC | Post-booster | GSK 22F-ELISA | 4, 6B, 9V, 14, 18C, 19F, 23F |
| 14 | Vesikari, 2009[37], NCT00370396[38] | Phase III RCT, PCV7 as control | PCV7 | 1 | Infants | Finland, France, Poland | 3+1 (2-3-4-12~18 months) | Both | Post dose 3, post-booster | GSK 22F-ELISA | 4, 6B, 9V, 14, 18C, 19F, 23F |
| 15 | Wysocki, 2009[39] | Phase III RCT, PCV7 as control | PCV7 | 1 | Infants | Germany, Poland, Spain | 3+1 (2-4-6-11~18 months) | Both | Post dose 3, post-booster | GSK 22F-ELISA | 4, 6B, 9V, 14, 18C, 19F, 23F |

| ID | Author & Year | Study design | Vaccine type | Study arm | Setting | | | Outcome reported | | | |
|---|---|---|---|---|---|---|---|---|---|---|---|
| | | | | | Population | Country/region | Schedule | Type† | Time points* | Assay | Serotype |
| 16 | Bermal, 2009[40], Bermal, 2011[41], NCT00344318[42], NCT00547248[43] | Phase III RCT, PCV7 as control | PCV7 | 2 | Infants | Philippines and Poland | 3+1 (6-10-14 weeks and 12~18 months for Philippines or 2-4-6-12~18 months for Poland) | Both | Post dose 3, post-booster | GSK 22F-ELISA | 4, 6B, 9V, 14, 18C, 19F, 23F |
| 17 | Givon-Lavi, 2010[44], Dagan, 2010[45], Dagan, 2012[46], Dagan, 2018[47] | Phase III RCT | PCV7 | 3 | Infants | Israel | 3+1 (2-4-6-12 months) or 3+0 (2-4-6 months) or 2+1 (4-6-12 months) | Both | Post dose 2 (for 2+1 only), post dose 3 (for 3+1 and 3+0), post-booster | 3rd-gen ELISA (WHO reference ELISA) | 4, 6B, 9V, 14, 18C, 19F, 23F |
| 18 | Wysocki, 2010[48] | Phase III RCT | PCV7 | 2 | Infants | Poland | 3+1 (2-3.5-6-12 months) | Both | Post dose 3, post-booster | 3rd-gen ELISA (WHO reference ELISA) | 4, 6B, 9V, 14, 18C, 19F, 23F |
| 19 | Goldblatt, 2010[49] | Phase IV RCT | PCV7 | 3 | Infants | United Kingdom | 2+1 (2-3-12 months) or 2+1 (2-4-12 months) | Both | Post dose 2, post-booster | 3rd-gen ELISA (WHO reference ELISA) | 4, 6B, 9V, 14, 18C, 19F, 23F |
| 20 | Grimprel, 2011[50] | Phase III RCT | PCV7 | 2 | Infants | France, Poland | 3+1 (2-3-4-12~18 months) | Both | Post dose 3, post-booster | 3rd-gen ELISA (WHO reference ELISA) | 4, 6B, 9V, 14, 18C, 19F, 23F |
| 21 | Scott, 2011[51] | Phase III RCT | PCV7 | 2 | Infants | Kenya | 3+1 (1.5-2.5-3.5-9 months) or 3+1 (0-2.5-3.5-9 months) | Both | post dose 1, post dose 2, post dose 3, post-booster | 3rd-gen ELISA (WHO reference ELISA) | 4, 6B, 9V, 14, 18C, 19F, 23F |
| 22 | van den Bergh, 2011[52], van den Bergh, 2016[53], NCT00652951[54] | Phase III RCT, PCV7 as control | PCV7 | 1 | Infants | Netherlands | 3+1 (2-3-4-11~13 months) | Both | Post dose 3, post-booster | GSK 22F-ELISA | 4, 6B, 9V, 14, 18C, 19F, 23F |
| 23 | Kim, 2011[55], NCT00680914[56], NCT00911144[57] | Phase III RCT, PCV7 as control | PCV7 | 1 | Infants | South Korea | 3+1 (2-4-6-12~18 months) | Both | Post dose 3, post-booster | GSK 22F-ELISA | 4, 6B, 9V, 14, 18C, 19F, 23F |
| 24 | Leonardi, 2011[58], NCT00109343[59] | Phase III RCT | PCV7 | 2 | Infants | United States | 3+1 (2-4-6-12~15 months) | Both | Post-booster | 3rd-gen ELISA (Merck Pn ELISA) | 4, 6B, 9V, 14, 18C, 19F, 23F |
| 25 | Marshall, 2011[60] | Phase II RCT | PCV7 | 2 | Infants | United States | 3+1 (2-4-6-12~15 months) | Both | Post dose 3, post booster | GSK 22F-ELISA | 4, 6B, 9V, 14, 18C, 19F, 23F |
| 26 | Blatter, 2012[61], NCT00578175[62] | Phase III RCT | PCV7 | 3 | Infants | United States | 3+1 (2-4-6-12~14 months) | Both | Post-booster | GSK 22F-ELISA | 4, 6B, 9V, 14, 18C, 19F, 23F |
| 27 | Klein, 2012[63], NCT00474526[64] | Phase III RCT | PCV7 | 2 | Infants | United States, Colombia, Argentina | 3+1 (2-4-6-12 months) | Both | Post dose 3 | ELISA without detail | 4, 6B, 9V, 14, 18C, 19F, 23F |
| 28 | Tapiéro, 2013[65], Halperin et al, 2014[66] | Phase II RCT | PCV7 | 2 | Infants | Canada | 3+1 (2-4-6-12 months) | GMC | Post dose 3, post-booster | 3rd-gen ELISA (Merck Pn ELISA) | 4, 6B, 9V, 14, 18C, 19F, 23F |
| 29 | Vesikari, 2013[67], NCT00657709[68] | Phase III RCT | PCV7 | 2 | Infants | Finland, Czech Republic, Germany, Austria, Italy | 3+1 (2-4-6-12 months) | GMC | Post dose 3 | ELISA without detail | 4, 6B, 9V, 14, 18C, 19F, 23F |

| ID | Author & Year | Study design | Vaccine type | Study arm | Setting | | | Outcome reported | | | |
|---|---|---|---|---|---|---|---|---|---|---|---|
| | | | | | Population | Country/region | Schedule | Type† | Time points* | Assay | Serotype |
| 30 | van Westen, 2013[69], Rodenburg, 2010[70] | Phase III RCT | PCV7 | 2 | Infants | Netherlands | 3+1 (2-3-4-11 months) or 2+1 (2-4-11 months) or 2+0 (2-4 months) | Both | Post-booster | 3rd-gen ELISA (WHO reference ELISA) | 4, 6B, 9V, 14, 18C, 19F, 23F |
| 31 | Yetman, 2013[71], NCT00312858[72] | Phase IV RCT | PCV7 | 2 | Infants | United States | 3+1 (2-4-6-12~15 months) | Both | Post-booster | 3rd-gen ELISA (Merck Pn ELISA) | 4, 6B, 9V, 14, 18C, 19F, 23F |
| 32 | Prymula, 2014[73], Esposito, 2014[74] | Phase II RCT | PCV7 | 3 | Infants | Czech Republic, Italy, Hungary, Chile, Argentina | 3+1 (2-3-4-12 months) | Both | Post dose 3 | 3rd-gen ELISA (WHO reference ELISA) | 4, 6B, 9V, 14, 18C, 19F, 23F |
| 33 | López, 2017[75], NCT01444781[76] | Phase III RCT | PCV7 | 3 | Infants | Colombia, Costa Rica | 3+1 (2-4-6-12~24 months) | Both | Post dose 3, post booster | ELISA without detail | 4, 6B, 9V, 14, 18C, 19F, 23F |
| 34 | Zhao, 2022[77] | Phase III RCT, PCV7 as control | PCV7 | 1 | Infants | China | 3+1 (3-4-5-12~15 months) | Both | Post dose 3, post-booster | 3rd-gen ELISA (WHO reference ELISA) | 4, 6B, 9V, 14, 18C, 19F, 23F |
| 35 | FDA Package Insert: Prevnar for Study D118-P16[19] | Phase II RCT | PCV7 | 1 | Infants | United States | 3+1 (2-4-6-12~15 months) | GMC | Post dose 3 | 2nd-gen ELISA | 4, 6B, 9V, 14, 18C, 19F, 23F |
| 36 | EUCTR2007-004276-39[78] | Phase III RCT | PCV7 | 2 | Infants | Germany | 3+1 (2-3-4-13 months) | Both | Post booster | ELISA without detail | 4, 6B, 9V, 14, 18C, 19F, 23F |
| 37 | NCT01250756[79] | Phase IV RCT | PCV7 (subcuta-neously) | 1 | Infants | Japan | 3+1 (3-4-5-12~15 months) | Both | Post dose 3, post booster | ELISA without detail | 4, 6B, 9V, 14, 18C, 19F, 23F |
| 38 | Thisyakorn, 2014[80] | Phase III RCT | PCV7 | 1 | Infants | Thailand | 3+1 (2-4-6-12~18 months) | GMC | Post-booster | 3rd-gen ELISA (WHO reference ELISA) | 4, 6B, 9V, 14, 18C, 19F, 23F |
| 39 | Grimprel, 2011[81], NCT00366678[82], NCT01026038[83] | Phase III RCT | PCV7 & PCV13 | 2 | Infants | France | 3+1 (2-3-4-12 months) | Both | Post dose 3, post-booster | 3rd-gen ELISA (WHO reference ELISA) | 4, 6B, 9V, 14, 18C, 19F, 23F, 1, 3, 5, 6A, 7F, 19A |
| 40 | Sobanjo-ter Meulen, 2015[84], EUCTR2009-015103-58-FI[85], NCT01215175[86] | Phase I RCT for PCV15 (PCV7 primary series with PCV15 booster), PCV7 as control | PCV7 | 1 | Infants | United States, Finland | 3+1 (2-4-6-12~15 months) | Both | Post-booster | Pn ECL | 4, 6B, 9V, 14, 18C, 19F, 23F |
| 41 | Kieninger , 2010[87], NCT00366340[88], EUCTR2005-004770-24[89] | Phase III RCT | PCV7 & PCV13 | 2 | Infants | Germany | 3+1 (2-3-4-11~12 months) | Both | Post dose 3, post-booster | 3rd-gen ELISA (WHO reference ELISA) | 4, 6B, 9V, 14, 18C, 19F, 23F, 1, 3, 5, 6A, 7F, 19A |
| 42 | Esposito, 2010[90], NCT00366899[91], EUCTR2005-004771-38-IT[92], Rodgers, 2013[93] | Phase III RCT | PCV7 & PCV13 | 2 | Infants | Italy | 2+1 (3-5-11 months) | Both | Post dose 2, post-booster | 3rd-gen ELISA (WHO reference ELISA) | 4, 6B, 9V, 14, 18C, 19F, 23F, 1, 3, 5, 6A, 7F, 19A |

| ID | Author & Year | Study design | Vaccine type | Study arm | Setting | | | Outcome reported | | | |
|---|---|---|---|---|---|---|---|---|---|---|---|
| | | | | | Population | Country/region | Schedule | Type† | Time points* | Assay | Serotype |
| 43 | Snape, 2010[94]; Rodgers, 2013[93], NCT00384059[95], EUCTR2005-005130-12[96] | Phase III RCT | PCV7 & PCV13 | 2 | Infants | United Kingdom | 2+1 (2-4-12 months) | Both | Post dose 2, post-booster | 3rd-gen ELISA (WHO reference ELISA) | 4, 6B, 9V, 14, 18C, 19F, 23F, 1, 3, 5, 6A, 7F, 19A |
| 44 | Bryant, 2010[97], NCT00205803[98] | Phase I and II RCT | PCV7 & PCV13 | 2 | Infants | United States | 3+1 (2-4-6-12 months) | Both | Post dose 3, post-booster | 3rd-gen ELISA (WHO reference ELISA) | 4, 6B, 9V, 14, 18C, 19F, 23F, 1, 3, 5, 6A, 7F, 19A |
| 45 | Yeh, 2010[99], NCT00373958[100] | Phase III RCT | PCV7 & PCV13 | 2 | Infants | United States | 3+1 (2-4-6-12 months) | Both | Post dose 3, post booster | 3rd-gen ELISA (WHO reference ELISA) | 4, 6B, 9V, 14, 18C, 19F, 23F, 1, 3, 5, 6A, 7F, 19A |
| 46 | Weckx, 2012[101], NCT00676091[102] | Phase III RCT | PCV7 & PCV13 | 2 | Infants | Brazil | 3+1 (2-4-6-12 months) | Both | Post dose 3, post-booster | 3rd-gen ELISA (WHO reference ELISA) | 4, 6B, 9V, 14, 18C, 19F, 23F, 1, 3, 5, 6A, 7F, 19A |
| 47 | Huang, 2012[103], NCT00688870[104] | Phase III RCT | PCV7 & PCV13 | 2 | Infants | Taiwan | 3+1 (2-4-6-15 months) | Both | Post dose 3, post-booster | 3rd-gen ELISA (WHO reference ELISA) | 4, 6B, 9V, 14, 18C, 19F, 23F, 1, 3, 5, 6A, 7F, 19A |
| 48 | Amdekar, 2013[105] | Phase III RCT | PCV7 & PCV13 | 2 | Infants | India | 3+1 (1.5-2.5-3.5-12 months) | Both | Post dose 3, post-booster | 3rd-gen ELISA (WHO reference ELISA) | 4, 6B, 9V, 14, 18C, 19F, 23F, 1, 3, 5, 6A, 7F, 19A |
| 49 | Dagan, 2013[106], Juergens, 2014[107], Dagan, 2021[108] | Phase III RCT | PCV7 & PCV13 | 2 | Infants | Israel | 3+1 (2-4-6-12 months) | GMC | Post dose 3, post booster | 3rd-gen ELISA (WHO reference ELISA) | 4, 6B, 9V, 14, 18C, 19F, 23F, 1, 3, 5, 6A, 7F, 19A |
| 50 | Kim, 2013[109], NCT00689351[110] | Phase II RCT | PCV7 & PCV13 | 2 | Infants | South Korea | 3+1 (2-4-6-12 months) | Both | Post dose 3, post booster | 3rd-gen ELISA (WHO reference ELISA) | 4, 6B, 9V, 14, 18C, 19F, 23F, 1, 3, 5, 6A, 7F, 19A |
| 51 | Rodgers, 2013[93]; Diez-Domingo, 2013[111], NCT00368966[112] | Phase III RCT | PCV7 & PCV13 | 2 | Infants | Spain | 3+1 (2-4-6-15 months) | Both | Post dose 2, post dose 3, post-booster | 3rd-gen ELISA (WHO reference ELISA) | 4, 6B, 9V, 14, 18C, 19F, 23F, 1, 3, 5, 6A, 7F, 19A |
| 52 | Payton, 2013[113], NCT00444457[114] | Phase III RCT | PCV7 & PCV13 | 4 | Infants | United States | 3+1 (2-4-6-12 months) | Both | Post dose 3, post booster | 3rd-gen ELISA (WHO reference ELISA) | 4, 6B, 9V, 14, 18C, 19F, 23F, 1, 3, 5, 6A, 7F, 19A |
| 53 | Togashi, 2015[115], NCT01200368[116] | Phase III RCT | PCV7 & PCV13 (subcuta-neously) | 2 | Infants | Japan | 3+1 (3-4-5-12~15 months) | Both | Post dose 3, post-booster | 3rd-gen ELISA (WHO reference ELISA) | 4, 6B, 9V, 14, 18C, 19F, 23F, 1, 3, 5, 6A, 7F, 19A |

| ID | Author & Year | Study design | Vaccine type | Study arm | Setting | | | Outcome reported | | | |
|---|---|---|---|---|---|---|---|---|---|---|---|
| | | | | | Population | Country/region | Schedule | Type† | Time points* | Assay | Serotype |
| 54 | Zhu, 2016[117], Zhu, 2019[118] | Phase III RCT | PCV7 & PCV13 | 4 | Infants | China | 3+1 (2-4-6-12 months or 3-4-5-12 months) or 2+1 (3-5-12 months), PCV7 had 3+1 (3-4-5-12 months) only | Both | Post dose 3, post booster | 3rd-gen ELISA (WHO reference ELISA) | 4, 6B, 9V, 14, 18C, 19F, 23F, 1, 3, 5, 6A, 7F, 19A |
| 55 | NCT00452790[119] | Phase III RCT | PCV7 & PCV13 | 2 | Infants | India | 3+1 (1.5-2.5-3.5-12 months) | Both | Post dose 3, post booster | 3rd-gen ELISA (WHO reference ELISA) | 4, 6B, 9V, 14, 18C, 19F, 23F, 1, 3, 5, 6A, 7F, 19A |
| 56 | Gadzinowski, 2011[120], NCT00464945[121], EUCTR2006-006204-11[122] | Phase III RCT | PCV13 | 2 | Infants | Poland | 3+1 (2-3-4-12 months) | Both | Post dose 3 | 3rd-gen ELISA (WHO reference ELISA) | 4, 6B, 9V, 14, 18C, 19F, 23F, 1, 3, 5, 6A, 7F, 19A |
| 57 | Vanderkooi, 2012[123], NCT00475033[124] | Phase III RCT | PCV13 | 1 | Infants | Canada | 3+1 (2-4-6-12 months) | Both | Post dose 3, post-booster | 3rd-gen ELISA (WHO reference ELISA) | 4, 6B, 9V, 14, 18C, 19F, 23F, 1, 3, 5, 6A, 7F, 19A |
| 58 | Spijkerman, 2013[125], van Westen, 2018[126] | Phase IV RCT | PCV13 | 4 | Infants | Netherlands | 3+1 (2-4-6-11.5 months or 2-3-4-11.5 months) or 2+1 (3-5-11.5 months or 2-4-11.5 months) | Both | Post dose 2, post dose 3, post-booster | FMIA | 4, 6B, 9V, 14, 18C, 19F, 23F, 1, 3, 5, 6A, 7F, 19A |
| 59 | Rodgers, 2013[93], Martinón-Torres, 2012[127], NCT00474539[128], EUCTR2007-000304-32[129] | Phase III RCT | PCV13 | 1 | Infants | Spain | 3+1 (2-4-6-15 months) | Both | Post dose 2, post dose 3, post-booster | 3rd-gen ELISA (WHO reference ELISA) | 4, 6B, 9V, 14, 18C, 19F, 23F, 1, 3, 5, 6A, 7F, 19A |
| 60 | Gadzinowski, 2015[130], NCT00366548[131] | Phase III RCT | PCV13 | 1 | Infants | Poland | 3+1 (2-3-4-12 months) | Both | Post dose 3, post-booster | 3rd-gen ELISA (WHO reference ELISA) | 4, 6B, 9V, 14, 18C, 19F, 23F, 1, 3, 5, 6A, 7F, 19A |
| 61 | Iro, 2015[132] | Phase IV RCT | PCV13 | 2 | Infants | United Kingdom, Malta | 2+1 (2-4-12 months) | Both | Post dose 2, post-booster | FMIA | 4, 6B, 9V, 14, 18C, 19F, 23F, 1, 3, 5, 6A, 7F, 19A |
| 62 | Truck, 2016[133] | Phase III RCT | PCV13 | 1 | Infants | United Kingdom | 2+1 (2-4-12 months) | Both | Post-booster | 3rd-gen ELISA (WHO reference ELISA) | 4, 6B, 9V, 14, 18C, 19F, 23F, 1, 3, 5, 6A, 7F, 19A |
| 63 | Block, 2016[134] | Phase III RCT | PCV13 | 3 | Infants | United States, Canada | 3+1 (2-4-6-12 months) | Both | Post dose 3, post-booster | 3rd-gen ELISA (WHO reference ELISA) | 4, 6B, 9V, 14, 18C, 19F, 23F, |

| ID | Author & Year | Study design | Vaccine type | Study arm | Setting | | | Outcome reported | | | |
|---|---|---|---|---|---|---|---|---|---|---|---|
| | | | | | Population | Country/region | Schedule | Type† | Time points* | Assay | Serotype |
| | | | | | | | | | | | 1, 3, 5, 6A, 7F, 19A |
| 64 | Prymula, 2017[135], NCT01204658[136] | Phase II RCT, PCV13 as control | PCV13 | 1 | Infants | Czech Republic, Germany, Poland, Sweden | 3+1 (2-3-4-12~15 months) | Both | Post dose 3, post-booster | GSK 22F-ELISA | 4, 6B, 9V, 14, 18C, 19F, 23F, 1, 3, 5, 6A, 7F, 19A |
| 65 | Vesikari, 2017[137], NCT01248884[138], NCT01453998[138] | Phase III RCT | PCV13 | 3 | Infants | Dominican Republic, Finland | 3+1 (2-3-4-12~15 months) | Both | Post dose 3, post-booster | GSK 22F-ELISA | 4, 6B, 9V, 14, 18C, 19F, 23F, 1, 3, 5, 6A, 7F, 19A |
| 66 | Idoko, 2017[139], NCT01964716[140], EUCTR2012-000482-21[141] | Phase III RCT | PCV13 & PCV13 (multi-dose vials) | 2 | Infants | Gambia | 3+0 (2-3-4 months) | Both | Post dose 3 | 3rd-gen ELISA (WHO reference ELISA) | 4, 6B, 9V, 14, 18C, 19F, 23F, 1, 3, 5, 6A, 7F, 19A |
| 67 | Wysocki, 2017[142], NCT01392378[143], EUCTR2010-022303-22[144] | Phase IV RCT | PCV13 | 1 | Infants | Poland | 3+1 (2-3-4-12 months) | Both | Post dose 3, post booster | 3rd-gen ELISA (WHO reference ELISA) | 4, 6B, 9V, 14, 18C, 19F, 23F, 1, 3, 5, 6A, 7F, 19A |
| 68 | Cutland, 2018[145], NCT01939158[146] | Phase III RCT | PCV13 | 2 | Infants | Australia, Canada, Czech Republic, Panama, South Africa, Turkey | 3+1 (2-4-6-12~14 months) | Both | Post-booster | 3rd-gen ELISA (WHO reference ELISA) | 4, 6B, 9V, 14, 18C, 19F, 23F, 1, 3, 5, 6A, 7F, 19A |
| 69 | Prymula, 2018[147], EUCTR2012-001055-39[148], EUCTR2012-001042-18-ES[149] | Phase III RCT | PCV13 | 2 | Infants | Germany, Czech Republic | 3+1 (2-3-4-11~15 months) | Both | Post dose 3, post-booster | 3rd-gen ELISA (WHO reference ELISA) | 4, 6B, 9V, 14, 18C, 19F, 23F, 1, 3, 5, 6A, 7F, 19A |
| 70 | Goldblatt, 2018[150], EUCTR2015-000817-32[151] | Phase II RCT | PCV13 | 2 | Infants | United Kingdom | 2+1 (2-4-12 months) or 1+1 (3-12 months) | Both | Post dose 2, post-booster | 3rd-gen ELISA (WHO reference ELISA) | 4, 6B, 9V, 14, 18C, 19F, 23F, 1, 3, 5, 6A, 7F, 19A |
| 71 | Temple, 2019[152] | Phase II and III RCT | PCV13 | 1 | Infants | Vietnam | 2+1 (2-4-9.5 months) | Both | Post dose 1, post dose 2, post-booster | 3rd-gen ELISA (WHO reference ELISA) | 4, 6B, 9V, 14, 18C, 19F, 23F, 1, 3, 5, 6A, 7F, 19A |
| 72 | Moïsi, 2019[153] | Phase IV RCT | PCV13 | 2 | Infants | Burkina Faso | 2+1 (1.5-3.5-9 months) or 3+0 (1.5-2.5-3.5 months) | Both | Post dose 2, post-booster or post dose 3 | 3rd-gen ELISA (WHO reference ELISA) | 4, 6B, 9V, 14, 18C, 19F, 23F, 1, 3, 5, 6A, 7F, 19A |
| 73 | Carmona Martinez, 2019[154], NCT01616459[155] | Phase II RCT, PCV13 as control | PCV13 | 1 | Infants | Czech Republic, Germany, Poland, Spain | 3+1 (2-3-4-12~15 months) | Both | Post dose 3, post-booster | GSK 22F-ELISA | 4, 6B, 9V, 14, 18C, 19F, 23F, 1, 3, 5, 6A, 7F, 19A |
| 74 | Odutola, 2019[156], NCT01262872[157] | Phase II RCT, PCV13 as control | PCV13 | 1 | Infants | Gambia | 3+0 (2-3-4 months) | Both | Post dose 3 | GSK 22F-ELISA | 4, 6B, 9V, 14, 18C, 19F, 23F, 1, 3, 5, 6A, 7F, 19A |

| ID | Author & Year | Study design | Vaccine type | Study arm | Setting | | | Outcome reported | | | |
|---|---|---|---|---|---|---|---|---|---|---|---|
| | | | | | Population | Country/region | Schedule | Type† | Time points* | Assay | Serotype |
| 75 | Klein, 2019[158], NCT01978093[159] | Phase III RCT, co-administered with Hib-MenCY-TT | PCV13 | 2 | Infants | United States | 3+1 (2-4-6-12~15 months) | Both | Post dose 3, post-booster | 3rd-gen ELISA (WHO reference ELISA) | 4, 6B, 9V, 14, 18C, 19F, 23F, 1, 3, 5, 6A, 7F, 19A |
| 76 | Madhi, 2020[160], Mutsaerts, 2024[161] | Phase III RCT | PCV13 | 3 | Infants | South Africa | 2+1 (1.5-3.5-9 months) or 1+1 (3.5-9 months or 1.5-9 months) | Both | Post dose 1, post dose 2, post-booster | 3rd-gen ELISA (WHO reference ELISA with 007sp) | 4, 6B, 9V, 14, 18C, 19F, 23F, 1, 3, 5, 6A, 7F, 19A |
| 77 | Shin, 2020[162] | Phase II RCT, PCV13 as control | PCV13 | 1 | Infants | Thailand | 2+1 (2-4-10~13 months) | Both | Post dose 2, post-booster | 3rd-gen ELISA (WHO reference ELISA) | 4, 6B, 9V, 14, 18C, 19F, 23F, 1, 3, 5, 6A, 7F, 19A |
| 78 | Leach, 2021[163], Leach, 2022[164] | Phase IV RCT | PCV13 | 1 | Australian Aboriginal infants | Australia | 3+0 (2-4-6 months) | Both | Post dose 3 | 3rd-gen ELISA (WHO reference ELISA) | 4, 6B, 9V, 14, 18C, 19F, 23F, 1, 3, 5, 6A, 7F, 19A |
| 79 | Lalwani, 2021[165], NCT03548337[166] | Phase IV RCT | PCV13, PCV13 (multi-dose vials) | 2 | Infants | India | 3+1 (6-10-14 weeks-12 months) | Both | Post dose 3, post-booster | Pfizer dLIA | 4, 6B, 9V, 14, 18C, 19F, 23F, 1, 3, 5, 6A, 7F, 19A |
| 80 | Dhingra, 2021[167], NCT03205371[168] | Phase III RCT, co-administered with Hib-MenCY-TT | PCV13 | 2 | Infants | the Russian Federation | 2+1 (2-4-15~23 months) | Both | Post-booster | Pn ECL | 4, 6B, 9V, 14, 18C, 19F, 23F, 1, 3, 5, 6A, 7F, 19A |
| 81 | Wang, 2022[169] | Phase III RCT | PCV13 | 1 | Infants | China | 3+1 (2-4-6-13 months) | Both | Post dose 3, post-booster | 3rd-gen ELISA (WHO reference ELISA) | 4, 6B, 9V, 14, 18C, 19F, 23F, 1, 3, 5, 6A, 7F, 19A |
| 82 | Kawade, 2023[170] | Phase IV RCT | PCV13 | 3 | Infants | India | 1+1 (3.5-9 months) or 2+1 (1.5-3.5-9 months) or 3+0 (1.5-2.5-3.5 months) | Both | Post dose 1, post dose 2, post dose 3, post-booster | 3rd-gen ELISA (WHO reference ELISA) | 4, 6B, 9V, 14, 18C, 19F, 23F, 1, 3, 5, 6A, 7F, 19A |
| 83 | Sanchez, 2023[171] | Phase III RCT | PCV13 | 2 | Infants | Thailand | 3+1 (2-4-6-15~18 months) | Both | Post dose 3 | Pn ECL | 4, 6B, 9V, 14, 18C, 19F, 23F, 1, 3, 5, 6A, 7F, 19A |
| 84 | Rajan, 2023[172] | Phase III RCT | PCV13 | 2 | Infants | United Kingdom | 1+1 (3-12 months) | Both | Post dose 1, post booster | 3rd-gen ELISA (WHO reference ELISA) | 4, 6B, 9V, 14, 18C, 19F, 23F, 1, 3, 5, 6A, 7F, 19A |
| 85 | Simon, 2023[173], NCT03550313[174] | Phase II RCT of a complementary 7-valent PCV, PCV13 as control | PCV13 | 1 | Infants | United States | 3+1 (2-4-6-12 months) | Both | Post dose 3, post-booster | Pfizer dLIA | 4, 6B, 9V, 14, 18C, 19F, 23F, 1, 3, 5, 6A, 7F, 19A |

| ID | Author & Year | Study design | Vaccine type | Study arm | Setting | | | Outcome reported | | | |
|---|---|---|---|---|---|---|---|---|---|---|---|
| | | | | | Population | Country/region | Schedule | Type† | Time points* | Assay | Serotype |
| 86 | Temple, 2023[175] | Phase II and III RCT | PCV13 | 2 | Infants | Vietnam | 0+1 (12 months) or 1+1 (2-12 months) | Both | Post dose 1, post-booster | 3rd-gen ELISA (WHO reference ELISA) | 4, 6B, 9V, 14, 18C, 19F, 23F, 1, 3, 5, 6A, 7F, 19A |
| 87 | Xie, 2024[176] | Phase III RCT, PCV13 as control | PCV13 | 1 | Infants | China | 3+1 (2-4-6-12~15 months) | Both | Post dose 3, post-booster | 3rd-gen ELISA (WHO reference ELISA) | 4, 6B, 9V, 14, 18C, 19F, 23F, 1, 3, 5, 6A, 7F, 19A |
| 88 | Matur, 2024[177] | Phase III RCT, PCV13 as control | PCV13 | 1 | Infants | India | 3+0 (1.5-2.5-3.5 months) | Rate | Post dose 3 | 2nd-gen ELISA (WHO reference ELISA with a minor modification) | 4, 6B, 9V, 14, 18C, 19F, 23F, 1, 3, 5, 7F, 19A (without 6A) |
| 89 | Gallagher, 2024[178] | Phase IV RCT | PCV13 | 1 | Infants | Kenya | 2+1 (1.5-3.5-9~12 months) | Rate | Post dose 2 | 3rd-gen ELISA (WHO reference ELISA) | 4, 6B, 9V, 14, 18C, 19F, 23F, 1, 3, 5, 6A, 7F, 19A |
| 90 | Borys, 2024[179] | Phase I RCT, PCV13 as control | PCV13 | 1 | Infants | United States | 3+1 (2-4-6-12 months) | Rate | Post-booster | Pfizer dLIA | 4, 6B, 9V, 14, 18C, 19F, 23F, 1, 3, 5, 6A, 7F, 19A |
| 91 | NCT01090453[180] | Phase II RCT, co-administered with DTPa-IPV/Hib-MenC-TT | PCV13 | 2 | Infants | Canada, France, Germany | 2+1 (2-4-12 months) | Both | Post dose 2, post booster | GSK 22F-ELISA | 4, 6B, 9V, 14, 18C, 19F, 23F, 1, 3, 5, 6A, 7F, 19A |
| 92 | NCT03207750[181] | Phase III RCT, co-administered with human rotavirus vaccine | PCV13 | 2 | Infants | United States | 3+0 (2-4-6 months) | GMC | Post dose 3 | 3rd-gen ELISA (WHO reference ELISA) | 4, 6B, 9V, 14, 18C, 19F, 23F, 1, 3, 5, 6A, 7F, 19A |
| 93 | Bili, 2023[182], NCT03620162[183], EUCTR2018-001151-12[184] | Phase III RCT, PCV13 as control | PCV13 & PCV15 | 2 | Infants | USA, Puerto Rico, Thailand, Turkey | 3+1 (2-4-6-12~15 months) | Both | Post dose 3, post booster | Pn ECL | 4, 6B, 9V, 14, 18C, 19F, 23F, 1, 3, 5, 6A, 7F, 19A, 22F, 33F |
| 94 | NCT05408429[185], EUCTR2021-006624-41[186] | Phase III RCT, PCV13 as control | PCV13 | 1 | Infants | Hungary, Poland, Spain | 2+1 (2-4-12 months) | Both | Post booster | Pfizer dLIA | 4, 6B, 9V, 14, 18C, 19F, 23F, 1, 3, 5, 6A, 7F, 19A |
| 95 | Greenberg, 2018[187], NCT01215188[188], EUCTR2010-019775-29[189] | Phase II RCT, PCV13 as control | PCV13 | 1 | Infants | United States, Canada, Finland, Israel, and Spain | 3+1 (2-4-6-12 months) | GMC | Post dose 3, post-booster | Pn ECL | 4, 6B, 9V, 14, 18C, 19F, 23F, 1, 3, 5, 6A, 7F, 19A, 22F, 33F |
| 96 | Rupp, 2019[190], NCT02531373[191] | Phase I and II RCT, PCV13 as control | PCV13 & PCV15 | 3 | Infants | United States | 3+1 (2-4-6-12~15 months) | Both | Post dose 3, post booster | Pn ECL | 4, 6B, 9V, 14, 18C, 19F, 23F, 1, 3, 5, 6A, 7F, 19A, 22F, 33F |

| ID | Author & Year | Study design | Vaccine type | Study arm | Setting | | | Outcome reported | | | |
|---|---|---|---|---|---|---|---|---|---|---|---|
| | | | | | Population | Country/region | Schedule | Type† | Time points* | Assay | Serotype |
| 97 | Platt, 2020[192], NCT02987972[193] | Phase II RCT, PCV13 as control | PCV13 & PCV15 | 3 | Infants | Finland, Spain, Israel, Denmark, Canada, the United States | 3+1 (2-4-6-12~15 months) | Both | Post dose 3, post booster | Pn ECL | 4, 6B, 9V, 14, 18C, 19F, 23F, 1, 3, 5, 6A, 7F, 19A, 22F, 33F |
| 98 | Banniettis, 2022[194], EUCTR2018-003706-88[195], NCT03885934[196] | Phase III RCT, PCV13 as control | PCV13 & PCV15 | 2 | Infants | Finland, Malaysia, Poland, the Russian Federation, Thailand | 2+1 (9-10-12 months) | GMC | Post booster | Pn ECL | 4, 6B, 9V, 14, 18C, 19F, 23F, 1, 3, 5, 6A, 7F, 19A, 22F, 33F |
| 99 | Martinón-Torres, 2023[197], NCT04031846[198] | Phase III RCT, PCV13 as control | PCV13 & PCV15 | 2 | Infants | Australia, Belgium, Czech Republic, Estonia, Germany, Greece, Poland, Russian Federation, Spain | 2+1 (2-4-11~15 months) | Both | Post dose 2, post booster | Pn ECL | 4, 6B, 9V, 14, 18C, 19F, 23F, 1, 3, 5, 6A, 7F, 19A, 22F, 33F |
| 100 | Benfield, 2023[199], NCT04016714[200] | Phase III RCT, PCV13 as control | PCV13 & PCV15 | 2 | Infants | Denmark, Finland, Italy, Norway | 2+1 (3-5-12 months) | Both | Post dose 2, post-booster | Pn ECL | 4, 6B, 9V, 14, 18C, 19F, 23F, 1, 3, 5, 6A, 7F, 19A, 22F, 33F |
| 101 | Suzuki, 2023[201], NCT04384107[202] | Phase III RCT, PCV13 as control | PCV13 & PCV15 (subcuta-neously) | 2 | Infants | Japan | 3+1 (2-3-4-12~15 months) | Both | Post dose 3, post-booster | Pn ECL | 4, 6B, 9V, 14, 18C, 19F, 23F, 1, 3, 5, 6A, 7F, 19A, 22F, 33F |
| 102 | Ishihara, 2023[203], Wan, 2024[204], NCT03848065[205] | Phase I RCT, PCV13 as control | PCV13 (subcuta-neously) & PCV15 (both subcuta-neously and intramu-scularly) | 3 | Infants | Japan | 3+1 (3-4-5-12~15 months) | Both | Post dose 3, post-booster | Pn ECL | 4, 6B, 9V, 14, 18C, 19F, 23F, 1, 3, 5, 6A, 7F, 19A, 22F, 33F |
| 103 | Lupinacci, 2023[206], NCT03893448[207] | Phase III RCT, PCV13 as control | PCV13 & PCV15 | 2 | Infants | United States, Puerto Rico, Thailand, Turkey | 3+1 (2-4-6-15~18 months) | Both | Post dose 3, post booster | Pn ECL | 4, 6B, 9V, 14, 18C, 19F, 23F, 1, 3, 5, 6A, 7F, 19A, 22F, 33F |
| 104 | Senders, 2021[208], NCT03512288[209] | Phase II RCT, PCV13 as control | PCV13 & PCV20 | 2 | Infants | United States | 3+1 (2-4-6-12 months) | GMC | Post dose 3, post-booster | Pfizer dLIA | 4, 6B, 9V, 14, 18C, 19F, 23F, 1, 3, 5, 6A, 7F, 19A, 22F, 33F, 8, 10A, 11A, 12F,15B |
| 105 | Korbal, 2024[210], NCT04546425[211] | Phase III RCT, PCV13 as control | PCV13 & PCV20 | 2 | Infants | Australia, Czechia, Denmark, Estonia, Finland, Italy, Netherlands, Norway, Poland, Russian Federation, Slovakia | 2+1 (2-4-12 months) | Both | Post dose 2, post-booster | Pfizer dLIA | 4, 6B, 9V, 14, 18C, 19F, 23F, 1, 3, 5, 6A, 7F, 19A, 22F, 33F, 8, 10A, 11A, 12F,15B |

| ID | Author & Year | Study design | Vaccine type | Study arm | Setting | | | Outcome reported | | | |
|---|---|---|---|---|---|---|---|---|---|---|---|
| | | | | | Population | Country/region | Schedule | Type† | Time points* | Assay | Serotype |
| 106 | Ishihara, 2024[212], NCT04530838[213] | Phase III RCT, PCV13 as control | PCV13 (subcuta-neously) & PCV20 (both subcuta-neously and intramu-scularly) | 3 | Infants | Japan | 3+1 (2-3-4-12~15 months) | Both | Post dose 3, post booster | Pfizer dLIA | 4, 6B, 9V, 14, 18C, 19F, 23F, 1, 3, 5, 6A, 7F, 19A, 22F, 33F, 8, 10A, 11A, 12F,15B |
| 107 | Senders, 2024[214], NCT04382326[215], EUCTR2019-003305-10[216] | Phase III RCT, PCV13 as control | PCV13 & PCV20 | 2 | Infants | United States, Puerto Rico | 3+1 (2-4-6-12~15 months) | Both | Post dose 3, post booster | Pfizer dLIA | 4, 6B, 9V, 14, 18C, 19F, 23F, 1, 3, 5, 6A, 7F, 19A, 22F, 33F, 8, 10A, 11A, 12F,15B |
| 108 | Clarke, 2020[217], NCT02308540[218] | Phase I and II RCT, PCV13 as control | PCV10-SII & PCV13 | 3 | Infants | Gambia | 3+1 (2-3-4-10 months or 2-3-4-10~14 months) | Both | Post dose 3, post-booster | 3rd-gen ELISA (WHO reference ELISA) | 1, 5, 6A, 6B, 7F, 9V, 14, 19A, 19F, 23F |
| 109 | Clarke, 2021[219], NCT03197376[220] | Phase III RCT | PCV10-SII | 1 | Infants | Gambia | 3+1 (1.5-2.5-3.5-9 months) | Both | Post dose 3, post-booster | 3rd-gen ELISA (WHO reference ELISA) | 1, 5, 6A, 6B, 7F, 9V, 14, 19A, 19F, 23F |
| 110 | Adigweme, 2023[221], NCT03896477[222] | Phase III RCT | PCV10-SII & PCV13 | 2 | Infants | Gambia | 2+1 (1.5-3.5-9~18 months) | Both | Post dose 2, post-booster | 3rd-gen ELISA (WHO reference ELISA) | 1, 5, 6A, 6B, 7F, 9V, 14, 19A, 19F, 23F |
| Quasi-experimental studies | | | | | | | | | | | |
| 111 | Nurkka, 2001[223] | Phase II study, non-randomized study | PCV7 and PCV7 as primary PPSV23 as booster | 1 | Infants | Finland | 3+1 (2-4-6-15 months) | GMC | Post dose 3, post-booster | 1st-gen ELISA | 4, 6B, 9V, 14, 18C, 19F, 23F |
| 112 | Käyhty, 2005[224] | Single-arm, non-randomized study | PCV7 | 1 | Infants | Sweden | 2+1 (3-5-12 months) | GMC | Post dose 2, post-booster | 2nd-gen ELISA | 4, 6B, 9V, 14, 18C, 19F, 23F |
| 113 | Shao, 2006[225] | Single-arm, non-experimental study | PCV7 | 1 | Infants | Taiwan | 3+1 (2-4-6-15~20 months) | GMC | Post-booster | 2nd-gen ELISA | 4, 6B, 9V, 14, 18C, 19F, 23F |
| 114 | Kim, 2007[226] | Single-arm, non-experimental study | PCV7 | 1 | Infants | South Korea | 3+1 (2-4-6-12 months) | Both | Post dose 2, post dose 3 | 2nd-gen ELISA | 4, 6B, 9V, 14, 18C, 19F, 23F |
| 115 | Lee, 2009[227] | Single-arm, non-randomized study | PCV7 | 1 | Infants who monitored at Ewha Winans University Hospital | Korea | 3+0 (2-4-6 months) | Both | Post dose 3 | 3rd-gen ELISA (WHO reference ELISA) | 4, 6B, 9V, 14, 18C, 19F, 23F |
| 116 | Li, 2015[228] | Phase IV trial, open label, grouping based on age, single arm per age group | PCV7 | 2 | Infants | China | 3+1 (5-6-7-14 months) or 2+1 (10-11-15 months) | Both | Post-booster | 3rd-gen ELISA (WHO reference ELISA) | 4, 6B, 9V, 14, 18C, 19F, 23F |

| ID | Author & Year | Study design | Vaccine type | Study arm | Setting | | | Outcome reported | | | |
|---|---|---|---|---|---|---|---|---|---|---|---|
| | | | | | Population | Country/region | Schedule | Type† | Time points* | Assay | Serotype |
| 117 | Togashi, 2013[229], NCT00574795[230] | Single-arm, non-experimental, open label study | PCV13 (subcuta-neously) | 1 | Infants | Japan | 3+1 (3-4-5-12~15 months) | Both | Post dose 3, post-booster | 3rd-gen ELISA (WHO reference ELISA) | 4, 6B, 9V, 14, 18C, 19F, 23F, 1, 3, 5, 6A, 7F, 19A |
| 118 | Rodgers, 2013[93], Gutiérrez Brito, 2013[231], NCT00708682[232] | Single-arm, open-label study | PCV13 | 1 | Infants | Mexico | 3+1 (2-4-6-12 months) | Both | Post dose 2, post dose 3, post-booster | 3rd-gen ELISA (WHO reference ELISA) | 4, 6B, 9V, 14, 18C, 19F, 23F, 1, 3, 5, 6A, 7F, 19A |
| 119 | Singleton, 2013[233] | Phase III trial, open label, grouping based on age, single arm per age group | PCV13 | 1 | Alaska Native children | United States | 3+1 (2-4-6-12 months) | Rate | Post dose 3 | 3rd-gen ELISA (WHO reference ELISA) | 4, 6B, 9V, 14, 18C, 19F, 23F, 1, 3, 5, 6A, 7F, 19A |
| 120 | Wijmenga-Monsuur, 2015[234], van Westen, 2015[235], van Westen, 2018[126] | Non-randomized study phase IV trial | PCV13 | 1 | Infants | Netherlands | 3+1 (2-3-4-11 months) | Both | Post-booster | FMIA | 4, 6B, 9V, 14, 18C, 19F, 23F, 1, 3, 5, 6A, 7F, 19A |
| 121 | Martinón-Torres, 2015[236], Martinón-Torres, 2017[237], NCT01193335[238] | Phase IV trial, open label, grouping based on GA at birth, single arm per group | PCV13 | 1 | Infants | Poland, Spain | 3+1 (2-3-4-12 months) | Both | Post dose 3, post booster | 3rd-gen ELISA (WHO reference ELISA) | 4, 6B, 9V, 14, 18C, 19F, 23F, 1, 3, 5, 6A, 7F, 19A |
| 122 | Chu, 2023[239], NCT03574389[240] | Phase III trial, single-arm, open-label | PCV13 | 2 | Infants | China | 3+1 (2-4-6-12~15 months) or 2+1 (10-11-14 months) | Both | Post dose 3, post booster | Not mentioned | 4, 6B, 9V, 14, 18C, 19F, 23F, 1, 3, 5, 6A, 7F, 19A |
| 123 | Urbancikova, 2017[241] | Phase III trial, open label, grouping based on vaccine history | PCV13 | 2 | Infants | Czech Republic | 3+1 (2-3-4-12~15 months) or 2+1 (2-4-11~12 months) | Both | Post-booster | 3rd-gen ELISA (WHO reference ELISA) | 4, 6B, 9V, 14, 18C, 19F, 23F, 1, 3, 5, 6A, 7F, 19A |
| 124 | Maestri, 2024[242], NCT04633226[243] | Single-arm phase III trial | PCV15 | 1 | Infants | Korea | 3+1 (2-4-6-12~15 months) | Both | Post dose 3, post booster | Pn ECL | 4, 6B, 9V, 14, 18C, 19F, 23F, 1, 3, 5, 6A, 7F, 19A, 22F, 33F |
| Cohort studies | | | | | | | | | | | |
| 125 | O'Brien, 2000[244] | Prospective cohort study | PCV7 | 1 | SCD and matched healthy infants (SCD excluded) | United States | 3+0 (2-4-6 months) | GMC | post dose 3 | 2nd-gen ELISA | 4, 6B, 9V, 14, 18C, 19F, 23F |
| 126 | Esposito, 2005[245] | Prospective cohort study nested within a cluster-RCT | PCV7 | 1 | Preterm and term infants | Italy | 2+1 (3-5-11 months) | Rate | Post dose 2, post-booster | 2nd-gen ELISA | 4, 6B, 9V, 14, 18C, 19F, 23F |
| 127 | Osendarp, 2007[246] | Prospective cohort study nested within a cluster-RCT | PCV7 | 1 | Infants with or without zinc supplementation | Bangladesh | 3+0 (4-6-7 months) | GMC | Post dose 1, post dose 2, post dose 3 | 2nd-gen ELISA | 4, 6B, 9V, 14, 18C, 19F, 23F |

| ID | Author & Year | Study design | Vaccine type | Study arm | Setting | | | Outcome reported | | | |
|---|---|---|---|---|---|---|---|---|---|---|---|
| | | | | | Population | Country/region | Schedule | Type† | Time points* | Assay | Serotype |
| 128 | Vesikari, 2010[247] | Prospective cohort study nested within a RCT | PCV7 | 2 | Infants | France, Germany | 3+1 (2-3-4/12-15 months) | Both | Post dose 3 | 3rd-gen ELISA (WHO reference ELISA) | 4, 6B, 9V, 14, 18C, 19F, 23F |
| 129 | Moss, 2010[248], Moss, 2010[249] | Prospective cohort study | PCV7 | 2 | Healthy term infants | United Kingdom | 3+0 (2-3-4 months) or 3+1 (2-3-4-12 months) | Both | Post dose 3, post-booster | 3rd-gen ELISA (WHO reference ELISA) | 4, 6B, 9V, 14, 18C, 19F, 23F |
| 130 | Whelan, 2012[250] | Prospective cohort study | PCV7 | 1 | Children who were already receiving routine vaccinations as part of the national immunization schedule | Netherlands | 3+1 (2-3-4-11 months) | Both | Post-booster | 3rd-gen ELISA (WHO reference ELISA) | 4, 6B, 9V, 14, 18C, 19F, 23F |
| 131 | Jones, 2013[251] | Prospective cohort study | PCV7 | 1 | Healthy, HIV-unexposed infants | South Africa | 2+1 (1.5-3.5-9 months) | Both | Post dose 1, post dose 2, post-booster | 3rd-gen ELISA (WHO reference ELISA) | 4, 6B, 9V, 14, 18C, 19F, 23F |
| 132 | Madhi, 2013[252], Madhi et all. 2010[253], Madhi, 2020[254] | Prospective cohort study | PCV7 | 1 | Infants with various HIV status and healthy infant cohorts | South Africa | 3+1 (1.5-2.5-3.5-15~18 months) | Both | Post dose 1, post dose 2, post dose 3, post-booster | 3rd-gen ELISA (WHO reference ELISA) | 4, 6B, 9V, 14, 18C, 19F, 23F |
| 133 | Ladhani, 2015[255] | Prospective cohort study | PCV13 | 1 | Infants born at term who were immunised according to the national schedule | United Kingdom | 2+1 (2-4-12 months) | Both | Post dose 2 | 3rd-gen ELISA (WHO reference ELISA) | 4, 6B, 9V, 14, 18C, 19F, 23F, 1, 3, 5, 6A, 7F, 19A |
| 134 | Ladhani, 2015[256] | Prospective cohort study | PCV13 | 1 | Infants born at term who were immunised according to the national schedule | United Kingdom | 2+1 (2-4-12 months) | Both | Post dose 2 | 3rd-gen ELISA (WHO reference ELISA) | 4, 6B, 9V, 14, 18C, 19F, 23F, 1, 3, 5, 6A, 7F, 19A |
| 135 | Maertens, 2017[257] | Prospective cohort study nested within a cluster-randomized trials | PCV13 | 2 | Infants born to Tdap vaccinated mothers and controls | Belgium | 2+1 (2-4-12 months) | Both | Post dose 2, post-booster | 3rd-gen ELISA (WHO reference ELISA) | 4, 6B, 9V, 14, 18C, 19F, 23F, 1, 3, 5, 6A, 7F, 19A |
| 136 | Madhi, 2017[258] | Prospective cohort study nested within a phase Ib and II RCT | PCV13 | 1 | Infants born to GBS vaccinated mothers and controls | South Africa | 3+1 (1.5-2.5-3.5-9 months) | Both | Post dose 3, post booster | 3rd-gen ELISA (WHO reference ELISA) | 4, 6B, 9V, 14, 18C, 19F, 23F, 1, 3, 5, 6A, 7F, 19A |
| 137 | Zimmermann, 2020[259], Zimmermann, 2019[260], Zimmermann, 2019[261] | Prospective cohort study nested within a cluster-randomized trials | PCV13 | 2 | Infants randomised to receive neonatal BCG vaccination or no intervention | Australia | 3+0 (1.5-4-6 months) | Both | Post dose 3 | FMIA | 4, 6B, 9V, 14, 18C, 19F, 23F, 1, 3, 5, 6A, 7F, 19A |
| 138 | Perrett, 2020[262], Martinón-Torres, 2021[263], | Prospective cohort study nested | PCV13 | 4 | Infants born to Tdap vaccinated | Australia, Canada, Czech Republic, Finland, Italy, Spain | 3+1 (2-4-6-11~18 months) or 2+1 (3-5-11~18 months) | Both | Post dose 2, post dose 3, post-booster | Pn ECL | 4, 6B, 9V, 14, 18C, 19F, 23F, |

| ID | Author & Year | Study design | Vaccine type | Study arm | Setting | | | Outcome reported | | | |
|---|---|---|---|---|---|---|---|---|---|---|---|
| | | | | | Population | Country/region | Schedule | Type† | Time points* | Assay | Serotype |
| | NCT02422264[264], NCT02853929[265] | within a cluster-randomized trials | | | mothers and controls | | | | | | 1, 3, 5, 6A, 7F, 19A |

Note: GMC = geometric mean concentration; 1st-gen ELISA = first-generation enzyme-linked immunosorbent assay; 2nd-gen ELISA = second-generation enzyme-linked immunosorbent assay; 3rd-gen ELISA = third-generation enzyme-linked immunosorbent assay; WHO reference ELISA = World Health Organization enzyme-linked immunosorbent assay; GSK 22F-ELISA = GlaxoSmithKline 22F inhibition enzyme-linked immunosorbent assay; MIA = multiplex immunoassay; Pfizer dLIA = Pfizer direct Luminex immunoassay; Pn ECL = pneumococcal electrochemiluminescence assay.

†: “GMC” refers to IgG GMCs measured at specific time points post-vaccination; “Rate” refers to the seroresponse rate; and “Both” indicates that both outcomes are reported.

*: 30 days (4–6 weeks) post each sampling time point.

## Supplementary Table 13. Variable list and completeness analysis

| Variable name | Definition/details | Type | Completeness (N = 4419)* |
|---|---|---|---|
| title | Title of publication paper(s) | String | 4419 (100%) |
| Author_year | Author and publication year of paper(s) | String | 4419 (100%) |
| study_design_type | Study design type of this study arm | String | 4419 (100%) |
| study_sponsor | Sponsor of this study | String | 4391 (99.4%) |
| study_period | The period during which the study was conducted | String | 4419 (100%) |
| country | Country name where study was conducted | String | 4419 (100%) |
| ISO3 | Three-letter country codes | String | 4419 (100%) |
| WHO_region | Region of study sites based on WHO regions. The WHO regions are focused on public health administration and epidemiology. WHO divides the world based on health challenges, needs, and capacities to better coordinate global health initiatives and responses to diseases, including African Region (AFR), Region of the Americas (AMR), South-East Asian Region (SEAR), European Region (EUR), Eastern Mediterranean Region (EMR), and Western Pacific Region (WPR). | String | 4419 (100%) |
| Income_group | The World Bank classifies economies for analytical purposes into four income groups: low, lower-middle, upper-middle, and high income. | String | 4419 (100%) |
| sample_size | The sample size for immune response for certain vaccine, schedule, serotype and sampling time | Integer | 4419 (100%) |
| study_eligibility_minimum_age | The minimum age requirement to participant in the study | String | 4419 (100%) |
| study_eligibility_maximum_age | The maximum age requirement to participant in the study | String | 4370 (98.9%) |
| whe_vaccine_naive | Whether participant is naive to PCVs or not | Boolean | 4419 (100%) |
| percentage_vaccine_naive | The percentage of vaccine naive participants | Numeric | 4419 (100%) |
| percentage_female | The percentage of female participants | Numeric | 4169 (94.3%) |
| indigenous_status | Study conducted specifically for Indigenous population or not | Boolean | 4419 (100%) |
| ethnicity_or_race | The percentage of participants identifying their ethnicity as White | Boolean | 4419 (100%) |
| ethnicity_race_proportion | The percentage of participants identifying their race / ethnicity | String | 3281 (74.2%) |
| vaccine_type | Vaccine used througout the study in order | String | 4419 (100%) |
| administration_vaccine | Route of vaccine administration, given intramuscularly, or subcutaneously | String | 4419 (100%) |
| vaccine_schedule | Vaccine schedule | String | 4419 (100%) |
| vaccine_schedule_time | Timing of scheduled dose | String | 4419 (100%) |
| age_first_dose_primary | Timing of first dose of primay series | String | 4406 (99.7%) |
| age_last_dose_primary | Timing of last dose of primay series | Numeric | 4263 (96.5%) |
| vaccine_interval_primary | Time interval between primary series | Numeric | 4263 (96.5%) |
| timing_booster | Timing of booster dose | Numeric | 4419 (100%) |
| serotype | Serotype | String | 4419 (100%) |
| sampling_time | Time points of blood sample date post vaccination to get the antibody levels | String | 4419 (100%) |
| laboratory_assay | Assays used to measure IgG antibody | String | 4419 (100%) |
| results_mean | Mean value of IgG GMCs | String | 4308 (97.5%) |
| results_lower | Lower 95% confidence intervals for the IgG GMCs | Numeric | 4296 (97.2%) |
| results_upper | Upper 95% confidence intervals for the IgG GMCs | Numeric | 4296 (97.2%) |
| seropositive_num | Number of participants with pneumococcal serotype-specific IgG concentrations higher than predefined threshold | Numeric | 3520 (79.7%) |
| seropositive_rate | Percentage of participant with pneumococcal serotype-specific IgG concentrations higher than predefined threshold | Numeric | 3520 (79.7%) |

## Supplementary Table 14. Risk of bias assessment according to the modified JBI tool for randomized controlled trial (RCT)

| ID | Author & Year | Q3 | Q6 | Q8 | Q9 | Q10 | Modified Q11 | Q12 | Overall |
|---|---|---|---|---|---|---|---|---|---|
| 1 | Rennels, 1998[16] | Yes | Yes | Yes | Yes | No | Yes | Yes | High |
| 2 | Shinefield, 1999[17] | Yes | Yes | Yes | Yes | Yes | Yes | Yes | Low |
| 3 | Black, 2000[18], US FDA Package Insert: Prevnar for Prevnar Study D118-P8[19] | Unclear | Yes | Yes | Yes | No | Yes | Yes | High |
| 4 | Eskola, 2001[20], Ekström, 2005[21], Ekström, 2007[22] | Yes | Yes | Yes | Yes | Yes | Yes | Yes | Low |
| 5 | Tichmann-Schumann, 2005[23] | Unclear | Yes | Yes | Yes | Yes | Yes | Yes | Low |
| 6 | Scheifele, 2006[24], Scheifele, 2007[25] | Yes | Yes | Yes | Unclear | Yes | Yes | Yes | High |
| 7 | Knuf, 2006[26] | Yes | Yes | Yes | Yes | Yes | Yes | Yes | Low |
| 8 | Pichichero, 2007[27] | Yes | Yes | Yes | Yes | Yes | Yes | Yes | Low |
| 9 | O'Brien, 2007[28], Millar, 2007[29] | Yes | Yes | Yes | Yes | No | Yes | Yes | High |
| 10 | Li, 2008[30], NCT00488826[31], Li, 2016[32] | Yes | Yes | Yes | Yes | Yes | Yes | Yes | Low |
| 11 | Olivier, 2008[33] | Yes | Yes | Yes | Yes | No | No | Yes | High |
| 12 | Dennehy, 2008[34] | Yes | Yes | Yes | Yes | No | Yes | Yes | High |
| 13 | Trofa, 2008[35], NCT00197002[36] | Yes | Yes | Yes | Yes | Yes | Yes | Yes | Low |
| 14 | Vesikari, 2009[37], NCT00370396[38] | Yes | Yes | Yes | Yes | Yes | Yes | Yes | Low |
| 15 | Wysocki, 2009[39] | Yes | Yes | Yes | Yes | Yes | Yes | Yes | Low |
| 16 | Bermal, 2009[40], Bermal, 2011[41], NCT00344318[42], NCT00547248[43] | Yes | Yes | Yes | Yes | Yes | Yes | Yes | Low |
| 17 | Givon-Lavi, 2010[44], Dagan, 2010[45], Dagan, 2012[46], Dagan, 2018[47] | Yes | Yes | Yes | Yes | No | Yes | Yes | Moderate |
| 18 | Wysocki, 2010[48] | Yes | Yes | Yes | Yes | Yes | Yes | Yes | Low |
| 19 | Goldblatt, 2010[49] | Yes | Yes | Yes | Yes | Yes | Yes | Yes | Low |
| 20 | Grimprel, 2011[50] | Yes | Yes | Yes | Yes | Yes | Yes | Yes | Low |
| 21 | Scott, 2011[51] | Yes | Yes | Yes | Yes | Yes | Yes | Yes | Low |
| 22 | van den Bergh, 2011[52], van den Bergh, 2016[53], NCT00652951[54] | Yes | Yes | Yes | Yes | Yes | Yes | Yes | Low |
| 23 | Kim, 2011[55], NCT00680914[56], NCT00911144[57] | Yes | Yes | Yes | Yes | Yes | Yes | Yes | Low |
| 24 | Leonardi, 2011[58], NCT00109343[59] | Yes | Yes | Yes | Yes | Yes | Yes | Yes | Low |
| 25 | Marshall, 2011[60] | Yes | Yes | Yes | Yes | No | Yes | Yes | Low |
| 26 | Blatter, 2012[61], NCT00578175[62] | Yes | Yes | Yes | Yes | Yes | Yes | Yes | Low |
| 27 | Klein, 2012[63], NCT00474526[64] | Yes | Yes | Yes | Unclear | Yes | Yes | Yes | High |
| 28 | Tapiéro, 2013[65], Halperin et al, 2014[66] | Yes | Yes | Yes | Yes | Yes | Yes | Yes | Low |
| 29 | Vesikari, 2013[67], NCT00657709[68] | Yes | Yes | Yes | Unclear | Yes | No | Yes | High |
| 30 | van Westen, 2013[69], Rodenburg, 2010[70] | Yes | Yes | Yes | Yes | Yes | Yes | Yes | Low |
| 31 | Yetman, 2013[71], NCT00312858[72] | Yes | Yes | Yes | Yes | Yes | Yes | Yes | Low |
| 32 | Prymula, 2014[73], Esposito, 2014[74] | Yes | Yes | Yes | Yes | Yes | Yes | Yes | Low |
| 33 | López, 2017[75], NCT01444781[76] | Yes | Yes | Yes | Unclear | Yes | Yes | Yes | High |
| 34 | Zhao, 2022[77] | Yes | Yes | Yes | Yes | Yes | Yes | Yes | Low |
| 35 | FDA Package Insert: Prevnar for Study D118-P16[19] | Unclear | Yes | Yes | Yes | Unclear | Yes | Unclear | High |
| 36 | EUCTR2007-004276-39[78] | Yes | Yes | Yes | Unclear | Unclear | Yes | Yes | High |
| 37 | NCT01250756[79] | Yes | Yes | Yes | Unclear | Unclear | Yes | Yes | High |
| 38 | Thisyakorn, 2014[80] | Yes | Yes | Yes | Yes | Yes | Yes | Yes | Low |
| 39 | Grimprel, 2011[81], NCT00366678[82], NCT01026038[83] | Yes | Yes | Yes | Yes | Yes | Yes | Yes | Low |
| 40 | Sobanjo-ter Meulen, 2015[84], EUCTR2009-015103-58-FI[85], NCT01215175[86] | Yes | Yes | Yes | Yes | Yes | Yes | Yes | Low |
| 41 | Kieninger , 2010[87], NCT00366340[88], EUCTR2005-004770-24[89] | Yes | Yes | Yes | Yes | Yes | Yes | Yes | Low |
| 42 | Esposito, 2010[90], NCT00366899[91], EUCTR2005-004771-38-IT[92], Rodgers, 2013[93] | Yes | Yes | Yes | Yes | Yes | Yes | Yes | Low |
| 43 | Snape, 2010[94]; Rodgers, 2013[93], NCT00384059[95], EUCTR2005-005130-12[96] | Yes | Yes | Yes | Yes | Yes | Yes | Yes | Low |
| 44 | Bryant, 2010[97], NCT00205803[98] | Yes | Yes | Yes | Yes | Yes | Yes | Yes | Low |

| ID | Author & Year | Q3 | Q6 | Q8 | Q9 | Q10 | Modified Q11 | Q12 | Overall |
|---|---|---|---|---|---|---|---|---|---|
| 45 | Yeh, 2010[99], NCT00373958[100] | Yes | Yes | Yes | Yes | Yes | Yes | Yes | Low |
| 46 | Weckx, 2012[101], NCT00676091[102] | Yes | Yes | Yes | Yes | Yes | Yes | Yes | Low |
| 47 | Huang, 2012[103], NCT00688870[104] | Yes | Yes | Yes | Yes | Yes | Yes | Yes | Low |
| 48 | Amdekar, 2013[105] | Yes | Yes | Yes | Yes | No | Yes | Yes | Moderate |
| 49 | Dagan, 2013[106], Juergens, 2014[107], Dagan, 2021[108] | Yes | Yes | Yes | Yes | Yes | Yes | Yes | Low |
| 50 | Kim, 2013[109], NCT00689351[110] | Yes | Yes | Yes | Yes | Yes | Yes | Yes | Low |
| 51 | Rodgers, 2013[93]; Diez-Domingo, 2013[111], NCT00368966[112] | Yes | Yes | Yes | Yes | Yes | Yes | Yes | Low |
| 52 | Payton, 2013[113], NCT00444457[114] | Yes | Yes | Yes | Yes | Yes | Yes | Yes | Low |
| 53 | Togashi, 2015[115], NCT01200368[116] | Yes | Yes | Yes | Yes | Yes | Yes | Yes | Low |
| 54 | Zhu, 2016[117], Zhu, 2019[118] | Yes | Yes | Yes | Yes | Yes | Yes | Yes | Low |
| 55 | NCT00452790[119] | Yes | Yes | Yes | Yes | Yes | Yes | Yes | Low |
| 56 | Gadzinowski, 2011[120], NCT00464945[121], EUCTR2006-006204-11[122] | Yes | Yes | Yes | Yes | Yes | Yes | Yes | Low |
| 57 | Vanderkooi, 2012[123], NCT00475033[124] | Yes | Yes | Yes | Yes | Yes | Yes | Yes | Low |
| 58 | Spijkerman, 2013[125], van Westen, 2018[126] | Yes | Yes | Yes | Yes | Yes | Yes | Yes | Low |
| 59 | Rodgers, 2013[93], Martinón-Torres, 2012[127], NCT00474539[128], EUCTR2007-000304-32[129] | Yes | Yes | Yes | Yes | Yes | Yes | Yes | Low |
| 60 | Gadzinowski, 2015[130], NCT00366548[131] | Yes | Yes | Yes | Yes | Yes | Yes | Yes | Low |
| 61 | Iro, 2015[132] | Yes | Yes | Yes | Yes | No | Yes | Yes | Moderate |
| 62 | Truck, 2016[133] | Yes | Yes | Yes | Yes | Yes | Yes | Yes | Low |
| 63 | Block, 2016[134] | Yes | Yes | Yes | Yes | No | Yes | Yes | Moderate |
| 64 | Prymula, 2017[135], NCT01204658[136] | Yes | Yes | Yes | Yes | Yes | Yes | Yes | Low |
| 65 | Vesikari, 2017[137], NCT01248884[138], NCT01453998[138] | Yes | Yes | Yes | Yes | Yes | Yes | Yes | Low |
| 66 | Idoko, 2017[139], NCT01964716[140], EUCTR2012-000482-21[141] | Yes | Yes | Yes | Yes | Yes | Yes | Yes | Low |
| 67 | Wysocki, 2017[142], NCT01392378[143], EUCTR2010-022303-22[144] | Yes | Yes | Yes | Yes | Yes | No | Yes | Low |
| 68 | Cutland, 2018[145], NCT01939158[146] | Yes | Yes | Yes | Yes | Yes | Yes | Yes | Low |
| 69 | Prymula, 2018[147], EUCTR2012-001055-39[148], EUCTR2012-001042-18-ES[149] | Yes | Yes | Yes | Yes | Yes | Yes | Yes | Low |
| 70 | Goldblatt, 2018[150], EUCTR2015-000817-32[151] | Yes | Yes | Yes | Yes | Yes | Yes | Yes | Low |
| 71 | Temple, 2019[152] | Yes | Yes | Yes | Yes | Yes | Yes | Yes | Low |
| 72 | Moïsi, 2019[153] | Yes | Yes | Yes | Yes | Yes | No | Yes | Low |
| 73 | Carmona Martinez, 2019[154], NCT01616459[155] | Yes | Yes | Yes | Yes | Yes | Yes | Yes | Low |
| 74 | Odutola, 2019[156], NCT01262872[157] | Yes | Yes | Yes | Yes | Yes | Yes | Yes | Low |
| 75 | Klein, 2019[158], NCT01978093[159] | No | Yes | Yes | Yes | Yes | Yes | Yes | Moderate |
| 76 | Madhi, 2020[160], Mutsaerts, 2024[161] | Yes | Yes | Yes | Yes | Yes | No | Yes | Low |
| 77 | Shin, 2020[162] | Yes | Yes | Yes | Yes | Yes | Yes | Yes | Low |
| 78 | Leach, 2021[163], Leach, 2022[164] | Yes | Yes | Yes | Yes | Yes | Yes | Yes | Low |
| 79 | Lalwani, 2021[165], NCT03548337[166] | Yes | Yes | Yes | Yes | Yes | Yes | Yes | Low |
| 80 | Dhingra, 2021[167], NCT03205371[168] | Yes | Yes | Yes | Yes | Yes | Yes | Yes | Low |
| 81 | Wang, 2022[169] | Yes | Yes | Yes | Yes | Yes | Yes | Yes | Low |
| 82 | Kawade, 2023[170] | Yes | Yes | Yes | Yes | Yes | No | Yes | Low |
| 83 | Sanchez, 2023[171] | Yes | Yes | Yes | Yes | Yes | Yes | Yes | Low |
| 84 | Rajan, 2023[172] | Yes | Yes | Yes | Yes | Yes | No | Yes | Low |
| 85 | Simon, 2023[173], NCT03550313[174] | Yes | Yes | Yes | Yes | Yes | Yes | Yes | Low |
| 86 | Temple, 2023[175] | Yes | Yes | Yes | Yes | Yes | No | Yes | Low |
| 87 | Xie, 2024[176] | Yes | Yes | Yes | Yes | Yes | Yes | Yes | Low |
| 88 | Matur, 2024[177] | Yes | Yes | Yes | Yes | Yes | Yes | Yes | Low |
| 89 | Gallagher, 2024[178] | Yes | Yes | Yes | Yes | Yes | Yes | Yes | Low |
| 90 | Borys, 2024[179] | No | Yes | Yes | Yes | Yes | Yes | Yes | Moderate |

| ID | Author & Year | Q3 | Q6 | Q8 | Q9 | Q10 | Modified Q11 | Q12 | Overall |
|---|---|---|---|---|---|---|---|---|---|
| 91 | NCT01090453[180] | Yes | Yes | Yes | Yes | Yes | Yes | Yes | Low |
| 92 | NCT03207750[181] | Yes | Yes | Yes | Yes | Yes | Yes | Yes | Low |
| 93 | Bili, 2023[182], NCT03620162[183], EUCTR2018-001151-12[184] | Yes | Yes | Yes | Yes | Yes | Yes | Yes | Low |
| 94 | NCT05408429[185], EUCTR2021-006624-41[186] | Yes | Yes | Yes | Yes | Yes | Yes | Yes | Low |
| 95 | Greenberg, 2018[187], NCT01215188[188], EUCTR2010-019775-29[189] | Yes | Yes | Yes | Yes | Yes | Yes | Yes | Low |
| 96 | Rupp, 2019[190], NCT02531373[191] | No | Yes | Yes | Yes | No | Yes | Yes | High |
| 97 | Platt, 2020[192], NCT02987972[193] | Yes | Yes | Yes | Yes | Yes | Yes | Yes | Low |
| 98 | Banniettis, 2022[194], EUCTR2018-003706-88[195], NCT03885934[196] | Yes | Yes | Yes | Yes | Yes | Yes | Yes | Low |
| 99 | Martinón-Torres, 2023[197], NCT04031846[198] | Yes | Yes | Yes | Yes | Yes | Yes | Yes | Low |
| 100 | Benfield, 2023[199], NCT04016714[200] | Yes | Yes | Yes | Yes | Yes | Yes | Yes | Low |
| 101 | Suzuki, 2023[201], NCT04384107[202] | Yes | Yes | Yes | Yes | Yes | Yes | Yes | Low |
| 102 | Ishihara, 2023[203], Wan, 2024[204], NCT03848065[205] | Yes | Yes | Yes | Yes | Yes | Yes | Yes | Low |
| 103 | Lupinacci, 2023[206], NCT03893448[207] | Yes | Yes | Yes | Yes | Yes | Yes | Yes | Low |
| 104 | Senders, 2021[208], NCT03512288[209] | Yes | Yes | Yes | Yes | Yes | Yes | Yes | Low |
| 105 | Korbal, 2024[210], NCT04546425[211] | Yes | Yes | Yes | Yes | Yes | Yes | Yes | Low |
| 106 | Ishihara, 2024[212], NCT04530838[213] | Yes | Yes | Yes | Yes | Yes | Yes | Yes | Low |
| 107 | Senders, 2024[214], NCT04382326[215], EUCTR2019-003305-10[216] | Yes | Yes | Yes | Yes | Yes | Yes | Yes | Low |
| 108 | Clarke, 2020[217], NCT02308540[218] | Yes | Yes | Yes | Yes | Yes | Yes | Yes | Low |
| 109 | Clarke, 2021[219], NCT03197376[220] | Yes | Yes | Yes | Yes | Yes | Yes | Yes | Low |
| 110 | Adigweme, 2023[221], NCT03896477[222] | Yes | Yes | Yes | Yes | Yes | Yes | Yes | Low |

## Supplementary Table 15. Risk of bias assessment according to the modified JBI tool for quasi experimental study

| ID | Author & Year | Q3 | Q4 | Q6 | Q7 | Q8 | Q9 | Overall |
|---|---|---|---|---|---|---|---|---|
| 111 | Nurkka, 2001[223] | Unclear | Yes | Yes | Yes | Yes | Yes | Moderate |
| 112 | Käyhty, 2005[224] | Single-arm | Single-arm | Single-arm | Yes | Yes | Yes | Low |
| 113 | Shao, 2006[225] | Single-arm | Single-arm | Single-arm | Yes | Yes | Yes | Low |
| 114 | Kim, 2007[226] | Single-arm | Single-arm | Single-arm | Yes | Yes | Yes | Low |
| 115 | Lee, 2009[227] | Single-arm | Single-arm | Single-arm | Yes | Yes | Yes | Low |
| 116 | Li, 2015[228] | Subjects were stratified into 4 groups based on age | Yes | Yes | Yes | Yes | Yes | Low |
| 117 | Togashi, 2013[229], NCT00574795[230] | Single-arm | Single-arm | Single-arm | Yes | Yes | Yes | Low |
| 118 | Rodgers, 2013[93], Gutiérrez Brito, 2013[231], NCT00708682[232] | Single-arm | Single-arm | Single-arm | Yes | Yes | Yes | Low |
| 119 | Singleton, 2013[233] | Subjects were stratified into 5 groups based on age | Yes | Yes | Yes | No | Yes | High |
| 120 | Wijmenga-Monsuur, 2015[234], van Westen, 2015[235], van Westen, 2018[126] | Subjects were stratified into 2 groups based on birth date | Yes | Yes | Yes | Yes | Yes | Low |
| 121 | Martinón-Torres, 2015[236], Martinón-Torres, 2017[237], NCT01193335[238] | Subjects were designated preterm or term based on GA at birth. | Yes | Yes | Yes | Yes | Yes | Low |
| 122 | Chu, 2023[239], NCT03574389[240] | Subjects were stratified into 4 groups based on age | Yes | Yes | Unclear | No | Yes | High |
| 123 | Urbancikova, 2017[241] | Yes | No | Yes | Yes | Yes | Yes | Low |
| 124 | Maestri, 2024[242], NCT04633226[243] | Single-arm | Single-arm | Single-arm | Yes | Yes | Yes | Low |

**Supplementary Table 16. Risk of bias assessment according to the modified JBI tool for cohort study**

| ID | Author & Year | Q1 | Q2 | Q7 | Q8 | Q9 | Q11 | Overall |
|---|---|---|---|---|---|---|---|---|
| 125 | O'Brien, 2000[244] | Yes | Yes | Yes | Yes | Yes | Yes | Low |
| 126 | Esposito, 2005[245] | Yes | Yes | Yes | Yes | Yes | Yes | Low |
| 127 | Osendarp, 2007[246] | Yes | Yes | Yes | Yes | Yes | Yes | Low |
| 128 | Vesikari, 2010[247] | Yes | Yes | Yes | Yes | Yes | Yes | Low |
| 129 | Moss, 2010[248], Moss, 2010[249] | Yes | Yes | Yes | Yes | No | Yes | Moderate |
| 130 | Whelan, 2012[250] | Yes | Yes | Yes | Yes | Yes | Yes | Low |
| 131 | Jones, 2013[251] | Single-arm | Single-arm | Yes | Yes | Yes | Yes | Low |
| 132 | Madhi, 2013[252], Madhi et all. 2010[253], Madhi, 2020[254] | No, but only HIV-uninfected infants born to HIV non-infected mothers were included | Yes | Yes | Yes | Yes | Yes | Low |
| 133 | Ladhani, 2015[255] | Single-arm | Single-arm | Yes | Yes | Yes | Partly yes, no details of method for calculating 95%CI | Moderate |
| 134 | Ladhani, 2015[256] | Single-arm | Single-arm | Yes | Yes | Yes | Partly yes, no details of method for calculating 95%CI | Moderate |
| 135 | Maertens, 2017[257] | Yes | Yes | Yes | Yes | Yes | Partly yes, no details of method for calculating 95%CI | Moderate |
| 136 | Madhi, 2017[258] | Yes | Yes | Yes | Yes | Yes | Yes | Low |
| 137 | Zimmermann, 2020[259], Zimmermann, 2019[260], Zimmermann, 2019[261] | Yes | Yes | Yes | Yes | No | Yes | Moderate |
| 138 | Perrett, 2020[262], Martinón-Torres, 2021[263], NCT02422264[264], NCT02853929[265] | Yes | Yes | Yes | Yes | Yes | Yes | Low |

## Supplementary Table 17. Distribution of included study arms by vaccine products and countries conducted

| Country* | Number of study arm by vaccine products | | | | |
|---|---|---|---|---|---|
| | PCV7 | PCV13 | PCV15 | PCV20 | PCV10-SII |
| Australia | - | 3 | - | - | - |
| Bangladesh | 1 | - | - | - | - |
| Belgium | - | 2 | - | - | - |
| Brazil | 1 | 1 | - | - | - |
| Burkina Faso | - | 2 | - | - | - |
| Canada | 5 | 1 | - | - | - |
| China | 8 | 8 | - | - | - |
| Czechia | - | 1 | - | - | - |
| Finland | 2 | - | - | - | - |
| France | 1 | 1 | - | - | - |
| Gambia (the) | - | 6 | - | - | 3 |
| Germany | 5 | 1 | - | - | - |
| India | 2 | 8 | - | - | - |
| Israel | 4 | 1 | - | - | - |
| Italy | 2 | 1 | - | - | - |
| Japan | 2 | 5 | 3 | 2 | - |
| Kenya | 2 | 1 | - | - | - |
| Korea (the Republic of) | 4 | 1 | 1 | - | - |
| Mexico | - | 1 | - | - | - |
| Multi-countries | 18 | 31 | 7 | 2 | - |
| Netherlands (the) | 4 | 5 | - | - | - |
| Philippines (the) | 1 | - | - | - | - |
| Poland | 3 | 4 | - | - | - |
| Russian Federation (the) | - | 2 | - | - | - |
| Slovakia | - | 1 | - | - | - |
| South Africa | 2 | 4 | - | - | - |
| Spain | 1 | 2 | - | - | - |
| Sweden | 1 | - | - | - | - |
| Thailand | 1 | 3 | - | - | - |
| United Kingdom of Great Britain and Northern Ireland (the) | 6 | 8 | - | - | - |
| United States of America (the) | 25 | 15 | 1 | 1 | - |
| Viet Nam | 1 | 2 | - | - | - |

*"Multi-countries" indicates study arm conducted across multiple sites involving more than one country.

## Supplementary Table 18. Detailed breakdown of countries involved in "Multi-countries" studies and corresponding number of study arms

| Country | Number of study arm by vaccine products | | | |
|---|---|---|---|---|
| | PCV7 | PCV13 | PCV15 | PCV20 |
| Argentina | 5 | - | - | - |
| Australia | - | 8 | 1 | 1 |
| Austria | 2 | - | - | - |
| Belgium | - | 2 | 1 | 1 |
| Canada | - | 13 | 2 | - |
| Chile | 3 | - | - | - |
| Colombia | 5 | - | - | - |
| Costa Rica | 3 | - | - | - |
| Czechia | 5 | 12 | 1 | 1 |
| Denmark | - | 3 | 3 | 1 |
| Dominican Republic (the) | - | 3 | - | - |
| Estonia | - | 2 | 1 | 1 |
| Finland | 4 | 11 | 4 | 1 |
| France | 6 | 2 | - | - |
| Germany | 6 | 7 | 1 | - |
| Greece | - | 1 | 1 | - |
| Hungary | 3 | 1 | - | - |
| Israel | - | 1 | 2 | - |
| Italy | 5 | 6 | 1 | 1 |
| Malaysia | - | 1 | 1 | - |
| Malta | - | 2 | - | - |
| Netherlands (the) | - | 1 | - | 1 |
| Norway | - | 2 | 1 | 1 |
| Panama | - | 2 | - | - |
| Poland | 4 | 7 | 2 | 1 |
| Puerto Rico | - | 3 | 2 | 1 |
| Russian Federation (the) | - | 3 | 2 | 1 |

| Country | Number of study arm by vaccine products | | | |
|---|---|---|---|---|
| | PCV7 | PCV13 | PCV15 | PCV20 |
| Slovakia | - | 1 | - | 1 |
| South Africa | - | 2 | - | - |
| Spain | 1 | 10 | 3 | - |
| Sweden | - | 1 | - | - |
| Thailand | - | 3 | 3 | - |
| Turkey | - | 4 | 2 | - |
| United Kingdom of Great Britain and Northern Ireland (the) | - | 2 | - | - |
| United States of America (the) | 3 | 7 | 2 | 1 |

### Supplementary Table 19. Summary of study arm inclusion and exclusion for the primary analysis

| Step | Number of Study Arms | Description |
|---|---|---|
| Total study arms identified | 243 | All study arms in the analysis dataset |
| Excluded from main analysis* | 81 | Study arms excluded based on pre-specified criteria |
| Late priming | 9 | Participants received first primary dose >4 months of age or last primary dose >6 months |
| Subcutaneous administration | 10 | Study arms where vaccines were administered subcutaneously |
| Assay exclusion | 52 | Outcomes measured using first-generation ELISA, GSK 22F-ELISA, ELISA without detail, or fluorescent bead-based multiplex immunoassay |
| High risk of bias | 28 | Study arms assessed as high risk of bias |
| Included in main analysis | 162 | Study arms retained after applying all exclusion criteria (age criteria met, intramuscular, eligible assay, low/moderate risk) |

*Numbers for individual exclusion criteria may exceed the total number excluded because some study arms met more than one exclusion criterion. These study arms were retained for sensitivity analyses.

### Supplementary Table 20. Number of study arms reporting IgG geometric mean concentrations (IgG GMCs) and seroresponse rates post-childhood-schedule by vaccine product

| Vaccine product | Num of study arms with reported IgG GMCs | Num of study arms with reported seroresponse rates |
|---|---|---|
| PCV7 | 47 | 23 |
| PCV13 | 77 | 58 |
| PCV15 | 8 | 3 |
| PCV20 | 4 | 3 |
| PCV10-SII | 3 | 1 |

### Supplementary Table 21. Number of study arms reporting IgG geometric mean concentrations (IgG GMCs) and seroresponse rates post-childhood-schedule by vaccine product and schedule

| Vaccine product | Vaccine schedule | Num of study arms with reported IgG GMCs | Num of study arms with reported seroresponse rates |
|---|---|---|---|
| PCV7 | 3+1 | 34 | 16 |
| | 2+1 | 7 | 4 |
| | 1+1 | - | - |
| | 3+0 | 6 | 3 |
| PCV13 | 3+1 | 46 | 26 |
| | 2+1 | 19 | 19 |
| | 1+1 | 7 | 7 |
| | 3+0 | 5 | 6 |
| PCV15 | 3+1 | 6 | 1 |
| | 2+1 | 2 | 2 |
| | 1+1 | - | - |
| | 3+0 | - | - |
| PCV20 | 3+1 | 3 | 2 |
| | 2+1 | 1 | 1 |
| | 1+1 | - | - |
| | 3+0 | - | - |
| PCV10-SII | 3+1 | 2 | - |
| | 2+1 | 1 | 1 |
| | 1+1 | - | - |
| | 3+0 | - | - |

### Supplementary Table 22. Number of study arms reporting IgG geometric mean concentrations (IgG GMCs) and seroresponse rates post “3+1” vaccination schedule by vaccine product and WHO region

| Vaccine product | WHO region | Num of study arms with reported IgG GMCs | Num of study arms with reported seroresponse rates |
|---|---|---|---|
| PCV7 | AFR | - | - |
| | AMR | 10 | 5 |
| | EUR | 13 | 6 |
| | SEAR | 3 | 2 |
| | WPR | 7 | 2 |
| | Multi | 1 | 1 |
| PCV13 | AFR | 3 | 1 |
| | AMR | 16 | 7 |
| | EUR | 10 | 7 |
| | SEAR | 4 | 4 |
| | WPR | 5 | 3 |
| | Multi | 8 | 4 |
| PCV15 | AFR | - | - |
| | AMR | - | - |
| | EUR | - | - |
| | SEAR | - | - |
| | WPR | 2 | 1 |
| | Multi | 4 | - |
| PCV20 | AFR | - | - |
| | AMR | 2 | 1 |
| | EUR | - | - |
| | SEAR | - | - |
| | WPR | 1 | 1 |
| | Multi | - | - |
| PCV10-SII | AFR | 2 | - |
| | AMR | - | - |
| | EUR | - | - |
| | SEAR | - | - |
| | WPR | - | - |
| | Multi | - | - |

***Abbreviations:*** AFR: African Region; AMR: Region of the Americas; EUR: European Region; SEAR: South-East Asia Region; WPR: Western Pacific Region; Multi: Multi regions.

### Supplementary Table 23. Number of study arms reporting IgG geometric mean concentrations (IgG GMCs) and seroresponse rates post different timepoints by vaccine product

| Vaccine product | Timepoint | Num of study arms with reported IgG GMCs | Num of study arms with reported seroresponse rates |
|---|---|---|---|
| PCV7 | Post 1-dose primary | 3 | 3 |
| | Post 2-dose primary | 11 | 11 |
| | Post 3-dose primary | 36 | 28 |
| | Post booster | 41 | 20 |
| PCV13 | Post 1-dose primary | 5 | 5 |
| | Post 2-dose primary | 21 | 23 |
| | Post 3-dose primary | 49 | 52 |
| | Post booster | 72 | 52 |
| PCV15 | Post 1-dose primary | - | - |
| | Post 2-dose primary | 2 | 2 |
| | Post 3-dose primary | 6 | 6 |
| | Post booster | 8 | 3 |
| PCV20 | Post 1-dose primary | - | - |
| | Post 2-dose primary | 1 | 1 |
| | Post 3-dose primary | 3 | 3 |
| | Post booster | 4 | 3 |
| PCV10-SII | Post 1-dose primary | - | - |
| | Post 2-dose primary | 1 | 1 |
| | Post 3-dose primary | 2 | 2 |
| | Post booster | 3 | 1 |

## Supplementary Table 24. Summary of the meta-analysis results of anti-pneumococcal IgG geometric mean concentrations (GMCs, μg/mL) post-childhood-schedule by vaccine product and serotype

| Serotype | IgG GMCs (95%CI) by vaccine product (μg/mL) | | | | |
|---|---|---|---|---|---|
| (ST) | PCV7 | PCV13 | PCV15 | PCV20 | PCV10-SII |
| PCV7 covered STs | | | | | |
| 4 | 4.02 (3.37, 4.79) | 3.73 (3.25, 4.30) | 1.58 (1.29, 1.93) | 5.32 (3.76, 7.52) | - |
| 6B | 6.92 (5.42, 8.84) | 7.13 (6.30, 8.07) | 5.70 (4.83, 6.72) | 4.73 (2.98, 7.52) | 10.76 (8.16, 14.20) |
| 9V | 3.45 (3.02, 3.93) | 3.23 (2.90, 3.60) | 2.73 (2.25, 3.31) | 4.38 (3.44, 5.59) | 2.47 (1.70, 3.60) |
| 14 | 10.55 (9.36, 11.90) | 9.47 (8.73, 10.28) | 6.86 (5.70, 8.27) | 6.69 (4.80, 9.33) | 7.49 (6.58, 8.52) |
| 18C | 3.10 (2.76, 3.49) | 3.00 (2.69, 3.36) | 2.73 (2.31, 3.23) | 3.74 (2.79, 5.02) | - |
| 19F | 4.54 (3.91, 5.27) | 8.07 (7.30, 8.92) | 4.62 (4.02, 5.32) | 6.71 (5.30, 8.51) | 8.09 (5.73, 11.43) |
| 23F | 4.90 (4.16, 5.76) | 4.17 (3.67, 4.73) | 2.26 (1.84, 2.77) | 4.64 (2.96, 7.27) | 4.54 (3.96, 5.21) |
| PCV13-non-PCV7 STs | | | | | |
| 1 | - | 4.26 (3.75, 4.85) | 1.71 (1.38, 2.11) | 2.07 (1.51, 2.82) | 6.76 (5.28, 8.65) |
| 3 | - | 0.98 (0.89, 1.08) | 1.06 (0.90, 1.24) | 0.84 (0.60, 1.17) | - |
| 5 | - | 3.00 (2.69, 3.34) | 2.99 (2.32, 3.86) | 2.38 (1.72, 3.29) | 1.64 (1.19, 2.26) |
| 6A | - | 7.98 (7.15, 8.90) | 4.24 (3.60, 4.98) | 10.73 (8.02, 14.36) | 7.49 (4.79, 11.72) |
| 7F | - | 5.21 (4.81, 5.64) | 3.71 (3.22, 4.27) | 4.52 (3.58, 5.71) | 6.48 (6.09, 6.90) |
| 19A | - | 8.17 (7.56, 8.83) | 5.11 (4.48, 5.84) | 5.17 (3.68, 7.25) | 6.13 (3.82, 9.82) |
| PCV15-non-PCV13 STs | | | | | |
| 22F | - | - | 7.94 (6.48, 9.72) | 11.89 (9.55, 14.82) | - |
| 33F | - | - | 4.47 (3.70, 5.40) | 7.47 (5.12, 10.90) | - |
| PCV20-non-PCV15 STs | | | | | |
| 8 | - | - | - | 4.01 (3.08, 5.23) | - |
| 10A | - | - | - | 6.98 (5.15, 9.45) | - |
| 11A | - | - | - | 4.55 (3.51, 5.90) | - |
| 12F | - | - | - | 2.04 (1.72, 2.44) | - |
| 15B | - | - | - | 16.00 (12.31, 20.80) | - |

Heterogeneity for PCV7: $I^2$=98.7%, $\tau^2$=0.462, p <0.01; for PCV13: $I^2$=99.3%, $\tau^2$=0.572, p <0.01; for PCV15: $I^2$=99.4%, $\tau^2$=0.361, p <0.01; for PCV20: $I^2$=99.6%, $\tau^2$=0.508, p <0.01; for PCV10-SII: $I^2$=99.1%, $\tau^2$=0.354, p <0.01.

## Supplementary Table 25. Summary of the meta-analysis results of anti-pneumococcal IgG seroresponse rates (%) post-childhood-schedule by vaccine product

| Serotype | Seroresponse rate (95%CI) by vaccine product (%) | | | | |
|---|---|---|---|---|---|
| (ST) | PCV7 | PCV13 | PCV15 | PCV20 | PCV10-SII† |
| PCV7 covered STs | | | | | |
| 4 | 98% (97%-99%) | 99% (98%-99%) | 96% (95%-97%) | 98% (91%-100%) | - |
| 6B | 98% (96%-99%) | 98% (97%-99%) | 98% (96%-99%) | 99% (98%-99%) | 100% (98%-100%) |
| 9V | 99% (98%-99%) | 98% (98%-99%) | 99% (98%-100%) | 99% (98%-99%) | 99% (96%-100%) |
| 14 | 99% (98%-99%) | 99% (98%-99%) | 99% (99%-100%) | 98% (94%-100%) | 98% (96%-100%) |
| 18C | 99% (98%-99%) | 98% (98%-99%) | 99% (98%-100%) | 99% (98%-99%) | - |
| 19F | 98% (97%-99%) | 98% (98%-99%) | 100% (99%-100%) | 99% (98%-100%) | 100% (98%-100%) |
| 23F | 98% (97%-99%) | 98% (97%-98%) | 97% (96%-98%) | 98% (94%-99%) | 98% (95%-99%) |

| Serotype (ST) | Seroresponse rate (95%CI) by vaccine product (%) | | | | |
|---|---|---|---|---|---|
| | PCV7 | PCV13 | PCV15 | PCV20 | PCV10-SII† |
| PCV13-non-PCV7 STs | | | | | |
| 1 | - | 99% (98%-99%) | 97% (95%-98%) | 97% (93%-98%) | 100% (98%-100%) |
| 3 | - | 91% (88%-94%) | 92% (91%-94%) | 84% (70%-92%) | - |
| 5 | - | 98% (98%-99%) | 99% (98%-100%) | 98% (97%-99%) | 98% (94%-99%) |
| 6A | - | 99% (98%-99%) | 99% (98%-99%) | 99% (98%-100%) | 98% (95%-99%) |
| 7F | - | 99% (99%-99%) | 100% (99%-100%) | 100% (99%-100%) | 100% (98%-100%) |
| 19A | - | 99% (99%-100%) | 99% (99%-100%) | 100% (99%-100%) | 100% (98%-100%) |
| PCV15-non-PCV13 STs | | | | | |
| 22F | - | - | 100% (99%-100%) | 99% (99%-100%) | - |
| 33F | - | - | 99% (98%-100%) | 99% (98%-100%) | - |
| PCV20-non-PCV15 STs | | | | | |
| 8 | - | - | - | 99% (99%-100%) | - |
| 10A | - | - | - | 98% (96%-99%) | - |
| 11A | - | - | - | 99% (98%-99%) | - |
| 12F | - | - | - | 97% (94%-98%) | - |
| 15B | - | - | - | 100% (99%-100%) | - |

†: Only 1 study arm available for PCV10-SII, no pooled estimate or heterogeneity.
Heterogeneity for PCV7: $I^2$=79.2%, $\tau^2$=0.972, p <0.01; for PCV13: $I^2$=84.7%, $\tau^2$=0.979, p <0.01; for PCV15: $I^2$=81.1%, $\tau^2$=0.595, p <0.01; for PCV20: $I^2$=95.2%, $\tau^2$=1.063, p <0.01.

**Supplementary Table 26. Summary of the meta-analysis results of anti-pneumococcal IgG geometric mean concentrations (IgG GMCs, µg/mL) post-childhood-schedule by vaccine product and schedule**

| Serotype | IgG GMCs (95%CI) by vaccine schedule (µg/mL) | | | |
|---|---|---|---|---|
| | 3+1 | 2+1 | 1+1 | 3+0 |
| PCV7 | | | | |
| 4 | 4.19 (3.41, 5.15) | 5.68 (4.23, 7.63) | - | 2.08 (1.61, 2.68) |
| 6B | 9.43 (8.15, 10.91) | 6.34 (4.38, 9.17) | - | 1.22 (0.53, 2.80) |
| 9V | 3.76 (3.35, 4.22) | 4.19 (3.10, 5.66) | - | 1.58 (1.17, 2.14) |
| 14 | 11.25 (9.95, 12.72) | 13.04 (11.09, 15.33) | - | 5.50 (4.17, 7.25) |
| 18C | 3.32 (2.90, 3.81) | 2.87 (2.25, 3.68) | - | 2.22 (1.58, 3.11) |
| 19F | 5.01 (4.37, 5.74) | 5.81 (4.64, 7.28) | - | 1.89 (1.26, 2.85) |
| 23F | 5.93 (5.14, 6.83) | 4.50 (3.64, 5.57) | - | 1.67 (1.25, 2.24) |
| PCV13 | | | | |
| 4 | 3.51 (2.90, 4.26) | 3.47 (2.68, 4.49) | 6.05 (4.03, 9.10) | 4.33 (3.29, 5.70) |
| 6B | 9.07 (8.17, 10.07) | 6.75 (5.54, 8.23) | 3.12 (2.42, 4.00) | 2.64 (1.73, 4.04) |
| 9V | 3.10 (2.67, 3.60) | 3.45 (2.87, 4.14) | 4.27 (2.92, 6.24) | 2.60 (2.16, 3.14) |
| 14 | 9.74 (8.93, 10.64) | 9.40 (8.08, 10.93) | 12.69 (9.17, 17.56) | 5.06 (3.77, 6.79) |
| 18C | 3.19 (2.72, 3.74) | 2.86 (2.38, 3.44) | 2.44 (1.73, 3.44) | 2.89 (2.46, 3.39) |
| 19F | 7.67 (6.92, 8.50) | 8.22 (6.85, 9.86) | 15.99 (10.73, 23.82) | 4.90 (4.60, 5.23) |
| 23F | 4.89 (4.20, 5.68) | 3.76 (2.89, 4.90) | 2.76 (1.96, 3.89) | 2.44 (1.95, 3.06) |
| 1 | 3.82 (3.32, 4.40) | 4.08 (3.18, 5.23) | 11.51 (9.23, 14.36) | 3.52 (2.58, 4.80) |
| 3 | 0.96 (0.86, 1.07) | 0.89 (0.74, 1.06) | 1.10 (0.69, 1.76) | 1.39 (1.08, 1.79) |
| 5 | 3.24 (2.84, 3.70) | 2.76 (2.25, 3.39) | 3.26 (2.11, 5.03) | 1.76 (1.30, 2.37) |
| 6A | 8.74 (7.89, 9.68) | 8.39 (7.03, 10.01) | 8.67 (6.22, 12.06) | 2.39 (1.55, 3.69) |
| 7F | 5.76 (5.21, 6.36) | 4.76 (4.06, 5.56) | 4.46 (3.79, 5.26) | 3.64 (3.06, 4.34) |
| 19A | 8.64 (7.85, 9.50) | 7.44 (6.42, 8.62) | 10.20 (8.44, 12.32) | 5.17 (3.97, 6.72) |
| PCV15 | | | | |
| 4 | 1.68 (1.30, 2.17) | 1.35 (1.24, 1.47) | - | - |
| 6B | 6.24 (5.34, 7.29) | 4.36 (4.00, 4.76) | - | - |
| 9V | 2.98 (2.39, 3.72) | 2.14 (2.05, 2.23) | - | - |
| 14 | 7.51 (6.13, 9.19) | 5.30 (5.02, 5.60) | - | - |
| 18C | 3.01 (2.64, 3.42) | 2.02 (1.87, 2.18) | - | - |
| 19F | 4.84 (4.05, 5.79) | 4.08 (3.90, 4.28) | - | - |
| 23F | 2.57 (2.19, 3.02) | 1.55 (1.47, 1.63) | - | - |
| 1 | 1.88 (1.48, 2.39) | 1.29 (1.23, 1.34) | - | - |
| 3 | 1.14 (0.96, 1.36) | 0.84 (0.80, 0.89) | - | - |
| 5 | 3.42 (2.64, 4.43) | 2.02 (1.92, 2.13) | - | - |
| 6A | 4.66 (4.10, 5.29) | 3.16 (2.98, 3.34) | - | - |

| Serotype | IgG GMCs (95%CI) by vaccine schedule (μg/mL) | | | |
|---|---|---|---|---|
| | 3+1 | 2+1 | 1+1 | 3+0 |
| 7F | 4.03 (3.57, 4.55) | 2.93 (2.64, 3.25) | - | - |
| 19A | 5.28 (4.43, 6.30) | 4.71 (4.48, 4.95) | - | - |
| 22F | 8.90 (7.25, 10.93) | 6.02 (5.75, 6.30) | - | - |
| 33F | 5.01 (4.26, 5.89) | 3.35 (3.19, 3.51) | - | - |
| PCV20† | | | | |
| 4 | 5.80 (3.78, 8.90) | 4.11 (3.77, 4.48) | - | - |
| 6B | 5.74 (3.97, 8.31) | 2.64 (2.36, 2.95) | - | - |
| 9V | 4.65 (3.44, 6.30) | 3.68 (3.42, 3.97) | - | - |
| 14 | 7.62 (5.65, 10.27) | 4.52 (4.08, 5.00) | - | - |
| 18C | 4.17 (3.14, 5.55) | 2.71 (2.52, 2.93) | - | - |
| 19F | 6.91 (4.99, 9.58) | 6.19 (5.68, 6.75) | - | - |
| 23F | 5.59 (3.89, 8.06) | 2.64 (2.40, 2.91) | - | - |
| 1 | 2.21 (1.47, 3.30) | 1.71 (1.58, 1.84) | - | - |
| 3 | 0.88 (0.56, 1.39) | 0.72 (0.67, 0.78) | - | - |
| 5 | 2.64 (1.85, 3.77) | 1.74 (1.60, 1.89) | - | - |
| 6A | 11.95 (9.01, 15.85) | 7.75 (7.04, 8.53) | - | - |
| 7F | 4.88 (3.78, 6.30) | 3.61 (3.40, 3.84) | - | - |
| 19A | 5.41 (3.41, 8.59) | 4.51 (4.11, 4.94) | - | - |
| 22F | 12.92 (10.52, 15.88) | 9.27 (8.52, 10.08) | - | - |
| 33F | 7.87 (4.71, 13.17) | 6.37 (5.83, 6.95) | - | - |
| 8 | 4.17 (2.91, 5.98) | 3.57 (3.32, 3.83) | - | - |
| 10A | 7.87 (6.03, 10.26) | 4.86 (4.41, 5.36) | - | - |
| 11A | 4.86 (3.53, 6.69) | 3.74 (3.44, 4.07) | - | - |
| 12F | 2.12 (1.68, 2.67) | 1.86 (1.71, 2.01) | - | - |
| 15B | 17.13 (12.44, 23.60) | 13.09 (12.10, 14.15) | - | - |
| PCV10-SII‡ | | | | |
| 6B | 10.01 (6.63, 15.13) | 12.46 (11.07, 14.01) | - | - |
| 9V | 2.06 (1.53, 2.76) | 3.46 (3.08, 3.88) | - | - |
| 14 | 6.98 (6.25, 7.81) | 8.28 (6.97, 9.82) | - | - |
| 19F | 6.58 (5.40, 8.00) | 11.11 (9.70, 12.73) | - | - |
| 23F | 4.35 (3.61, 5.25) | 4.95 (4.28, 5.73) | - | - |
| 1 | 5.74 (5.29, 6.22) | 8.45 (7.54, 9.48) | - | - |
| 5 | 1.72 (0.99, 2.98) | 1.54 (1.38, 1.73) | - | - |
| 6A | 6.63 (3.45, 12.77) | 9.56 (8.26, 11.05) | - | - |
| 7F | 6.40 (5.93, 6.90) | 6.66 (5.96, 7.44) | - | - |
| 19A | 5.00 (3.00, 8.34) | 8.82 (7.65, 10.15) | - | - |

†: Only 1 study arm available for PCV20 (2+1 schedule), no pooled estimate or heterogeneity.
‡: Only 1 study arm available for PCV10-SII (2+1 schedule), no pooled estimate or heterogeneity.
Heterogeneity for PCV7 (3+1): $I^2$=98.7%, $\tau^2$=0.361, p <0.01; for PCV7 (2+1): $I^2$=97.6%, $\tau^2$=0.295, p <0.01; for PCV7 (3+0): $I^2$96.7%, $\tau^2$=0.417, p <0.01; for PCV13 (3+1): $I^2$=99.4%, $\tau^2$=0.576, p <0.01; for PCV13 (2+1): $I^2$=99.3%, $\tau^2$=0.538, p <0.01; for PCV13 (1+1): $I^2$=98.6%, $\tau^2$=0.736, p <0.01; for PCV13 (3+0): $I^2$=97.8%, $\tau^2$=0.229, p <0.01; for PCV15 (3+1): $I^2$=99.2%, $\tau^2$=0.350, p <0.01; for PCV15 (2+1): $I^2$=99.6%, $\tau^2$=0.329, p <0.01; for PCV20 (3+1): $I^2$=99.6%, $\tau^2$=0.509, p <0.01; for PCV10-SII (3+1): $I^2$=99.1%, $\tau^2$=0.329, p <0.01.

## Supplementary Table 27. Summary of the meta-analysis results of anti-pneumococcal IgG seroresponse rates (%) post-childhood-schedule by vaccine product and schedule

| Serotype | Seroresponse rate (95%CI) by vaccine schedule (%) | | | |
|---|---|---|---|---|
| | 3+1 | 2+1 | 1+1 | 3+0 |
| PCV7 | | | | |
| 4 | 98% (97%-99%) | 100% (98%-100%) | - | 98% (94%-99%) |
| 6B | 99% (98%-99%) | 97% (86%-99%) | - | 79% (17%-98%) |
| 9V | 99% (98%-100%) | 98% (95%-99%) | - | 94% (88%-98%) |
| 14 | 99% (98%-100%) | 99% (97%-100%) | - | 96% (92%-98%) |
| 18C | 99% (98%-99%) | 100% (98%-100%) | - | 98% (95%-99%) |
| 19F | 99% (98%-99%) | 98% (96%-99%) | - | 95% (91%-98%) |
| 23F | 99% (98%-99%) | 98% (97%-99%) | - | 92% (70%-98%) |
| PCV13 | | | | |
| 4 | 99% (98%-99%) | 98% (97%-99%) | 100% (99%-100%) | 98% (96%-99%) |
| 6B | 99% (99%-100%) | 98% (97%-99%) | 96% (93%-98%) | 90% (82%-94%) |
| 9V | 99% (98%-99%) | 98% (97%-99%) | 98% (96%-99%) | 97% (95%-98%) |
| 14 | 99% (99%-99%) | 99% (98%-99%) | 98% (94%-99%) | 98% (97%-99%) |
| 18C | 99% (98%-99%) | 98% (97%-99%) | 98% (96%-99%) | 97% (94%-98%) |
| 19F | 99% (98%-99%) | 99% (98%-99%) | 98% (96%-99%) | 98% (97%-98%) |
| 23F | 99% (98%-99%) | 97% (96%-98%) | 96% (94%-98%) | 93% (88%-96%) |
| 1 | 99% (98%-99%) | 99% (98%-99%) | 99% (98%-100%) | 97% (93%-99%) |
| 3 | 92% (87%-95%) | 89% (83%-93%) | 90% (79%-95%) | 97% (88%-99%) |
| 5 | 99% (98%-99%) | 98% (97%-99%) | 99% (97%-100%) | 94% (91%-96%) |
| 6A | 99% (99%-100%) | 99% (98%-99%) | 97% (95%-99%) | 96% (91%-98%) |

| Serotype | Seroresponse rate (95%CI) by vaccine schedule (%) | | | |
|---|---|---|---|---|
| | 3+1 | 2+1 | 1+1 | 3+0 |
| 7F | 99% (99%-100%) | 99% (99%-100%) | 99% (98%-100%) | 99% (96%-100%) |
| 19A | 100% (99%-100%) | 99% (98%-100%) | 100% (99%-100%) | 99% (99%-100%) |
| PCV15* | | | | |
| 4 | 100% (92%-100%) | 96% (95%-97%) | - | - |
| 6B | 100% (92%-100%) | 98% (95%-100%) | - | - |
| 9V | 100% (92%-100%) | 99% (97%-100%) | - | - |
| 14 | 100% (92%-100%) | 100% (98%-100%) | - | - |
| 18C | 100% (92%-100%) | 99% (97%-100%) | - | - |
| 19F | 100% (92%-100%) | 100% (99%-100%) | - | - |
| 23F | 100% (92%-100%) | 97% (96%-98%) | - | - |
| 1 | 100% (92%-100%) | 97% (95%-98%) | - | - |
| 3 | 98% (87%-100%) | 92% (91%-94%) | - | - |
| 5 | 100% (92%-100%) | 99% (98%-100%) | - | - |
| 6A | 100% (92%-100%) | 99% (98%-99%) | - | - |
| 7F | 100% (92%-100%) | 100% (99%-100%) | - | - |
| 19A | 100% (92%-100%) | 99% (98%-100%) | - | - |
| 22F | 100% (92%-100%) | 100% (99%-100%) | - | - |
| 33F | 100% (92%-100%) | 99% (98%-100%) | - | - |
| PCV20† | | | | |
| 4 | 97% (79%-100%) | 99% (98%-100%) | - | - |
| 6B | 99% (98%-100%) | 98% (97%-99%) | - | - |
| 9V | 99% (93%-100%) | 99% (98%-100%) | - | - |
| 14 | 99% (98%-99%) | 97% (95%-98%) | - | - |
| 18C | 99% (98%-99%) | 99% (98%-100%) | - | - |
| 19F | 99% (97%-100%) | 100% (99%-100%) | - | - |
| 23F | 98% (92%-100%) | 96% (94%-98%) | - | - |
| 1 | 97% (88%-99%) | 97% (95%-98%) | - | - |
| 3 | 84% (59%-95%) | 83% (79%-86%) | - | - |
| 5 | 99% (95%-100%) | 98% (97%-99%) | - | - |
| 6A | 100% (99%-100%) | 99% (97%-100%) | - | - |
| 7F | 100% (99%-100%) | 100% (99%-100%) | - | - |
| 19A | 100% (99%-100%) | 100% (99%-100%) | - | - |
| 22F | 100% (99%-100%) | 99% (98%-100%) | - | - |
| 33F | 100% (99%-100%) | 99% (97%-99%) | - | - |
| 8 | 100% (99%-100%) | 99% (98%-100%) | - | - |
| 10A | 99% (94%-100%) | 98% (96%-99%) | - | - |
| 11A | 99% (97%-100%) | 98% (97%-99%) | - | - |
| 12F | 97% (91%-99%) | 97% (95%-98%) | - | - |
| 15B | 100% (99%-100%) | 99% (98%-100%) | - | - |
| PCV10-SII‡ | | | | |
| 6B | - | 100% (98%-100%) | - | - |
| 9V | - | 99% (96%-100%) | - | - |
| 14 | - | 98% (96%-100%) | - | - |
| 19F | - | 100% (98%-100%) | - | - |
| 23F | - | 98% (95%-99%) | - | - |
| 1 | - | 100% (98%-100%) | - | - |
| 5 | - | 98% (94%-99%) | - | - |
| 6A | - | 98% (95%-99%) | - | - |
| 7F | - | 100% (98%-100%) | - | - |
| 19A | - | 100% (98%-100%) | - | - |

*: Only 1 study arm available for PCV15 (3+1 schedule), no pooled estimate or heterogeneity.
†: Only 1 study arm available for PCV20 (2+1 schedule), no pooled estimate or heterogeneity.
‡: Only 1 study arm available for PCV10-SII (2+1 schedule), no pooled estimate or heterogeneity.
Heterogeneity for PCV7 (3+1): $I^2$=61.5%, $\tau^2$=0.732, p <0.01; for PCV7 (2+1): $I^2$=35.9%, $\tau^2$=0.192, p <0.01; for PCV7 (3+0): $I^2$=87.1%, $\tau^2$=1.284, p <0.01; for PCV13 (3+1): $I^2$=85.0%, $\tau^2$=0.916, p <0.01; for PCV13 (2+1): $I^2$=83.2%, $\tau^2$=0.858, p <0.01; for PCV13 (1+1): $I^2$=63.3%, $\tau^2$=0.755, p <0.01; for PCV13 (3+0): $I^2$=90.3%, $\tau^2$=0.952, p <0.01; for PCV15 (2+1): $I^2$=87.0%, $\tau^2$=0.939, p <0.01; for PCV20 (3+1): $I^2$=95.6%, $\tau^2$=1.286, p <0.01.

### Supplementary Table 28. Summary of the meta-analysis results of anti-pneumococcal IgG geometric mean concentrations (IgG GMCs, µg/mL) post "3+1" vaccination schedule by vaccine product and region

| Serotype | IgG GMCs (95%CI) by WHO region (µg/mL) | | | | | |
|---|---|---|---|---|---|---|
| | Africa | Americas | Europe | South East Asia | Western Pacific | Multi-regions |
| PCV7† | | | | | | |
| 4 | - | 2.29 (1.69, 3.11) | 4.25 (3.78, 4.78) | 5.76 (5.35, 6.20) | 8.91 (6.03, 13.16) | 2.80 (1.90, 4.00) |
| 6B | - | 9.27 (7.62, 11.26) | 7.27 (5.89, 8.97) | 10.73 (9.16, 12.57) | 15.52 (12.16, 19.82) | 7.00 (4.50, 10.90) |
| 9V | - | 3.19 | 3.41 | 3.89 | 5.38 | 5.90 |

| Serotype | IgG GMCs (95%CI) by WHO region (µg/mL) | | | | | |
|---|---|---|---|---|---|---|
| | Africa | Americas | Europe | South East Asia | Western Pacific | Multi-regions |
| | | (2.92, 3.49) | (2.81, 4.13) | (2.86, 5.29) | (4.25, 6.82) | (3.60, 9.90) |
| 14 | - | 7.87 (6.67, 9.29) | 11.60 (10.12, 13.28) | 12.24 (9.87, 15.16) | 17.48 (14.80, 20.64) | 10.80 (6.10, 18.80) |
| 18C | - | 2.89 (2.44, 3.42) | 2.74 (2.35, 3.18) | 3.01 (2.80, 3.23) | 6.02 (4.67, 7.77) | 3.90 (2.70, 5.80) |
| 19F | - | 3.86 (3.49, 4.26) | 4.39 (3.87, 4.98) | 5.72 (5.28, 6.20) | 9.12 (6.66, 12.48) | 4.20 (2.80, 6.10) |
| 23F | - | 4.84 (4.21, 5.56) | 4.80 (4.08, 5.64) | 6.21 (4.54, 8.51) | 11.27 (9.57, 13.27) | 7.10 (4.60, 10.90) |
| PCV13 | | | | | | |
| 4 | 4.54 (3.61, 5.70) | 2.84 (2.04, 3.96) | 3.76 (3.18, 4.45) | 7.71 (5.05, 11.79) | 7.08 (4.21, 11.89) | 2.08 (1.70, 2.55) |
| 6B | 13.97 (10.43, 18.72) | 8.41 (6.83, 10.35) | 8.60 (7.37, 10.04) | 12.04 (11.02, 13.15) | 12.93 (11.19, 14.96) | 6.87 (6.22, 7.60) |
| 9V | 3.89 (3.05, 4.96) | 2.52 (1.89, 3.36) | 2.60 (2.18, 3.11) | 5.23 (2.78, 9.84) | 5.28 (3.97, 7.02) | 3.01 (2.45, 3.70) |
| 14 | 9.44 (6.74, 13.22) | 7.98 (7.16, 8.91) | 10.10 (9.43, 10.81) | 11.19 (10.16, 12.33) | 15.53 (10.60, 22.76) | 9.44 (7.95, 11.21) |
| 18C | 5.75 (4.37, 7.56) | 2.84 (2.12, 3.79) | 2.46 (1.96, 3.10) | 4.04 (2.48, 6.58) | 6.70 (4.53, 9.91) | 2.93 (2.59, 3.32) |
| 19F | 10.42 (7.79, 13.93) | 6.50 (5.65, 7.48) | 7.78 (6.66, 9.08) | 10.74 (8.07, 14.29) | 11.32 (8.05, 15.93) | 6.29 (5.10, 7.77) |
| 23F | 8.85 (5.10, 15.33) | 4.70 (3.74, 5.91) | 3.74 (3.27, 4.29) | 6.62 (4.00, 10.96) | 10.33 (7.56, 14.10) | 3.26 (2.67, 4.00) |
| 1 | 6.06 (4.97, 7.40) | 2.99 (2.46, 3.63) | 4.02 (3.42, 4.72) | 4.70 (4.27, 5.17) | 9.32 (7.51, 11.56) | 2.60 (2.22, 3.04) |
| 3 | 2.45 (1.98, 3.03) | 0.89 (0.75, 1.06) | 0.90 (0.75, 1.09) | 1.21 (0.88, 1.66) | 1.57 (1.39, 1.77) | 0.70 (0.66, 0.75) |
| 5 | 2.64 (2.26, 3.08) | 2.80 (2.21, 3.54) | 3.03 (2.41, 3.80) | 4.37 (3.45, 5.54) | 6.12 (4.11, 9.12) | 2.89 (2.28, 3.66) |
| 6A | 11.42 (8.05, 16.21) | 8.88 (7.35, 10.73) | 6.96 (6.30, 7.68) | 12.48 (7.59, 20.51) | 11.08 (8.60, 14.27) | 7.55 (6.63, 8.60) |
| 7F | 9.32 (7.85, 11.06) | 5.04 (4.23, 6.01) | 5.15 (4.57, 5.80) | 6.23 (4.63, 8.37) | 9.06 (6.83, 12.03) | 5.28 (4.95, 5.63) |
| 19A | 11.70 (8.04, 17.02) | 6.78 (5.97, 7.71) | 9.77 (8.81, 10.85) | 13.46 (12.43, 14.57) | 12.13 (9.92, 14.84) | 7.28 (6.27, 8.46) |
| PCV15 | | | | | | |
| 4 | - | - | - | - | 2.41 (1.46, 3.98) | 1.43 (1.25, 1.63) |
| 6B | - | - | - | - | 7.47 (6.27, 8.90) | 5.84 (4.89, 6.98) |
| 9V | - | - | - | - | 4.12 (2.64, 6.43) | 2.58 (2.37, 2.81) |
| 14 | - | - | - | - | 10.22 (7.93, 13.16) | 6.55 (5.83, 7.37) |
| 18C | - | - | - | - | 3.79 (3.21, 4.48) | 2.79 (2.56, 3.03) |
| 19F | - | - | - | - | 6.04 (4.90, 7.43) | 4.41 (3.69, 5.27) |
| 23F | - | - | - | - | 3.08 (2.59, 3.67) | 2.41 (1.99, 2.93) |
| 1 | - | - | - | - | 2.64 (1.93, 3.62) | 1.62 (1.36, 1.93) |
| 3 | - | - | - | - | 1.48 (1.19, 1.84) | 1.03 (0.91, 1.16) |
| 5 | - | - | - | - | 4.61 (2.35, 9.06) | 2.98 (2.59, 3.44) |
| 6A | - | - | - | - | 5.65 (4.73, 6.76) | 4.38 (3.86, 4.97) |
| 7F | - | - | - | - | 4.56 (3.70, 5.61) | 3.87 (3.37, 4.44) |
| 19A | - | - | - | - | 6.22 (3.90, 9.91) | 4.93 (4.23, 5.74) |
| 22F | - | - | - | - | 11.20 (7.67, 16.35) | 7.84 (7.20, 8.54) |
| 33F | - | - | - | - | 6.22 (5.34, 7.25) | 4.49 (4.02, 5.01) |
| PCV20[ǂ] | | | | | | |
| 4 | - | 5.18 (2.76, 9.71) | - | - | 7.31 (6.51, 8.20) | - |
| 6B | - | 5.03 (3.19, 7.91) | - | - | 7.50 (6.58, 8.55) | - |
| 9V | - | 4.34 | - | - | 5.38 | - |

| Serotype | IgG GMCs (95%CI) by WHO region (μg/mL) | | | | | |
|---|---|---|---|---|---|---|
| | Africa | Americas | Europe | South East Asia | Western Pacific | Multi-regions |
| | | (2.73, 6.90) | | | (4.81, 6.02) | |
| 14 | - | 6.95 (4.62, 10.44) | - | - | 9.19 (8.10, 10.43) | - |
| 18C | - | 4.38 (2.74, 6.99) | - | - | 3.81 (3.38, 4.30) | - |
| 19F | - | 6.22 (4.03, 9.58) | - | - | 8.56 (7.66, 9.56) | - |
| 23F | - | 4.87 (3.20, 7.40) | - | - | 7.39 (6.49, 8.42) | - |
| 1 | - | 1.97 (1.10, 3.51) | - | - | 2.78 (2.47, 3.12) | - |
| 3 | - | 0.80 (0.39, 1.62) | - | - | 1.08 (0.96, 1.21) | - |
| 5 | - | 2.52 (1.40, 4.53) | - | - | 2.94 (2.59, 3.33) | - |
| 6A | - | 11.10 (7.33, 16.82) | - | - | 13.92 (12.43, 15.59) | - |
| 7F | - | 4.90 (3.15, 7.62) | - | - | 4.85 (4.34, 5.42) | - |
| 19A | - | 4.47 (2.79, 7.16) | - | - | 7.92 (7.06, 8.89) | - |
| 22F | - | 12.38 (9.00, 17.03) | - | - | 14.21 (12.61, 16.00) | - |
| 33F | - | 6.63 (3.39, 12.95) | - | - | 11.13 (9.99, 12.39) | - |
| 8 | - | 3.54 (2.79, 4.48) | - | - | 5.88 (5.23, 6.62) | - |
| 10A | - | 7.83 (4.95, 12.38) | - | - | 8.02 (7.02, 9.16) | - |
| 11A | - | 4.47 (2.79, 7.15) | - | - | 5.78 (5.14, 6.50) | - |
| 12F | - | 1.87 (1.75, 1.99) | - | - | 2.69 (2.36, 3.06) | - |
| 15B | - | 15.19 (10.45, 22.10) | - | - | 21.83 (19.53, 24.41) | - |
| PCV10-SII | | | | | | |
| 6B | 10.01 (6.63, 15.13) | - | - | - | - | - |
| 9V | 2.06 (1.53, 2.76) | - | - | - | - | - |
| 14 | 6.98 (6.25, 7.81) | - | - | - | - | - |
| 19F | 6.58 (5.40, 8.00) | - | - | - | - | - |
| 23F | 4.35 (3.61, 5.25) | - | - | - | - | - |
| 1 | 5.74 (5.29, 6.22) | - | - | - | - | - |
| 5 | 1.72 (0.99, 2.98) | - | - | - | - | - |
| 6A | 6.63 (3.45, 12.77) | - | - | - | - | - |
| 7F | 6.40 (5.93, 6.90) | - | - | - | - | - |
| 19A | 5.00 (3.00, 8.34) | - | - | - | - | - |

†: Only 1 study arm available for PCV7 (Multi-regions), no pooled estimate or heterogeneity.
‡: Only 1 study arm available for PCV20 (Western Pacific Region), no pooled estimate or heterogeneity.
Heterogeneity for PCV7 (Americas): $I^2$=98.5%, $\tau^2$=0.303, p <0.01; PCV7 (Europe): $I^2$=98.3%, $\tau^2$=0.274, p <0.01; PCV7 (South-East Asia): $I^2$=98.1%, $\tau^2$=0.241, p <0.01; PCV7 (Western Pacific): $I^2$=98.5%, $\tau^2$=0.272, p <0.01; for PCV13 (Africa): $I^2$=94.2%, $\tau^2$=0.309, p <0.01; for PCV13 (Americas): $I^2$=99.4%, $\tau^2$=0.580, p <0.01; PCV13 (Europe): $I^2$=99.3%, $\tau^2$=0.496, p <0.01; PCV13 (South-East Asia): $I^2$=99.0%, $\tau^2$=0.508, p <0.01; PCV13 (Western Pacific): $I^2$=99.5%, $\tau^2$=0.424, p <0.01; PCV13 (Multi-region): $I^2$=99.4%, $\tau^2$=0.515, p <0.01; for PCV15(Western Pacific): $I^2$=95.9%, $\tau^2$=0.306, p <0.01; PCV15 (Multi-region): $I^2$=99.4%, $\tau^2$=0.334, p <0.01; for PCV20 (Americas): $I^2$=99.6%, $\tau^2$=0.514, p <0.01; for PCV10-SII (Africa): $I^2$=99.1%, $\tau^2$=0.329, p <0.01.

**Supplementary Table 29. Summary of the meta-analysis results of anti-pneumococcal IgG seroresponse rates (%) post "3+1" vaccination schedule by vaccine product and region**

| Serotype | IgG GMCs (95%CI) by WHO region (μg/mL)* | | | | | |
|---|---|---|---|---|---|---|
| | Africa | Americas | Europe | South East Asia | Western Pacific | Multi-regions |
| PCV7* | | | | | | |
| 4 | - | 96% (94%-98%) | 99% (99%-100%) | 100% (96%-100%) | 100% (99%-100%) | 85% (65%-97%) |
| 6B | - | 99% (98%-100%) | 98% (97%-99%) | 99% (98%-100%) | 100% (99%-100%) | 77% (55%-92%) |
| 9V | - | 99% (98%-100%) | 99% (98%-100%) | 99% (98%-100%) | 100% (99%-100%) | 85% (65%-97%) |
| 14 | - | 99% (98%-100%) | 99% (99%-100%) | 99% (98%-100%) | 100% (98%-100%) | 85% (65%-97%) |
| 18C | - | 98% (97%-99%) | 99% (97%-99%) | 99% (97%-100%) | 100% (99%-100%) | 85% (65%-97%) |
| 19F | - | 99% (98%-99%) | 99% (97%-99%) | 98% (96%-99%) | 100% (99%-100%) | 85% (65%-97%) |
| 23F | - | 99% (98%-99%) | 99% (98%-99%) | 99% (98%-100%) | 99% (95%-100%) | 85% (65%-97%) |
| PCV13† | | | | | | |
| 4 | 100% (95%-100%) | 98% (95%-99%) | 99% (99%-100%) | 100% (98%-100%) | 100% (99%-100%) | 98% (96%-99%) |
| 6B | 99% (92%-100%) | 100% (99%-100%) | 99% (99%-100%) | 98% (95%-99%) | 100% (99%-100%) | 99% (98%-100%) |
| 9V | 99% (92%-100%) | 98% (97%-99%) | 99% (99%-100%) | 99% (98%-100%) | 100% (98%-100%) | 99% (96%-100%) |
| 14 | 99% (92%-100%) | 100% (99%-100%) | 100% (99%-100%) | 98% (96%-99%) | 100% (99%-100%) | 100% (98%-100%) |
| 18C | 97% (90%-100%) | 99% (98%-99%) | 98% (97%-99%) | 99% (97%-100%) | 100% (99%-100%) | 99% (98%-100%) |
| 19F | 94% (86%-98%) | 99% (98%-99%) | 98% (97%-99%) | 98% (97%-99%) | 100% (99%-100%) | 100% (99%-100%) |
| 23F | 98% (91%-100%) | 98% (98%-99%) | 99% (98%-99%) | 98% (97%-99%) | 98% (97%-99%) | 99% (98%-99%) |
| 1 | 97% (90%-100%) | 97% (96%-98%) | 99% (98%-100%) | 99% (97%-99%) | 99% (99%-100%) | 99% (97%-100%) |
| 3 | 99% (92%-100%) | 87% (76%-93%) | 88% (82%-93%) | 95% (88%-98%) | 99% (98%-99%) | 80% (77%-83%) |
| 5 | 97% (90%-100%) | 98% (97%-99%) | 99% (98%-99%) | 100% (98%-100%) | 100% (99%-100%) | 98% (94%-100%) |
| 6A | 96% (88%-99%) | 100% (99%-100%) | 99% (98%-100%) | 98% (91%-99%) | 100% (99%-100%) | 100% (98%-100%) |
| 7F | 99% (92%-100%) | 100% (99%-100%) | 99% (98%-100%) | 99% (98%-100%) | 99% (98%-100%) | 100% (98%-100%) |
| 19A | 98% (92%-100%) | 100% (99%-100%) | 100% (99%-100%) | 99% (98%-100%) | 100% (99%-100%) | 100% (99%-100%) |
| PCV15ǂ | | | | | | |
| 4 | - | - | - | - | 100% (92%-100%) | - |
| 6B | - | - | - | - | 100% (92%-100%) | - |
| 9V | - | - | - | - | 100% (92%-100%) | - |
| 14 | - | - | - | - | 100% (92%-100%) | - |
| 18C | - | - | - | - | 100% (92%-100%) | - |
| 19F | - | - | - | - | 100% (92%-100%) | - |
| 23F | - | - | - | - | 100% (92%-100%) | - |
| 1 | - | - | - | - | 100% (92%-100%) | - |
| 3 | - | - | - | - | 98% (87%-100%) | - |
| 5 | - | - | - | - | 100% (92%-100%) | - |
| 6A | - | - | - | - | 100% (92%-100%) | - |
| 7F | - | - | - | - | 100% (92%-100%) | - |
| 19A | - | - | - | - | 100% (92%-100%) | - |
| 22F | - | - | - | - | 100% | - |

| Serotype | IgG GMCs (95%CI) by WHO region (μg/mL)* | | | | | |
|---|---|---|---|---|---|---|
| | Africa | Americas | Europe | South East Asia | Western Pacific | Multi-regions |
| | | | | | (92%-100%) | |
| 33F | - | - | - | - | 100% (92%-100%) | - |
| PCV20# | | | | | | |
| 4 | - | 99% (98%-100%) | - | - | 92% (87%-95%) | - |
| 6B | - | 99% (98%-100%) | - | - | 100% (98%-100%) | - |
| 9V | - | 98% (97%-99%) | - | - | 100% (98%-100%) | - |
| 14 | - | 99% (98%-100%) | - | - | 100% (98%-100%) | - |
| 18C | - | 99% (98%-100%) | - | - | 100% (98%-100%) | - |
| 19F | - | 99% (98%-99%) | - | - | 100% (98%-100%) | - |
| 23F | - | 97% (96%-98%) | - | - | 100% (97%-100%) | - |
| 1 | - | 94% (92%-96%) | - | - | 99% (96%-100%) | - |
| 3 | - | 74% (70%-77%) | - | - | 92% (87%-95%) | - |
| 5 | - | 98% (97%-99%) | - | - | 100% (97%-100%) | - |
| 6A | - | 100% (99%-100%) | - | - | 100% (98%-100%) | - |
| 7F | - | 100% (99%-100%) | - | - | 100% (98%-100%) | - |
| 19A | - | 100% (99%-100%) | - | - | 100% (98%-100%) | - |
| 22F | - | 100% (99%-100%) | - | - | 100% (98%-100%) | - |
| 33F | - | 100% (99%-100%) | - | - | 100% (98%-100%) | - |
| 8 | - | 100% (99%-100%) | - | - | 100% (98%-100%) | - |
| 10A | - | 98% (96%-99%) | - | - | 100% (97%-100%) | - |
| 11A | - | 99% (98%-99%) | - | - | 100% (98%-100%) | - |
| 12F | - | 95% (93%-97%) | - | - | 99% (96%-100%) | - |
| 15B | - | 100% (99%-100%) | - | - | 100% (98%-100%) | - |

*No Seroresponse rate data were reported for PCV10-SII study.
*: Only 1 study arm available for PCV7 (Multi-regions), no pooled estimate or heterogeneity.
†: Only 1 study arm available for PCV13 (African Region), no pooled estimate or heterogeneity.
‡: Only 1 study arm available for PCV15 (Western Pacific Region), no pooled estimate or heterogeneity.
#: Only 1 study arm available for PCV20 (Western Pacific Region) and PCV20 (Americas), no pooled estimate or heterogeneity.
Heterogeneity for PCV7 (Americas): $I^2$=57.2%, $\tau^2$=0.292, p <0.01; PCV7 (Europe): $I^2$=0%, $\tau^2$=0, p = 0.63; PCV7 (South-East Asia): $I^2$=0, $\tau^2$=0, p = 0.73; PCV7 (Western Pacific): $I^2$=21.8%, $\tau^2$=0.102, p = 0.22; for PCV13 (Americas): $I^2$=89.1%, $\tau^2$=1.007, p <0.01; PCV13 (Europe): $I^2$=83.4%, $\tau^2$=0.963, p <0.01; PCV13 (South-East Asia): $I^2$=60.9%, $\tau^2$=0.333, p <0.01; PCV13 (Western Pacific): $I^2$=12.7%, $\tau^2$=0.084, p = 0.25; PCV13 (Multi-region): $I^2$=85.5%, $\tau^2$=1.435, p <0.01.

### Supplementary Table 30. Summary of the meta-analysis results of anti-pneumococcal IgG geometric mean concentrations (IgG GMCs, μg/mL) by vaccine product and timepoint

| Serotype | IgG GMC (95%CI) by vaccine schedule (μg/mL) | | | |
|---|---|---|---|---|
| | Post 1-primary dose | Post 2-primary dose | Post 3-primary dose | Post booster |
| PCV7 | | | | |
| 4 | 0.68 (0.45, 1.02) | 2.66 (1.47, 4.79) | 3.25 (2.64, 3.99) | 4.41 (3.68, 5.28) |
| 6B | 0.16 (0.13, 0.19) | 0.47 (0.33, 0.66) | 1.99 (1.59, 2.49) | 8.83 (7.66, 10.17) |
| 9V | 0.40 (0.31, 0.53) | 1.93 (1.26, 2.94) | 2.37 (2.00, 2.81) | 3.83 (3.44, 4.26) |
| 14 | 0.97 (0.85, 1.12) | 3.38 (2.57, 4.45) | 6.30 (5.24, 7.58) | 11.54 (10.38, 12.83) |
| 18C | 0.54 (0.38, 0.77) | 1.70 (1.12, 2.59) | 2.69 (2.32, 3.13) | 3.24 (2.87, 3.66) |
| 19F | 1.05 (0.82, 1.33) | 3.54 (2.59, 4.82) | 3.44 (2.91, 4.07) | 5.13 (4.55, 5.78) |
| 23F | 0.23 (0.18, 0.29) | 0.78 (0.55, 1.11) | 2.18 (1.91, 2.50) | 5.66 (4.98, 6.42) |

| Serotype | IgG GMC (95%CI) by vaccine schedule (μg/mL) | | | |
|---|---|---|---|---|
| | Post 1-primary dose | Post 2-primary dose | Post 3-primary dose | Post booster |
| PCV13 | | | | |
| 4 | 0.56 (0.32, 0.98) | 1.84 (1.44, 2.37) | 2.14 (1.82, 2.51) | 3.70 (3.19, 4.29) |
| 6B | 0.14 (0.09, 0.21) | 0.38 (0.29, 0.51) | 1.75 (1.45, 2.11) | 7.63 (6.82, 8.55) |
| 9V | 0.29 (0.22, 0.37) | 1.35 (1.08, 1.68) | 1.68 (1.49, 1.90) | 3.29 (2.93, 3.68) |
| 14 | 0.62 (0.56, 0.69) | 3.76 (2.98, 4.74) | 5.26 (4.48, 6.18) | 9.88 (9.15, 10.66) |
| 18C | 0.47 (0.36, 0.60) | 1.43 (1.20, 1.71) | 1.99 (1.77, 2.25) | 3.02 (2.68, 3.40) |
| 19F | 0.75 (0.61, 0.92) | 4.41 (3.73, 5.22) | 2.92 (2.59, 3.29) | 8.38 (7.57, 9.26) |
| 23F | 0.14 (0.09, 0.21) | 0.68 (0.54, 0.84) | 1.57 (1.38, 1.79) | 4.33 (3.81, 4.93) |
| 1 | 0.81 (0.57, 1.16) | 2.08 (1.68, 2.58) | 2.28 (1.98, 2.63) | 4.33 (3.78, 4.95) |
| 3 | 0.77 (0.65, 0.92) | 0.75 (0.60, 0.94) | 0.92 (0.81, 1.05) | 0.95 (0.86, 1.05) |
| 5 | 0.45 (0.38, 0.54) | 1.10 (0.90, 1.34) | 1.49 (1.31, 1.71) | 3.11 (2.79, 3.47) |
| 6A | 0.24 (0.14, 0.44) | 1.40 (1.17, 1.68) | 2.27 (1.96, 2.63) | 8.65 (7.94, 9.42) |
| 7F | 0.76 (0.52, 1.12) | 2.53 (2.13, 3.01) | 3.08 (2.78, 3.41) | 5.35 (4.93, 5.79) |
| 19A | 0.46 (0.36, 0.59) | 2.29 (1.88, 2.77) | 2.70 (2.35, 3.10) | 8.43 (7.81, 9.10) |
| PCV15 | | | | |
| 4 | - | 1.57 (1.28, 1.93) | 1.79 (1.10, 2.92) | 1.58 (1.29, 1.93) |
| 6B | - | 0.43 (0.39, 0.47) | 1.90 (1.66, 2.18) | 5.70 (4.83, 6.72) |
| 9V | - | 1.38 (1.10, 1.73) | 2.06 (1.47, 2.90) | 2.73 (2.25, 3.31) |
| 14 | - | 4.65 (3.24, 6.67) | 6.29 (4.53, 8.73) | 6.86 (5.70, 8.27) |
| 18C | - | 1.18 (1.12, 1.23) | 1.73 (1.34, 2.24) | 2.73 (2.31, 3.23) |
| 19F | - | 2.71 (2.56, 2.87) | 2.72 (1.98, 3.74) | 4.62 (4.02, 5.32) |
| 23F | - | 0.73 (0.68, 0.78) | 1.55 (1.26, 1.91) | 2.26 (1.84, 2.77) |
| 1 | - | 1.34 (1.26, 1.43) | 1.63 (1.08, 2.46) | 1.71 (1.38, 2.11) |
| 3 | - | 0.98 (0.79, 1.22) | 1.43 (0.89, 2.30) | 1.06 (0.90, 1.24) |
| 5 | - | 1.01 (0.79, 1.28) | 1.92 (1.41, 2.61) | 2.99 (2.32, 3.86) |
| 6A | - | 0.66 (0.61, 0.70) | 1.73 (1.48, 2.03) | 4.24 (3.60, 4.98) |
| 7F | - | 1.86 (1.54, 2.23) | 2.57 (2.35, 2.81) | 3.71 (3.22, 4.27) |
| 19A | - | 1.71 (1.61, 1.81) | 2.07 (1.43, 3.01) | 5.11 (4.48, 5.84) |
| 22F | - | 2.96 (2.59, 3.38) | 5.51 (4.31, 7.05) | 7.94 (6.48, 9.72) |
| 33F | - | 0.30 (0.28, 0.33) | 1.78 (1.52, 2.08) | 4.47 (3.70, 5.40) |
| PCV20* | | | | |
| 4 | - | 0.55 (0.50, 0.61) | 1.20 (0.74, 1.96) | 5.32 (3.76, 7.52) |
| 6B | - | 0.03 (0.03, 0.04) | 0.53 (0.39, 0.72) | 4.73 (2.98, 7.52) |
| 9V | - | 0.45 (0.40, 0.51) | 1.15 (0.85, 1.55) | 4.38 (3.44, 5.59) |
| 14 | - | 1.05 (0.94, 1.18) | 2.53 (2.03, 3.14) | 6.69 (4.80, 9.33) |
| 18C | - | 0.69 (0.62, 0.77) | 1.46 (1.29, 1.64) | 3.74 (2.79, 5.02) |
| 19F | - | 2.21 (2.04, 2.40) | 2.05 (1.50, 2.81) | 6.71 (5.30, 8.51) |
| 23F | - | 0.13 (0.12, 0.15) | 1.00 (0.76, 1.30) | 4.64 (2.96, 7.27) |
| 1 | - | 0.57 (0.52, 0.62) | 0.92 (0.71, 1.20) | 2.07 (1.51, 2.82) |
| 3 | - | 0.41 (0.38, 0.45) | 0.55 (0.28, 1.09) | 0.84 (0.60, 1.17) |
| 5 | - | 0.34 (0.30, 0.38) | 0.84 (0.65, 1.09) | 2.38 (1.72, 3.29) |
| 6A | - | 0.45 (0.40, 0.52) | 1.93 (1.62, 2.32) | 10.73 (8.02, 14.36) |
| 7F | - | 1.02 (0.94, 1.10) | 1.77 (1.46, 2.16) | 4.52 (3.58, 5.71) |
| 19A | - | 0.67 (0.61, 0.74) | 1.13 (0.55, 2.34) | 5.17 (3.68, 7.25) |
| 22F | - | 2.25 (2.06, 2.45) | 4.19 (3.67, 4.78) | 11.89 (9.55, 14.82) |
| 33F | - | 0.31 (0.28, 0.34) | 1.74 (1.37, 2.19) | 7.47 (5.12, 10.90) |
| 8 | - | 1.62 (1.51, 1.74) | 2.31 (1.62, 3.29) | 4.01 (3.08, 5.23) |
| 10A | - | 0.16 (0.14, 0.18) | 0.98 (0.47, 2.07) | 6.98 (5.15, 9.45) |
| 11A | - | 1.62 (1.50, 1.75) | 2.41 (1.11, 5.26) | 4.55 (3.51, 5.90) |
| 12F | - | 0.15 (0.13, 0.17) | 0.69 (0.53, 0.90) | 2.04 (1.72, 2.44) |
| 15B | - | 3.33 (3.00, 3.70) | 5.57 (4.33, 7.17) | 16.00 (12.31, 20.80) |
| PCV10-SII‡ | | | | |
| 6B | - | 1.82 (1.48, 2.23) | 1.34 (1.05, 1.72) | 10.76 (8.16, 14.20) |
| 9V | - | 1.93 (1.74, 2.16) | 1.20 (0.99, 1.46) | 2.47 (1.70, 3.60) |
| 14 | - | 4.03 (3.50, 4.64) | 5.18 (4.91, 5.47) | 7.49 (6.58, 8.52) |
| 19F | - | 5.45 (4.94, 6.01) | 4.24 (3.85, 4.66) | 8.09 (5.73, 11.43) |
| 23F | - | 2.21 (1.92, 2.56) | 1.58 (1.51, 1.66) | 4.54 (3.96, 5.21) |
| 1 | - | 3.63 (3.32, 3.98) | 3.61 (2.54, 5.15) | 6.76 (5.28, 8.65) |
| 5 | - | 1.19 (1.10, 1.28) | 1.84 (1.46, 2.31) | 1.64 (1.19, 2.26) |
| 6A | - | 1.19 (1.00, 1.41) | 1.00 (0.95, 1.06) | 7.49 (4.79, 11.72) |
| 7F | - | 3.46 (3.09, 3.88) | 2.58 (1.92, 3.48) | 6.48 (6.09, 6.90) |
| 19A | - | 1.75 (1.57, 1.96) | 1.63 (1.56, 1.71) | 6.13 (3.82, 9.82) |

*: Only 1 study arm available for PCV20 (Post 2-primary), no pooled estimate or heterogeneity.
‡: Only 1 study arm available for PCV10-SII (Post 2-primary), no pooled estimate or heterogeneity.
Heterogeneity for PCV7 (post 1-primary): $I^2$=98.4%, $\tau^2$=0.476, p <0.01; for PCV7 (post 2-primary): $I^2$=99.0%, $\tau^2$=0.918, p <0.01; for PCV7 (post 3-primary): $I^2$=98.7%, $\tau^2$=0.410, p <0.01; for PCV7 (post booster): $I^2$=98.6%, $\tau^2$=0.348, p <0.01; for PCV13 (post 1-primary): $I^2$=98.1%, $\tau^2$=0.469, p <0.01; for PCV13 (post 2-primary): $I^2$=99.2%, $\tau^2$=0.660, p <0.01; for PCV13 (post 3-primary): $I^2$=99.2%, $\tau^2$=0.400, p <0.01; for PCV13 (post booster): $I^2$=99.3%, $\tau^2$=0.582, p <0.01; for PCV15 (post 2-primary): $I^2$=99.5%, $\tau^2$=0.511, p <0.01; for PCV15 (post 3-primary): $I^2$=98.9%, $\tau^2$=0.314, p <0.01; for PCV15 (post booster): $I^2$=99.4%, $\tau^2 = 0.361$, p <0.01; for PCV20 (post 3-primary): $I^2 = 99.4\%$, $\tau^2 = 0.462$, p <0.01; for PCV20 (post booster): $I^2$=99.6%,

$\tau^2$=0.508, p <0.01; for PCV10-SII (post 3-primary): $I^2$=99.7%, $\tau^2$=0.298, p <0.01; for PCV10-SII (post booster): $I^2$=99.1%, $\tau^2$=0.354, p <0.01.

**Supplementary Table 31. Summary of the meta-analysis results of anti-pneumococcal IgG seroresponse rates (%) by vaccine product and timepoint**

| Serotype | Seroresponse rate (95%CI) by vaccine schedule (%) | | | |
|---|---|---|---|---|
| | Post 1-primary dose | Post 2-primary dose | Post 3-primary dose | Post booster |
| PCV7 | | | | |
| 4 | 75% (58%-86%) | 96% (94%-97%) | 98% (98%-99%) | 99% (97%-99%) |
| 6B | 23% (19%-27%) | 61% (48%-72%) | 89% (85%-93%) | 99% (98%-99%) |
| 9V | 56% (49%-62%) | 94% (91%-96%) | 98% (97%-98%) | 99% (98%-100%) |
| 14 | 84% (73%-91%) | 96% (94%-97%) | 97% (96%-98%) | 99% (98%-100%) |
| 18C | 67% (54%-78%) | 93% (88%-96%) | 97% (97%-99%) | 99% (98%-99%) |
| 19F | 91% (81%-96%) | 96% (95%-98%) | 97% (96%-98%) | 99% (98%-99%) |
| 23F | 31% (20%-44%) | 77% (67%-84%) | 94% (92%-95%) | 99% (98%-99%) |
| PCV13 | | | | |
| 4 | 68% (46%-84%) | 96% (94%-97%) | 97% (96%-98%) | 99% (98%-99%) |
| 6B | 8% (4%-18%) | 55% (46%-64%) | 90% (87%-92%) | 99% (98%-99%) |
| 9V | 35% (27%-44%) | 92% (88%-94%) | 96% (95%-97%) | 99% (98%-99%) |
| 14 | 74% (70%-78%) | 96% (94%-97%) | 98% (97%-98%) | 99% (99%-99%) |
| 18C | 64% (51%-75%) | 91% (88%-93%) | 96% (96%-97%) | 99% (98%-99%) |
| 19F | 85% (77%-91%) | 98% (97%-99%) | 98% (97%-98%) | 99% (98%-99%) |
| 23F | 12% (8%-16%) | 72% (66%-77%) | 91% (90%-93%) | 98% (97%-98%) |
| 1 | 76% (70%-82%) | 96% (95%-97%) | 97% (96%-98%) | 99% (98%-99%) |
| 3 | 84% (78%-88%) | 87% (79%-92%) | 90% (87%-93%) | 90% (87%-93%) |
| 5 | 60% (54%-67%) | 90% (87%-93%) | 94% (92%-95%) | 99% (98%-99%) |
| 6A | 25% (10%-50%) | 88% (85%-91%) | 96% (94%-97%) | 99% (98%-99%) |
| 7F | 79% (67%-87%) | 98% (97%-99%) | 99% (98%-99%) | 99% (99%-99%) |
| 19A | 61% (50%-71%) | 96% (94%-97%) | 98% (98%-99%) | 99% (99%-100%) |
| PCV15 | | | | |
| 4 | - | 96% (90%-98%) | 97% (96%-98%) | 96% (95%-97%) |
| 6B | - | 58% (55%-61%) | 91% (89%-93%) | 98% (96%-99%) |
| 9V | - | 92% (85%-96%) | 97% (96%-98%) | 99% (98%-100%) |
| 14 | - | 97% (96%-98%) | 98% (97%-99%) | 99% (99%-100%) |
| 18C | - | 93% (91%-94%) | 97% (97%-98%) | 99% (98%-100%) |
| 19F | - | 98% (97%-99%) | 99% (98%-99%) | 100% (99%-100%) |
| 23F | - | 77% (74%-80%) | 93% (91%-95%) | 97% (96%-98%) |
| 1 | - | 96% (94%-98%) | 96% (95%-97%) | 97% (95%-98%) |
| 3 | - | 95% (91%-98%) | 95% (94%-96%) | 92% (91%-94%) |
| 5 | - | 89% (79%-94%) | 96% (95%-97%) | 99% (98%-100%) |
| 6A | - | 75% (71%-79%) | 95% (91%-98%) | 99% (98%-99%) |
| 7F | - | 98% (97%-99%) | 99% (98%-100%) | 100% (99%-100%) |
| 19A | - | 95% (93%-97%) | 98% (97%-99%) | 99% (99%-100%) |
| 22F | - | 97% (94%-98%) | 99% (98%-99%) | 100% (99%-100%) |
| 33F | - | 49% (46%-52%) | 91% (85%-95%) | 99% (98%-100%) |
| PCV20* | | | | |
| 4 | - | 69% (65%-72%) | 89% (76%-95%) | 98% (91%-100%) |
| 6B | - | 21% (17%-24%) | 86% (82%-89%) | 99% (98%-99%) |
| 9V | - | 60% (56%-64%) | 88% (80%-93%) | 99% (98%-99%) |
| 14 | - | 79% (75%-82%) | 94% (92%-96%) | 98% (94%-100%) |
| 18C | - | 71% (67%-75%) | 93% (91%-94%) | 99% (98%-99%) |
| 19F | - | 94% (92%-96%) | 98% (95%-99%) | 99% (98%-100%) |
| 23F | - | 24% (20%-27%) | 82% (74%-88%) | 98% (94%-99%) |
| 1 | - | 71% (67%-74%) | 87% (78%-92%) | 97% (93%-98%) |
| 3 | - | 58% (54%-62%) | 77% (37%-95%) | 84% (70%-92%) |
| 5 | - | 63% (59%-67%) | 88% (80%-93%) | 98% (97%-99%) |
| 6A | - | 60% (55%-64%) | 94% (92%-95%) | 99% (98%-100%) |
| 7F | - | 88% (85%-90%) | 97% (93%-99%) | 100% (99%-100%) |
| 19A | - | 92% (90%-94%) | 98% (96%-99%) | 100% (99%-100%) |
| 22F | - | 94% (92%-96%) | 98% (97%-99%) | 99% (99%-100%) |
| 33F | - | 47% (43%-51%) | 90% (86%-93%) | 99% (98%-100%) |
| 8 | - | 96% (95%-98%) | 99% (95%-100%) | 99% (99%-100%) |
| 10A | - | 29% (25%-33%) | 78% (59%-90%) | 98% (96%-99%) |
| 11A | - | 94% (92%-96%) | 97% (83%-99%) | 99% (98%-99%) |
| 12F | - | 30% (27%-34%) | 75% (65%-83%) | 97% (94%-98%) |
| 15B | - | 94% (92%-96%) | 98% (97%-99%) | 100% (99%-100%) |
| PCV10-SII‡ | | | | |
| 6B | - | 85% (79%-89%) | 84% (71%-92%) | 100% (98%-100%) |
| 9V | - | 95% (91%-97%) | 95% (94%-96%) | 99% (96%-100%) |
| 14 | - | 99% (96%-100%) | 99% (98%-99%) | 98% (96%-100%) |
| 19F | - | 100% (98%-100%) | 98% (97%-99%) | 100% (98%-100%) |
| 23F | - | 96% (92%-98%) | 93% (91%-96%) | 98% (95%-99%) |
| 1 | - | 100% (97%-100%) | 100% (99%-100%) | 100% (98%-100%) |

| Serotype | Seroresponse rate (95%CI) by vaccine schedule (%) | | | |
|---|---|---|---|---|
| | **Post 1-primary dose** | **Post 2-primary dose** | **Post 3-primary dose** | **Post booster** |
| 5 | - | 97% (93%-99%) | 98% (98%-99%) | 98% (94%-99%) |
| 6A | - | 83% (78%-88%) | 82% (80%-83%) | 98% (95%-99%) |
| 7F | - | 100% (97%-100%) | 98% (96%-99%) | 100% (98%-100%) |
| 19A | - | 98% (95%-99%) | 94% (91%-97%) | 100% (98%-100%) |

*: Only 1 study arm available for PCV20 (Post 2-primary), no pooled estimate or heterogeneity.

‡: Only 1 study arm available for PCV10-SII (Post 2-primary), no pooled estimate or heterogeneity.

Heterogeneity for PCV7 (post 1-primary): $I^2$=96.8%, $\tau^2$=1.531, p <0.01; for PCV7 (post 2-primary): $I^2$=94.6%, $\tau^2$=1.665, p <0.01; for PCV7 (post 3-primary): $I^2$=82.7%, $\tau^2$=0.924, p <0.01; for PCV7 (post booster): $I^2$=98.6%, $\tau^2$=0.348, p <0.01; for PCV13 (post 1-primary): $I^2$=97.3%, $\tau^2$=2.097, p <0.01; for PCV13 (post 2-primary): $I^2$=95.7%, $\tau^2$=1.727, p <0.01; for PCV13 (post 3-primary): $I^2$=91.0%, $\tau^2$=1.199, p <0.01; for PCV13 (post booster): $I^2$=99.3%, $\tau^2$=0.582, p <0.01; for PCV15 (post 2-primary): $I^2$=98.6%, $\tau^2$=1.650, p <0.01; for PCV15 (post 3-primary): $I^2$=88.8%, $\tau^2$=0.610, p <0.01; for PCV15 (post booster): $I^2$=99.4%, $\tau^2$=0.361, p <0.01; for PCV20 (post 3-primary): $I^2$=99.4%, $\tau^2$=0.462, p <0.01; for PCV20 (post booster): $I^2$=97.0%, $\tau^2$=1.334, p <0.01; for PCV10-SII (post 3-primary): $I^2$=99.7%, $\tau^2$=0.298, p <0.01; for PCV10-SII (post booster): $I^2$=97.7%, $\tau^2$=1.462, p <0.01.

## Supplementary Figures

### Supplementary Figure 1. Temporal distribution of study arms based on study start year

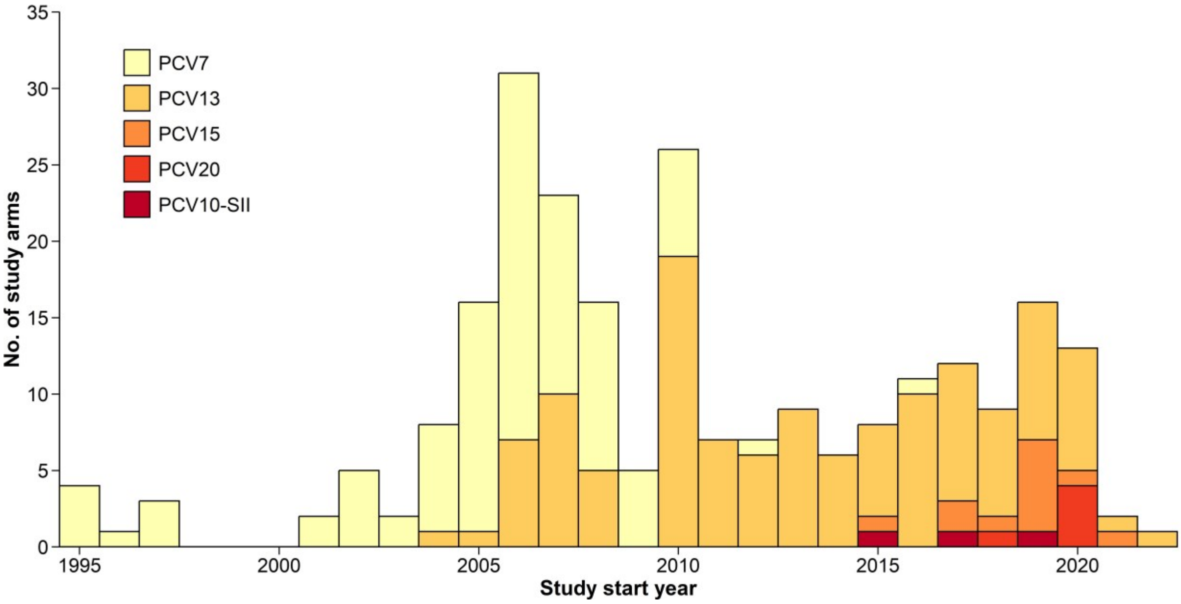


### Supplementary Figure 2. World map of study sites by number of study arms

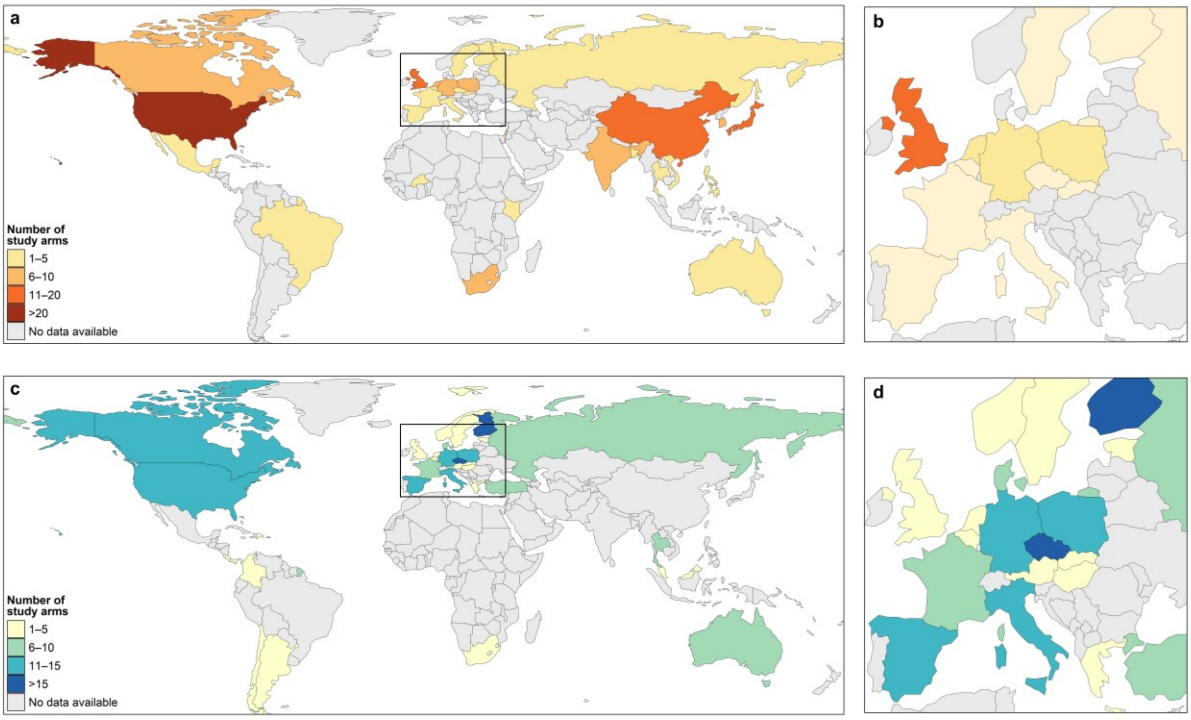


(a) Global distribution of single-country study arms by country. (b) Zoomed-in view of (a) showing single-country study arms in Europe. (c) Global distribution of multi-country study arms by country. (d) Zoomed-in view of (c) showing multi-country study arms in Europe.

***Note***: "multi-country" indicates study arms conducted across multiple sites involving more than one country. In multi-country studies, each study arm was counted for every country involved. In contrast, study arms conducted in a single country were counted only once.

**Supplementary Figure 3. Pneumococcal post-childhood-schedule IgG GMCs (μg/mL) by serotype and vaccine product for all vaccine-covered serotypes**

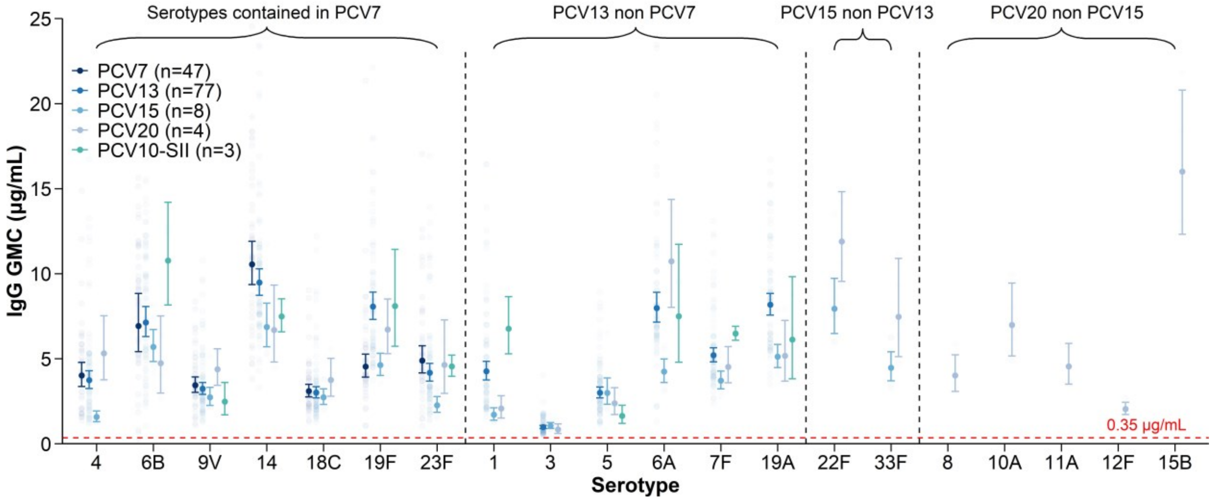


IgG GMCs post-childhood-schedule with different vaccine products.
***Note***: Numbers in parentheses following each vaccine name in the legend indicate the number of study arms included in the meta-analysis contributing to the pooled estimates for that vaccine. The horizontal line represents the 0.35 μg/mL WHO-defined putative protective threshold against IPD, which was developed for post-infant primary series responses and is not a defined correlate for booster doses or other pneumococcal endpoints; it is shown here as a reference line only, noting that serotype-specific protective thresholds may vary.

**Supplementary Figure 4. Pneumococcal post-childhood-schedule seroresponse rates (%) by serotype and vaccine product**

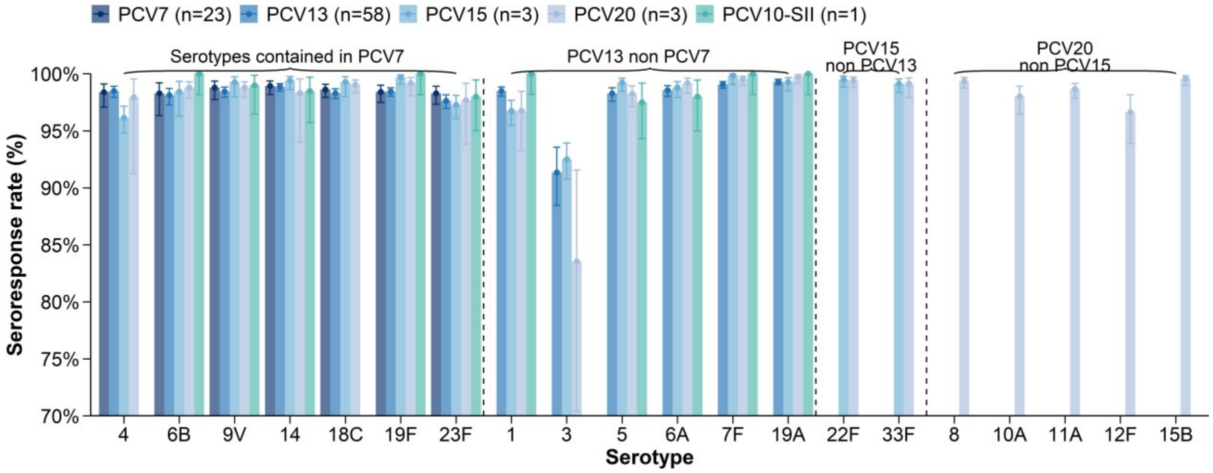


Seroresponse rates post-childhood-schedule with different vaccine products. The seroresponse rate indicates the proportion of participants achieving a predefined antibody response threshold. The y-axes in all panels are restricted to the range of 70–100% to highlight differences across vaccines.
***Note***: Numbers in parentheses following each vaccine name in the legend indicate the number of study arms included in the meta-analysis contributing to the pooled estimates for that vaccine.

**Supplementary Figure 5. Pneumococcal post-childhood-schedule IgG GMCs (μg/mL) by serotype and vaccine product for different assays**

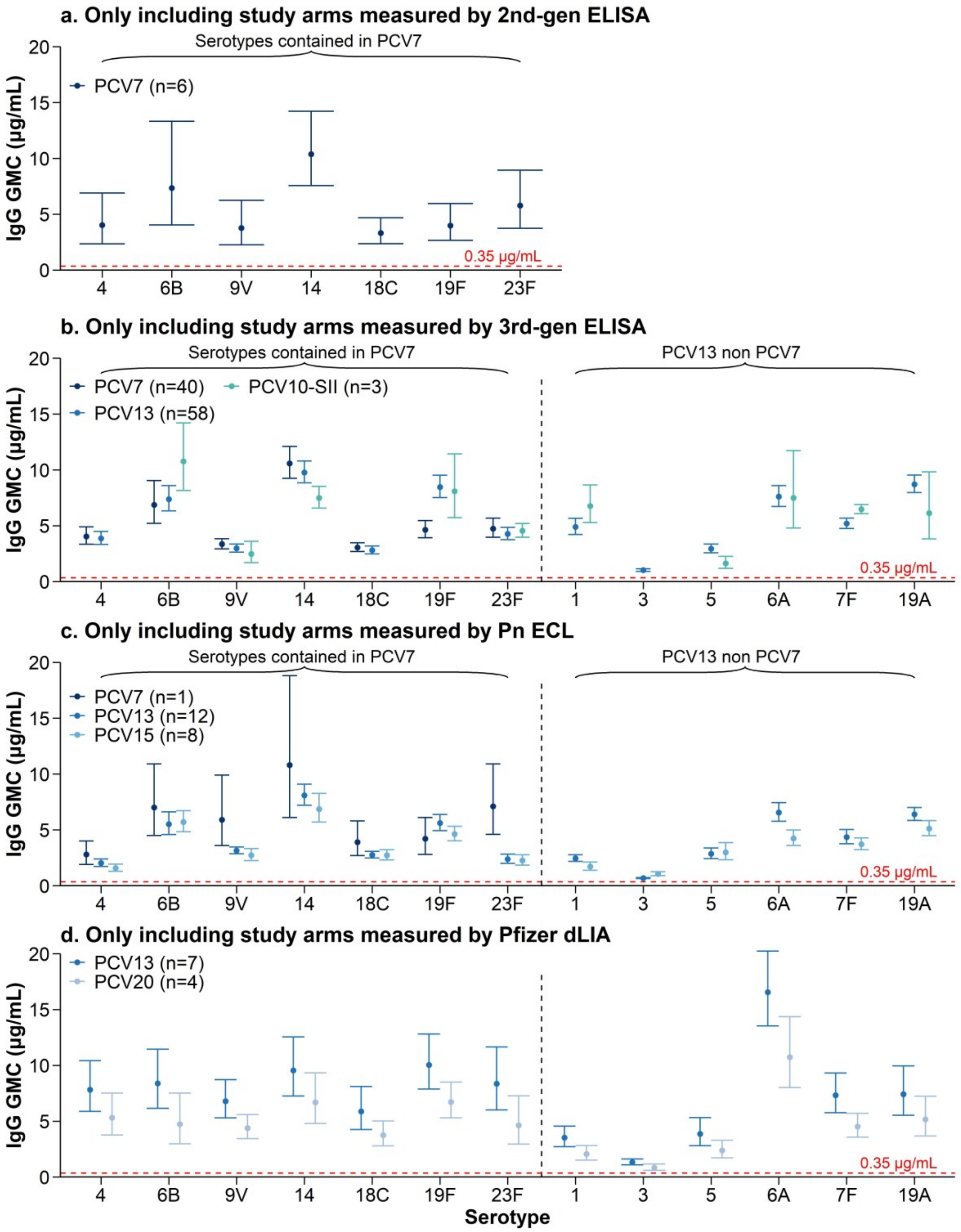


IgG GMCs post-childhood-schedule with different vaccine products measured by different laboratory methods. Panel (a) Study arms used second-generation ELISA to measure IgG response, (b) Study arms used third-generation ELISA (WHO reference ELISA) to measure IgG response, (c) Study arms used Pn ECL to measure IgG response, and (d) Study arms used Pfizer dLIA to measure IgG response.

***Note***: Numbers in parentheses following each vaccine name in the legend indicate the number of study arms included in the meta-analysis contributing to the pooled estimates for that vaccine. The horizontal line in each panel represents the 0.35 μg/mL WHO-defined putative protective threshold against IPD, which was developed for post-infant primary series responses and is not a defined correlate for booster doses or other pneumococcal endpoints; it is shown here as a reference line only, noting that serotype-specific protective thresholds may vary.

**Supplementary Figure 6. Pneumococcal post-childhood-schedule IgG GMCs (µg/mL) for PCV10-SII vs PCV13 by serotype in African Region**

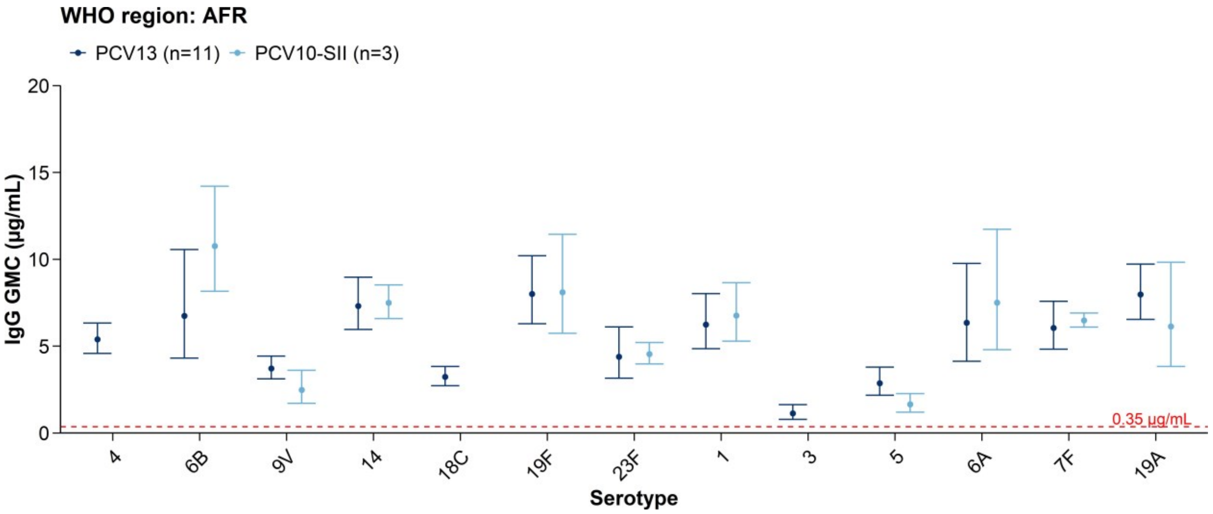


IgG GMCs post-childhood-schedule with different vaccine products in African Region.
***Note***: Numbers in parentheses following each vaccine name in the legend indicate the number of study arms included in the meta-analysis contributing to the pooled estimates for that vaccine. The horizontal line in each panel represents the 0.35 µg/mL WHO-defined putative protective threshold against IPD, which was developed for post-infant primary series responses and is not a defined correlate for booster doses or other pneumococcal endpoints; it is shown here as a reference line only, noting that serotype-specific protective thresholds may vary.

**Supplementary Figure 7. Pneumococcal post-childhood-schedule IgG GMC (µg/mL) by serotype, vaccine product and vaccine schedule**

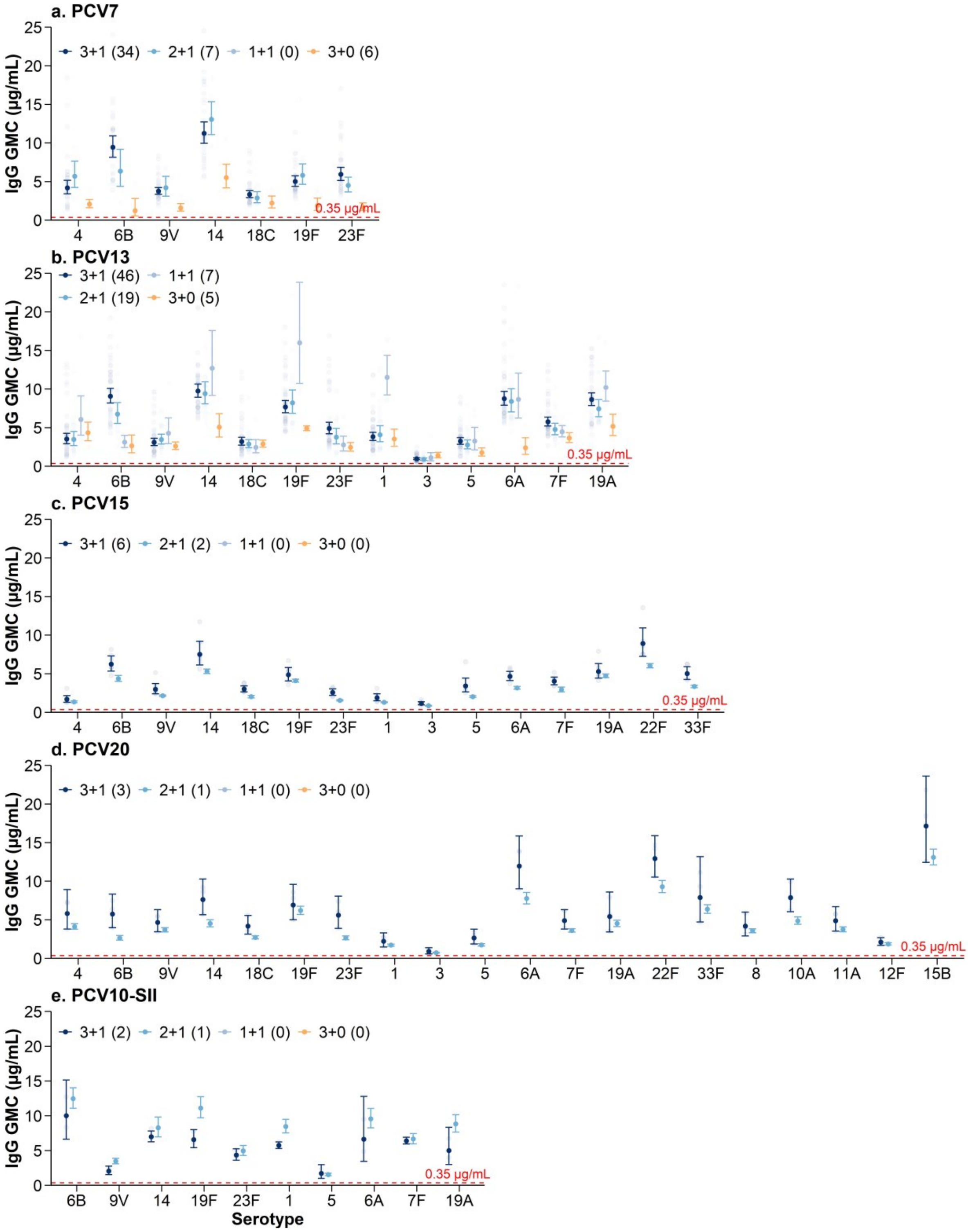


IgG GMCs post-childhood-schedule with different vaccine products and schedules. Panel (a) shows results for PCV7, (b) for PCV13, (c) for PCV15, (d) for PCV20, and (e) for PCV10-SII.

***Note***: Numbers in parentheses following each vaccine name in the legend indicate the number of study arms included in the meta-analysis contributing to the pooled estimates for that vaccine. The horizontal line in each panel represents the 0.35 µg/mL WHO-defined putative protective threshold against IPD, which was developed for post-infant primary series responses and is not a defined correlate for booster doses or other pneumococcal endpoints; it is shown here as a reference line only, noting that serotype-specific protective thresholds may vary.

**Supplementary Figure 8. Pneumococcal post-childhood-schedule seroresponse rates (%) by serotype, vaccine product and vaccine schedule**

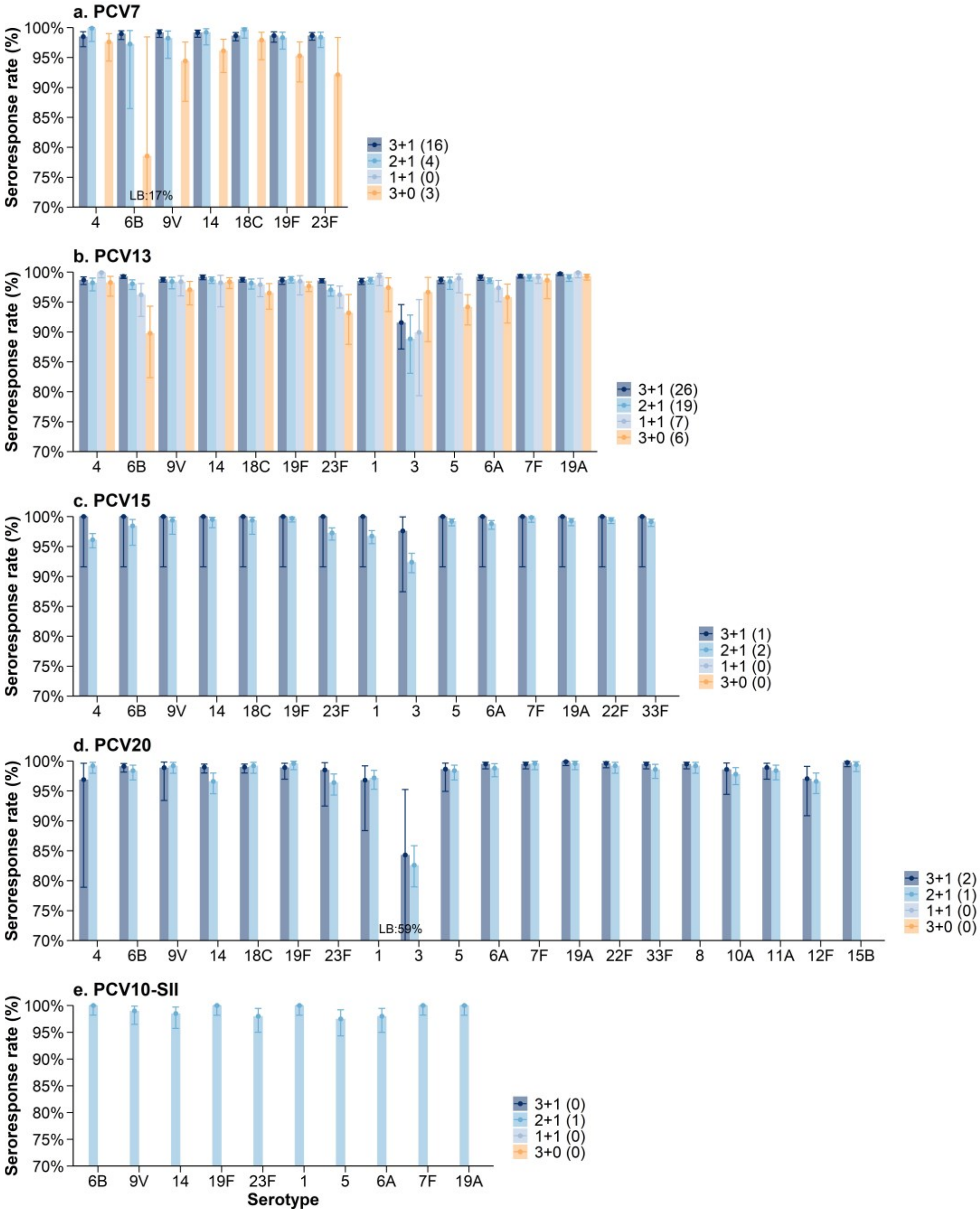


Seroresponse rates post-childhood-schedule with different vaccine products and schedules. Panel (a) shows results for PCV7, (b) for PCV13, (c) for PCV15, (d) for PCV20, and (e) for PCV10-SII. The seroresponse rate indicates the proportion of participants achieving a predefined antibody response threshold. The y-axes in all panels are restricted to the range of 80–100% to highlight differences across vaccines. LB denotes lower bound. ***Note***: Numbers in parentheses following each vaccine name in the legend indicate the number of study arms included in the meta-analysis contributing to the pooled estimates for that vaccine.

**Supplementary Figure 9. Pneumococcal IgG GMC (µg/mL) post "3+1" vaccination schedule by serotype, vaccine product and region**

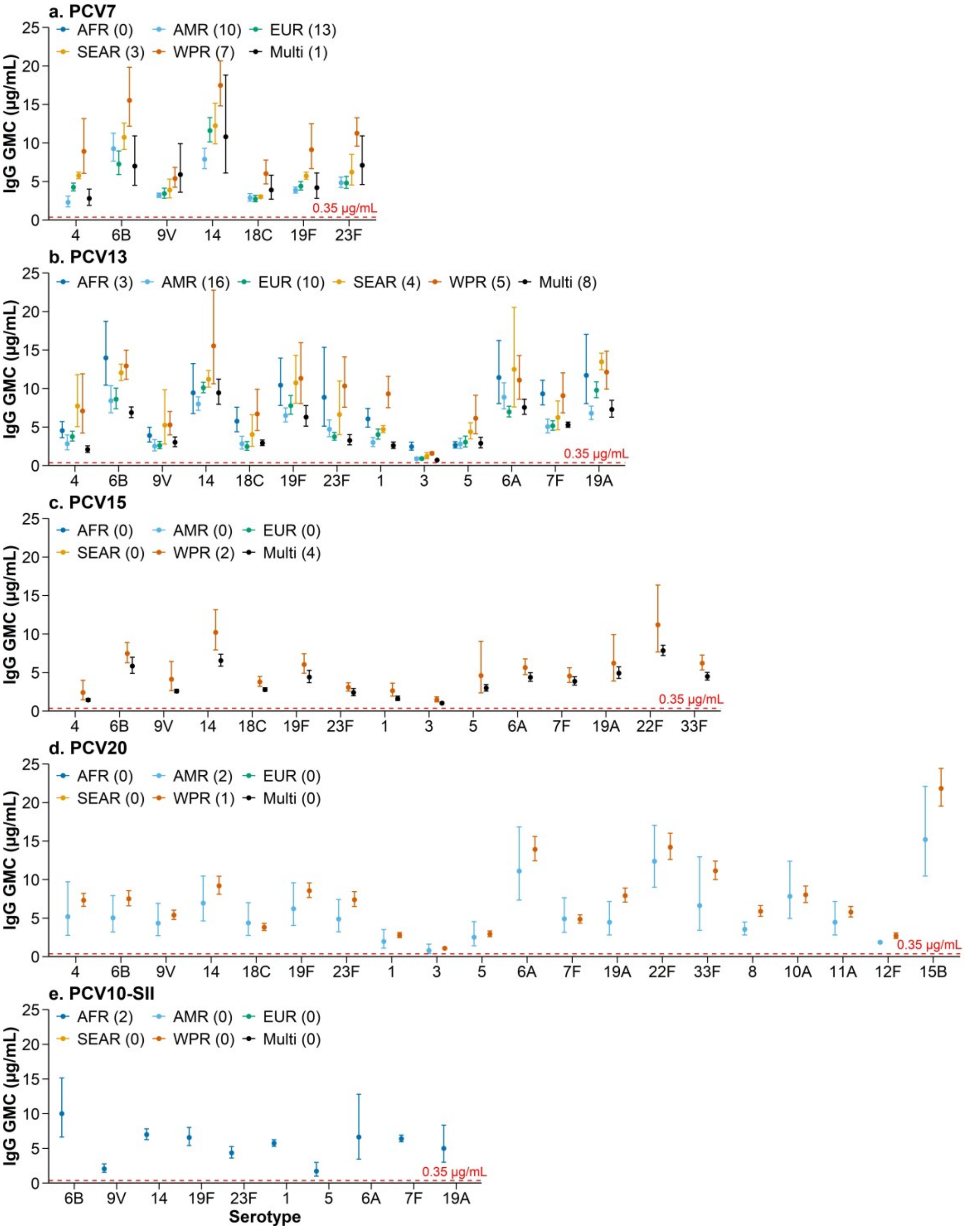


IgG GMCs post-childhood-schedule with different vaccine products using a 3+1 schedule, stratified by WHO regions. Panel (a) shows results for PCV7, (b) for PCV13, (c) for PCV15, (d) for PCV20, and (e) for PCV10-SII.

***Note***: "Multi region" indicates study arms conducted across multiple sites involving more than one WHO region. Numbers in parentheses following each vaccine name in the legend indicate the number of study arms included in the meta-analysis contributing to the pooled estimates for that vaccine. The horizontal line in each panel represents the 0.35 µg/mL WHO-defined putative protective threshold against IPD, which was developed for post-infant primary series responses and is not a defined correlate for booster doses or other disease endpoints; it is shown here as a reference line only, noting that serotype-specific protective thresholds may vary.

***Abbreviations:*** AFR: African Region; AMR: Region of the Americas; EUR: European Region; SEAR: South-East Asia Region; WPR: Western Pacific Region; Multi: Multi-regions.

**Supplementary Figure 10. Pneumococcal seroresponse rates (%) post "3+1" vaccination schedule by serotype, vaccine product and region**

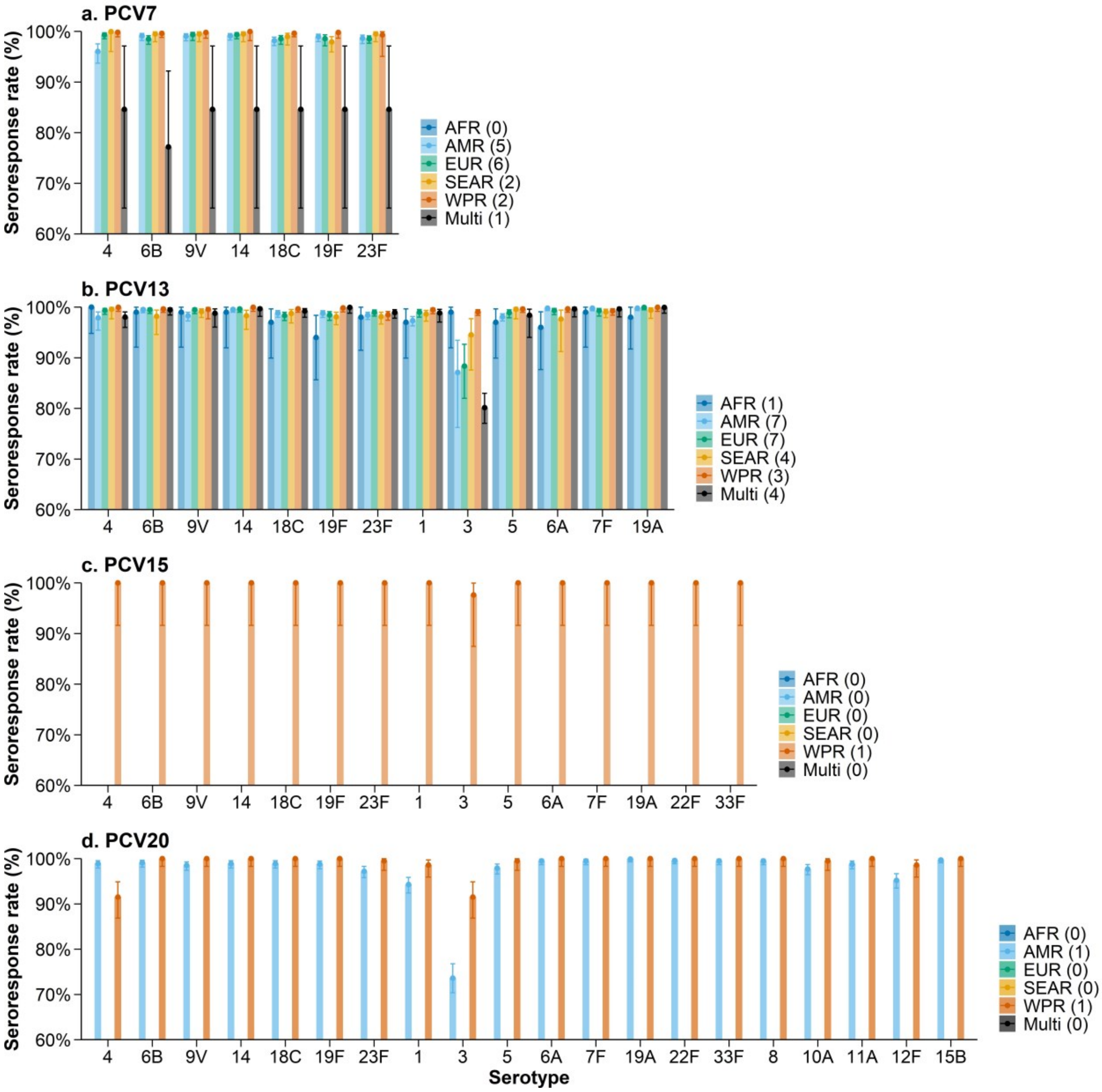


Seroresponse rates post-childhood-schedule with different vaccine products using a 3+1 schedule, stratified by WHO regions. Panel (a) shows results for PCV7, (b) for PCV13, (c) for PCV15, and (d) for PCV20. The seroresponse rate indicates the proportion of participants achieving a predefined antibody response threshold. The y-axes in all panels are restricted to the range of 80–100% to highlight differences across vaccines.
***Note***: "Multi region" indicates study arms conducted across multiple sites involving more than one WHO region. Numbers in parentheses following each vaccine name in the legend indicate the number of study arms included in the meta-analysis contributing to the pooled estimates for that vaccine. No seroresponse rate data were reported for PCV10-SII study.
***Abbreviations:*** AFR: African Region; AMR: Region of the Americas; EUR: European Region; SEAR: South-East Asia Region; WPR: Western Pacific Region; Multi: Multi regions.

**Supplementary Figure 11. Serotype-specific pneumococcal IgG GMC (µg/mL) post PCV13 vaccination by vaccine schedule and region**

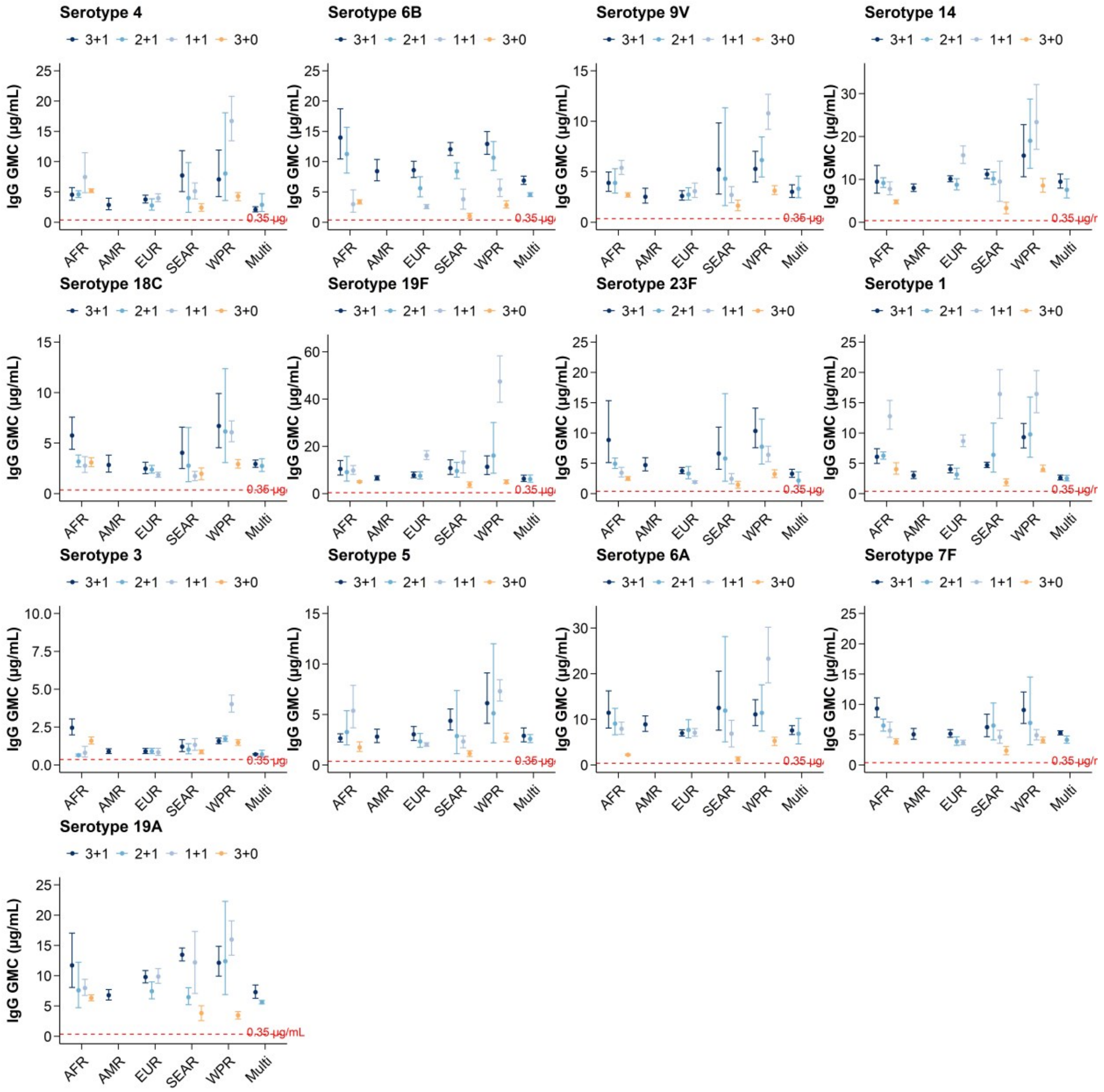


Serotype-specific IgG GMCs post-childhood-schedule with PCV13 vaccination, stratified by WHO regions and schedule.

***Note***: "Multi region" indicates study arms conducted across multiple sites involving more than one WHO region. Numbers in parentheses following each vaccine name in the legend indicate the number of study arms included in the meta-analysis contributing to the pooled estimates for that vaccine. The horizontal line in each panel represents the 0.35 µg/mL WHO-defined putative protective threshold against IPD, which was developed for post-infant primary series responses and is not a defined correlate for booster doses or other pneumococcal endpoints; it is shown here as a reference line only, noting that serotype-specific protective thresholds may vary.

***Abbreviations:*** AFR: African Region; AMR: Region of the Americas; EUR: European Region; SEAR: South-East Asia Region; WPR: Western Pacific Region; Multi: Multi-regions.

**Supplementary Figure 12. Serotype-specific pneumococcal seroresponse rates (%) post PCV13 vaccination by vaccine schedule and region**

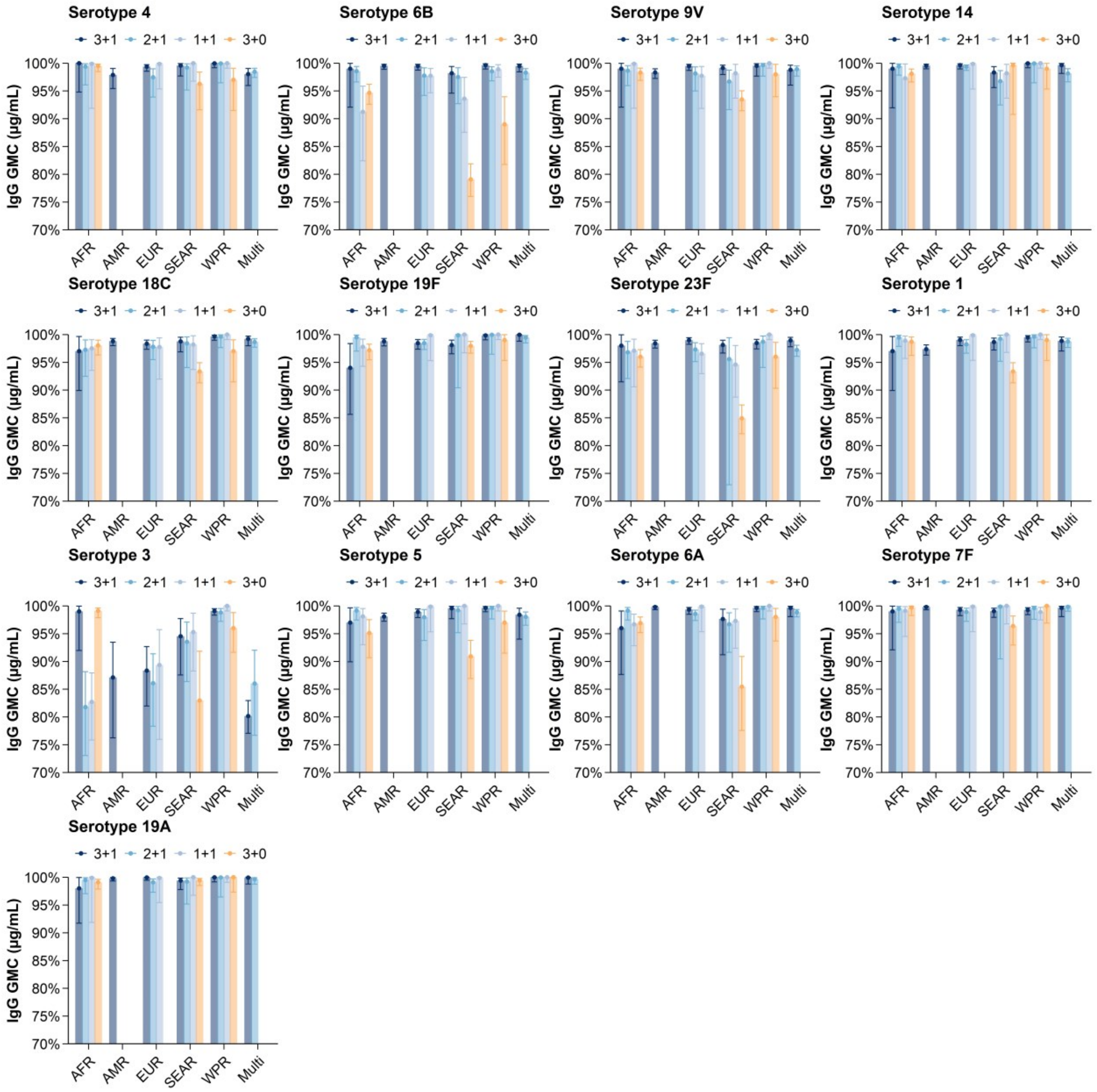


Serotype-specific seroresponse rates post-childhood-schedule with PCV13 vaccination, stratified by WHO regions and schedule.

***Note***: "Multi region" indicates study arms conducted across multiple sites involving more than one WHO region. Numbers in parentheses following each vaccine name in the legend indicate the number of study arms included in the meta-analysis contributing to the pooled estimates for that vaccine.

***Abbreviations:*** AFR: African Region; AMR: Region of the Americas; EUR: European Region; SEAR: South-East Asia Region; WPR: Western Pacific Region; Multi: Multi-regions.

**Supplementary Figure 13. Serotype-specific pneumococcal IgG GMCs (µg/mL) by vaccine product and timepoint**

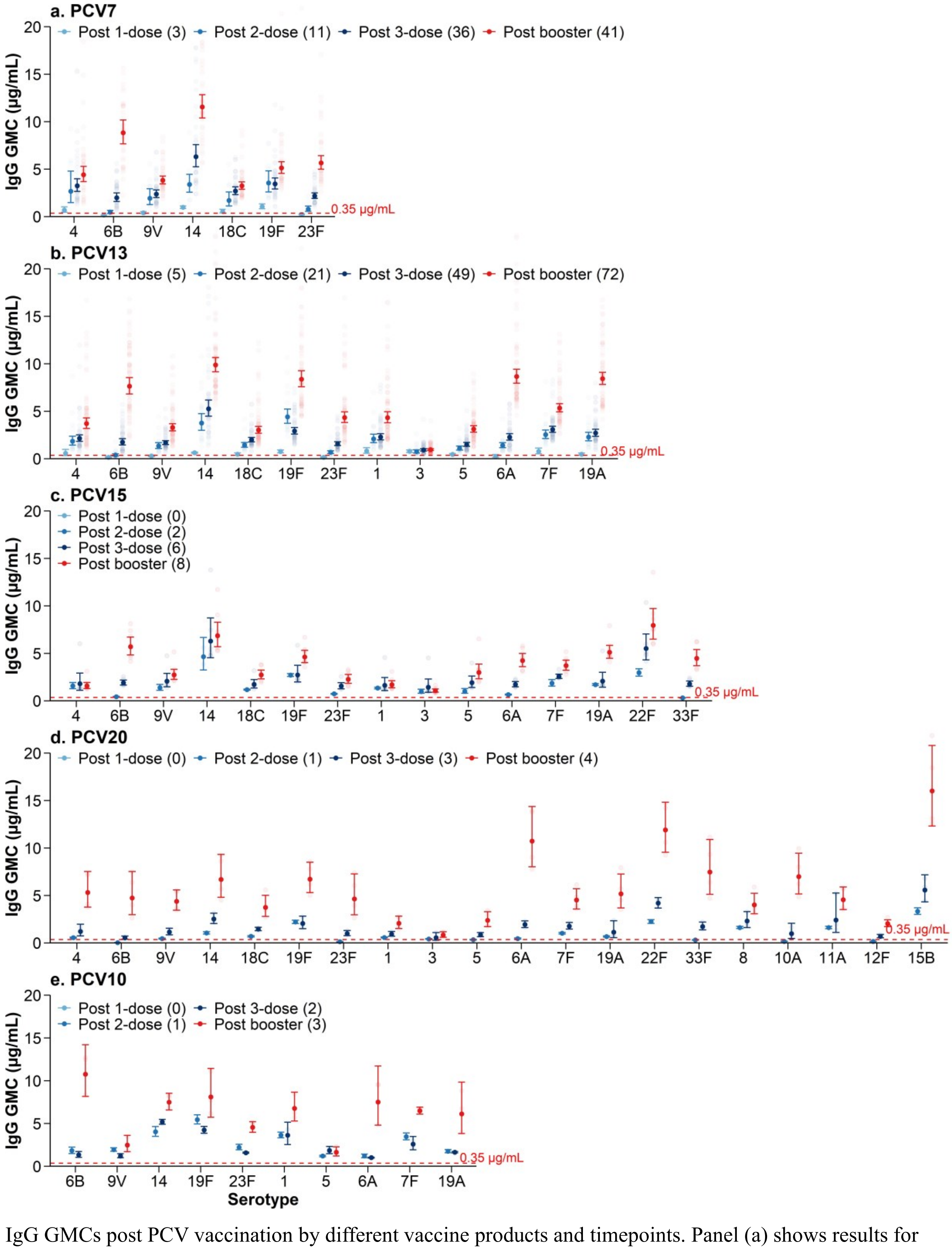


IgG GMCs post PCV vaccination by different vaccine products and timepoints. Panel (a) shows results for PCV7, (b) for PCV13, (c) for PCV15, (d) for PCV20, and (e) for PCV10-SII.

***Note***: Numbers in parentheses following each vaccine name in the legend indicate the number of study arms included in the meta-analysis contributing to the pooled estimates for that vaccine. The horizontal line in each panel represents the 0.35 µg/mL WHO-defined putative protective threshold against IPD, which was developed for post-infant primary series responses and is not a defined correlate for booster doses or other pneumococcal endpoints; it is shown here as a reference line only, noting that serotype-specific protective thresholds may vary.

**Supplementary Figure 14. Post-primary series serotype-specific pneumococcal IgG GMCs (μg/mL) by vaccine product and timepoint**

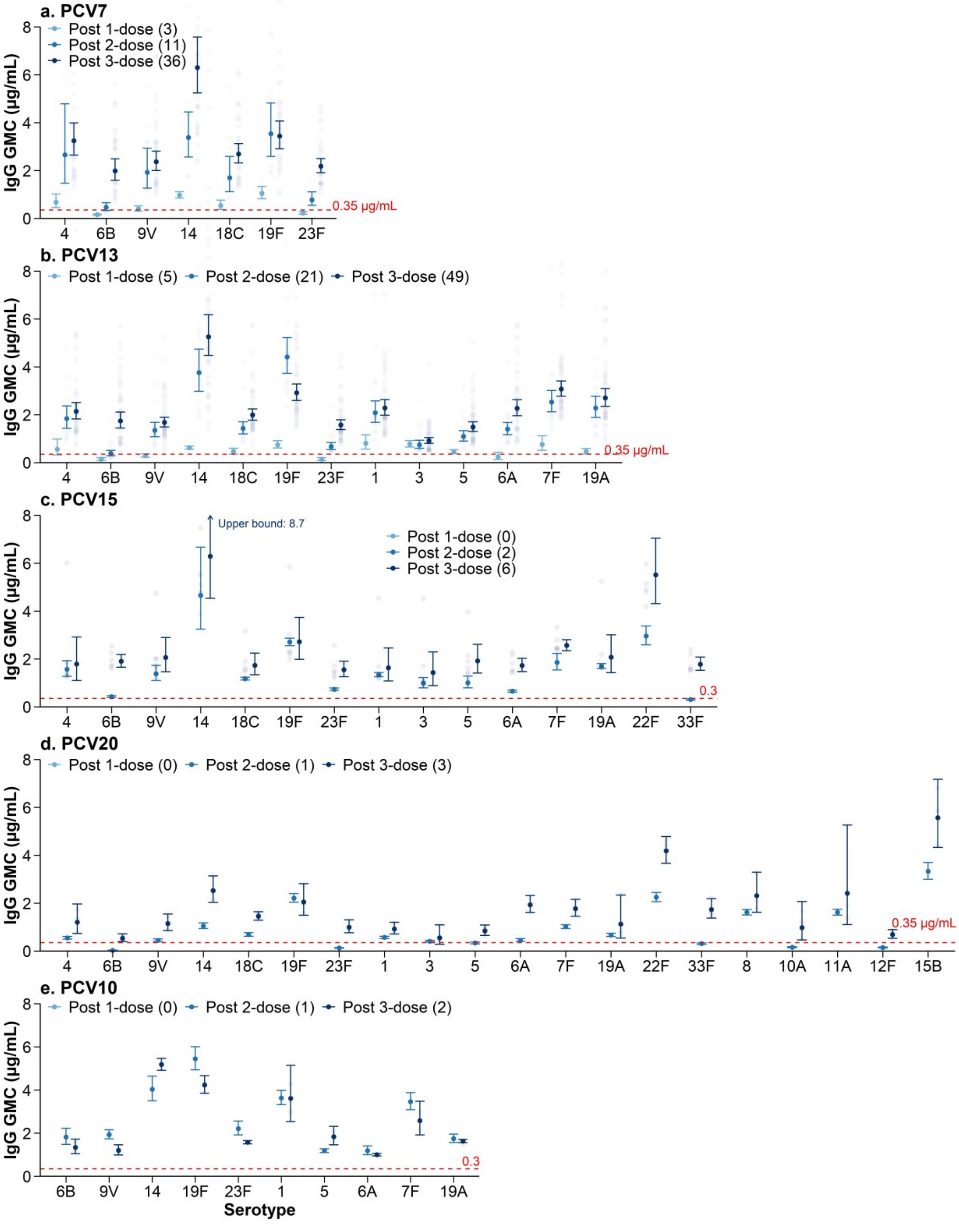


Post-primary series IgG GMCs post PCV vaccination by different vaccine products and timepoints. Panel (a) shows results for PCV7, (b) for PCV13, (c) for PCV15, (d) for PCV20, and (e) for PCV10-SII.

***Note***: Numbers in parentheses following each vaccine name in the legend indicate the number of study arms included in the meta-analysis contributing to the pooled estimates for that vaccine. The horizontal line in each panel represents the 0.35 μg/mL WHO-defined putative protective threshold against IPD, which was developed for post-infant primary series responses and is not a defined correlate for other pneumococcal endpoints; it is shown here as a reference line only, noting that serotype-specific protective thresholds may vary.

**Supplementary Figure 15. Serotype-specific pneumococcal seroresponse rates (%) by vaccine product and timepoint**

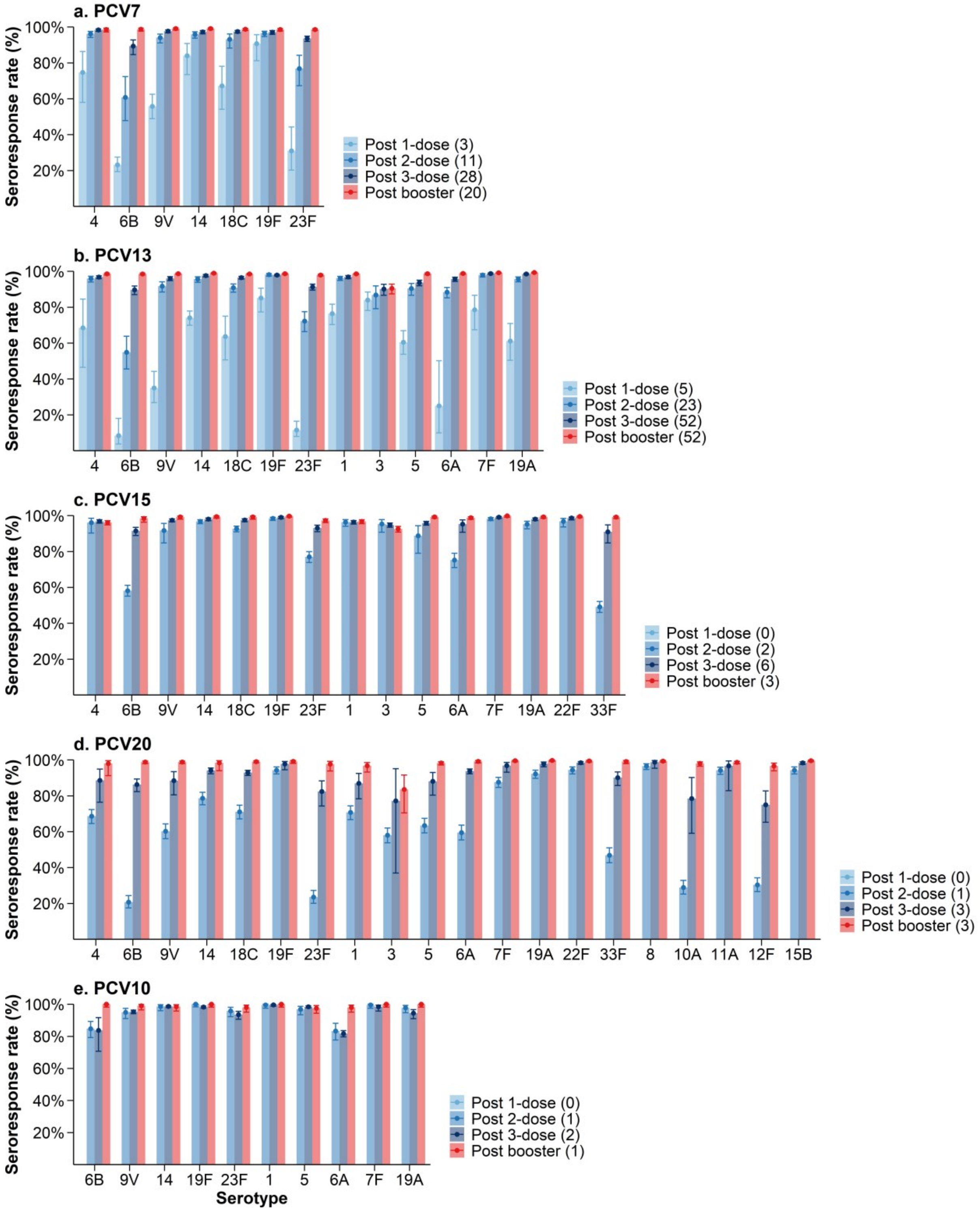


Seroresponse rates post PCV vaccination by different vaccine products and timepoints. Panel (a) shows results for PCV7, (b) for PCV13, (c) for PCV15, (d) for PCV20, and (e) for PCV10-SII. The seroresponse rate indicates the proportion of participants achieving a predefined antibody response threshold.

***Note***: Numbers in parentheses following each vaccine name in the legend indicate the number of study arms included in the meta-analysis contributing to the pooled estimates for that vaccine.

**Supplementary Figure 16. Serotype-specific pneumococcal IgG GMCs (μg/mL) post PCV13 vaccination with "3+1" schedule by timepoint and age of first dose**

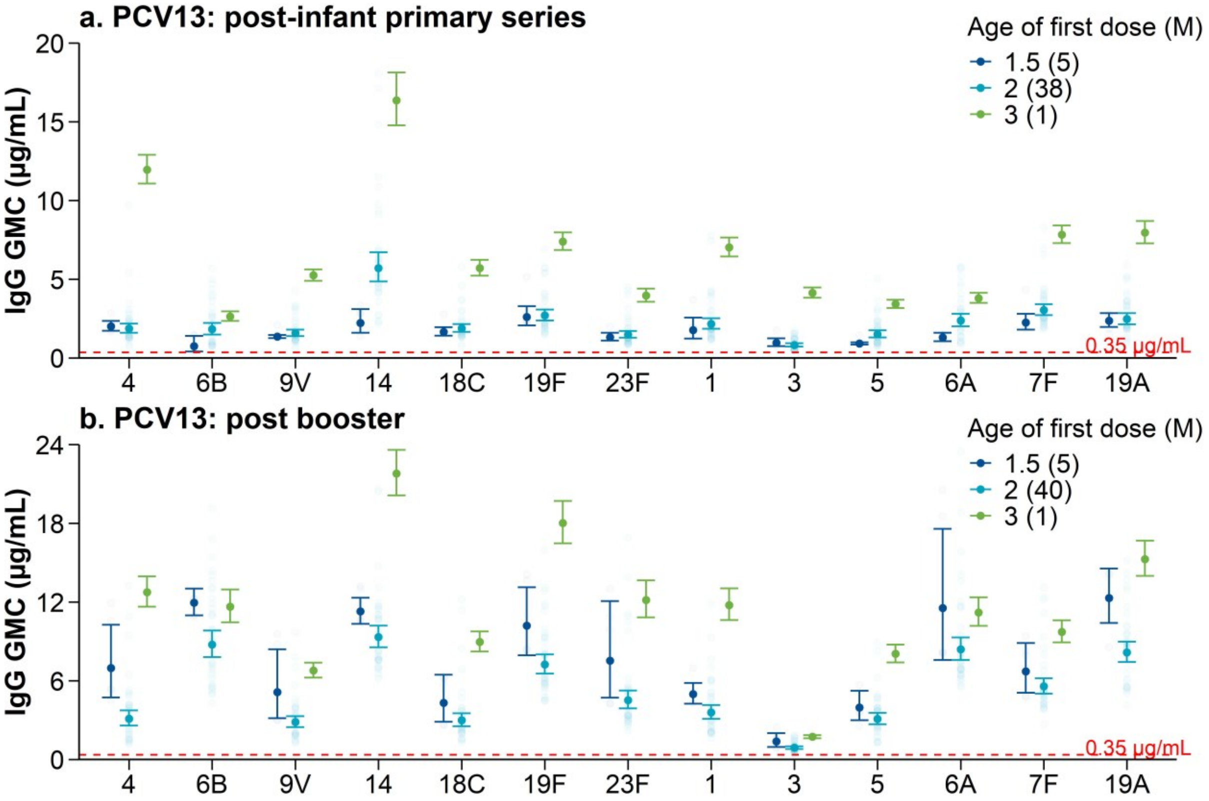


IgG GMCs post PCV13 vaccination by age of first dose. Panel (a) shows results for post primary dose, (b) for post booster.

***Note***: Numbers in parentheses following each vaccine name in the legend indicate the number of study arms included in the meta-analysis contributing to the pooled estimates for that vaccine. The horizontal line in each panel represents the 0.35 μg/mL WHO-defined protective threshold against IPD, which was developed for post-infant primary series responses and is not a defined correlate for booster doses or other pneumococcal endpoints; it is shown here as a reference line only, noting that serotype-specific protective thresholds may vary.

**Supplementary Figure 17. Serotype-specific pneumococcal seroresponse rates (%) post PCV13 vaccination with "3+1" schedule by timepoint and age of first dose**

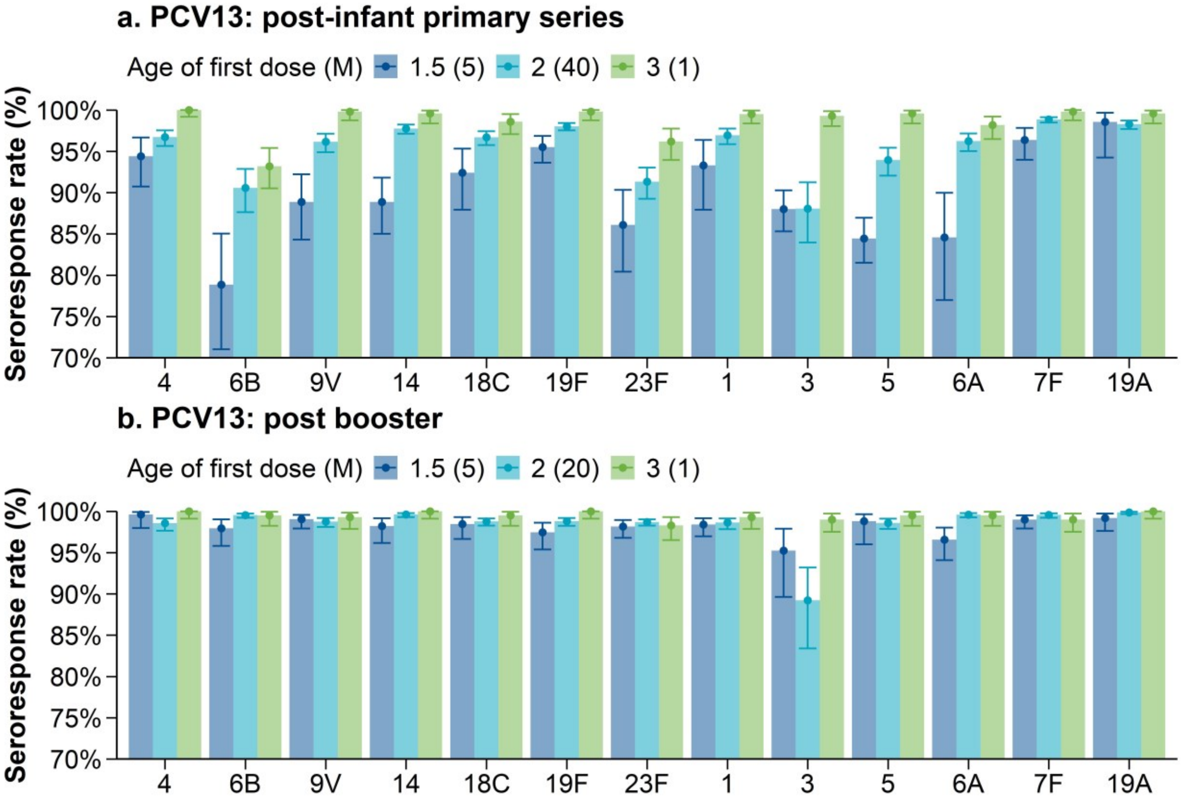


Seroresponse rates post PCV13 vaccination by age of first dose. Panel (a) shows results for post primary dose, (b) for post booster. The seroresponse rate indicates the proportion of participants achieving a predefined antibody response threshold.

***Note***: Numbers in parentheses following each vaccine name in the legend indicate the number of study arms included in the meta-analysis contributing to the pooled estimates for that vaccine.

**Supplementary Figure 18. Serotype-specific pneumococcal IgG GMCs (µg/mL) post PCV13 vaccination with "3+1" schedule by timepoint and interval between primary doses**

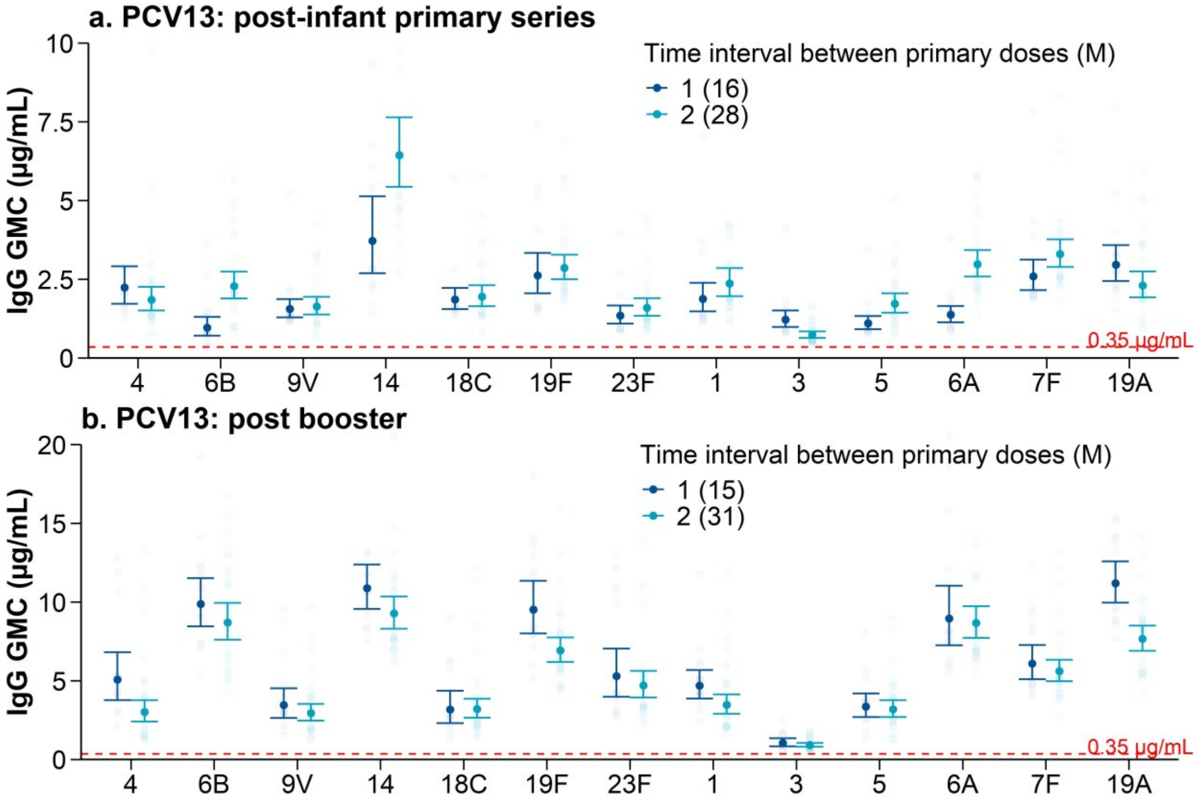


IgG GMCs post PCV13 vaccination by time interval between primary doses. Panel (a) shows results for post primary dose, (b) for post booster.

***Note***: Numbers in parentheses following each vaccine name in the legend indicate the number of study arms included in the meta-analysis contributing to the pooled estimates for that vaccine. The horizontal line in each panel represents the 0.35 µg/mL WHO-defined protective threshold against IPD, which was developed for post-infant primary series responses and is not a defined correlate for booster doses or other pneumococcal endpoints; it is shown here as a reference line only, noting that serotype-specific protective thresholds may vary.

**Supplementary Figure 19. Serotype-specific pneumococcal seroresponse rates (%) post PCV13 vaccination with "3+1" schedule by timepoint and interval between primary doses**

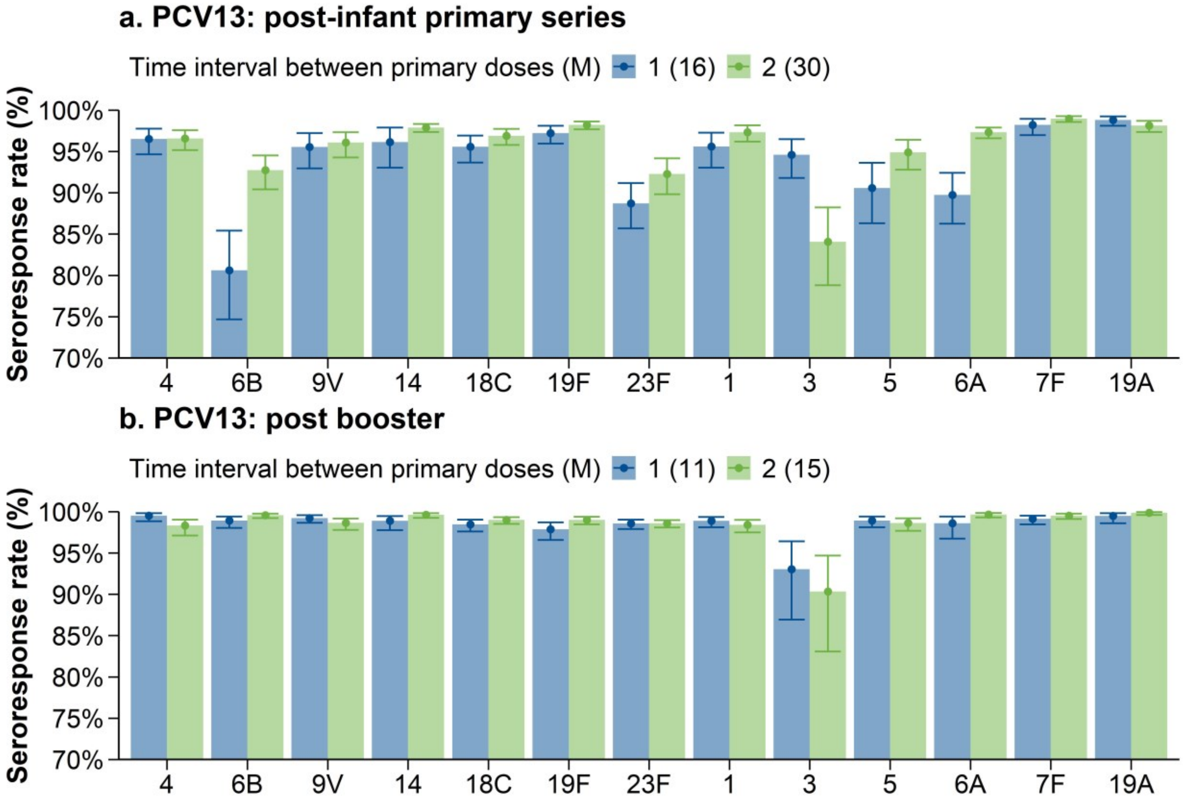


Seroresponse rates post PCV13 vaccination by time interval between primary doses. Panel (a) shows results for post primary dose, (b) for post booster. The seroresponse rate indicates the proportion of participants achieving a predefined antibody response threshold.

***Note***: Numbers in parentheses following each vaccine name in the legend indicate the number of study arms included in the meta-analysis contributing to the pooled estimates for that vaccine.

**Supplementary Figure 20. Sensitivity analysis of pneumococcal post-childhood-schedule IgG GMCs (μg/mL) by serotype and vaccine product (c.f. Fig 2 and Fig S3 which excludes these studies)**

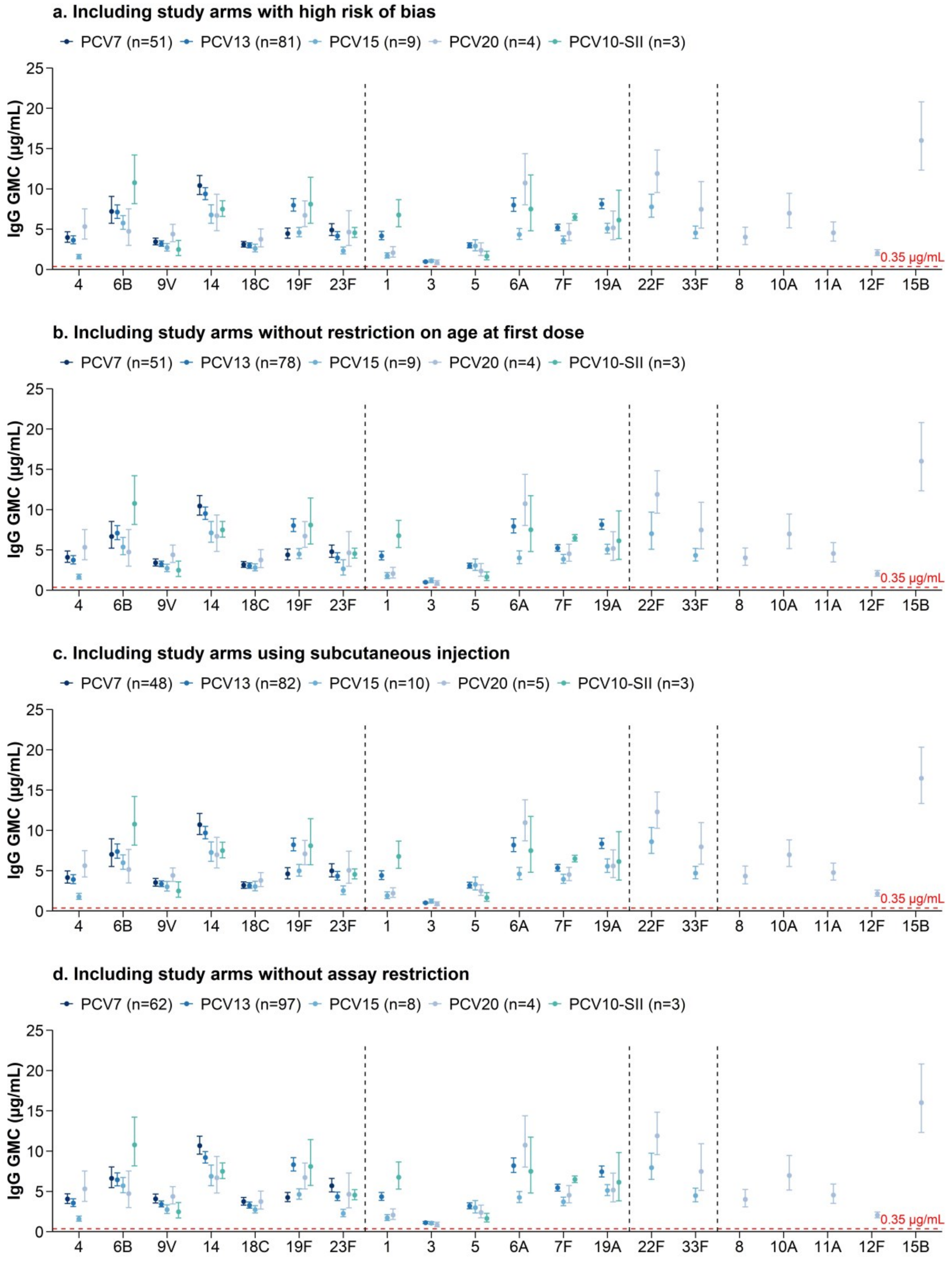


***Note***: Numbers in parentheses following each vaccine name in the legend indicate the number of study arms included in the meta-analysis contributing to the pooled estimates for that vaccine. The horizontal line in each panel represents the 0.35 μg/mL WHO-defined protective threshold against IPD, which was developed for post-infant primary series responses and is not a defined correlate for booster doses or other pneumococcal endpoints; it is shown here as a reference line only, noting that serotype-specific protective thresholds may vary.

**Supplementary Figure 21. Sensitivity analysis of pneumococcal post-childhood-schedule seroresponse rates (%) by serotype and vaccine product (c.f. Fig S5 which excludes these studies)**

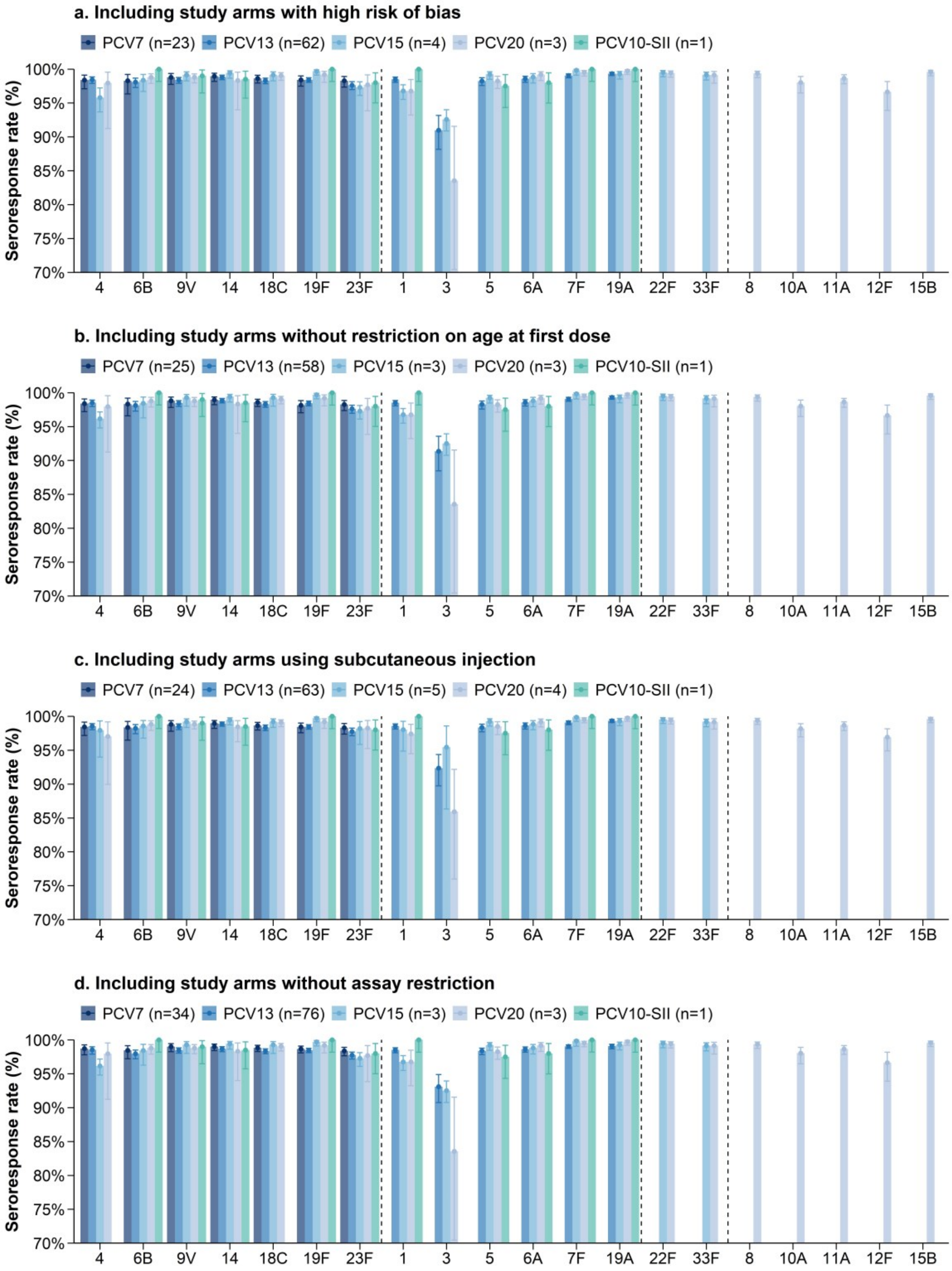


The seroresponse rate indicates the proportion of participants achieving a predefined antibody response threshold. The y-axes in all panels are restricted to the range of 70–100% to highlight differences across vaccines.

## Supplementary Figure 22. The funnel plot analysis for PCV7

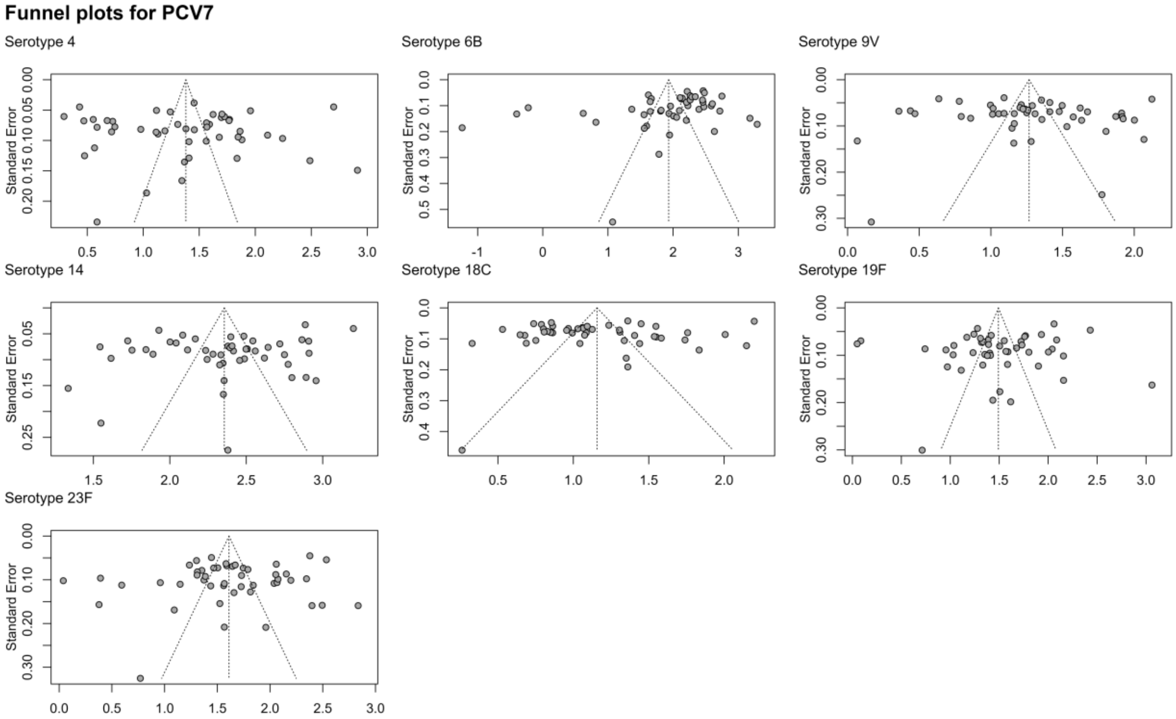

## Supplementary Figure 23. The funnel plot analysis for PCV13

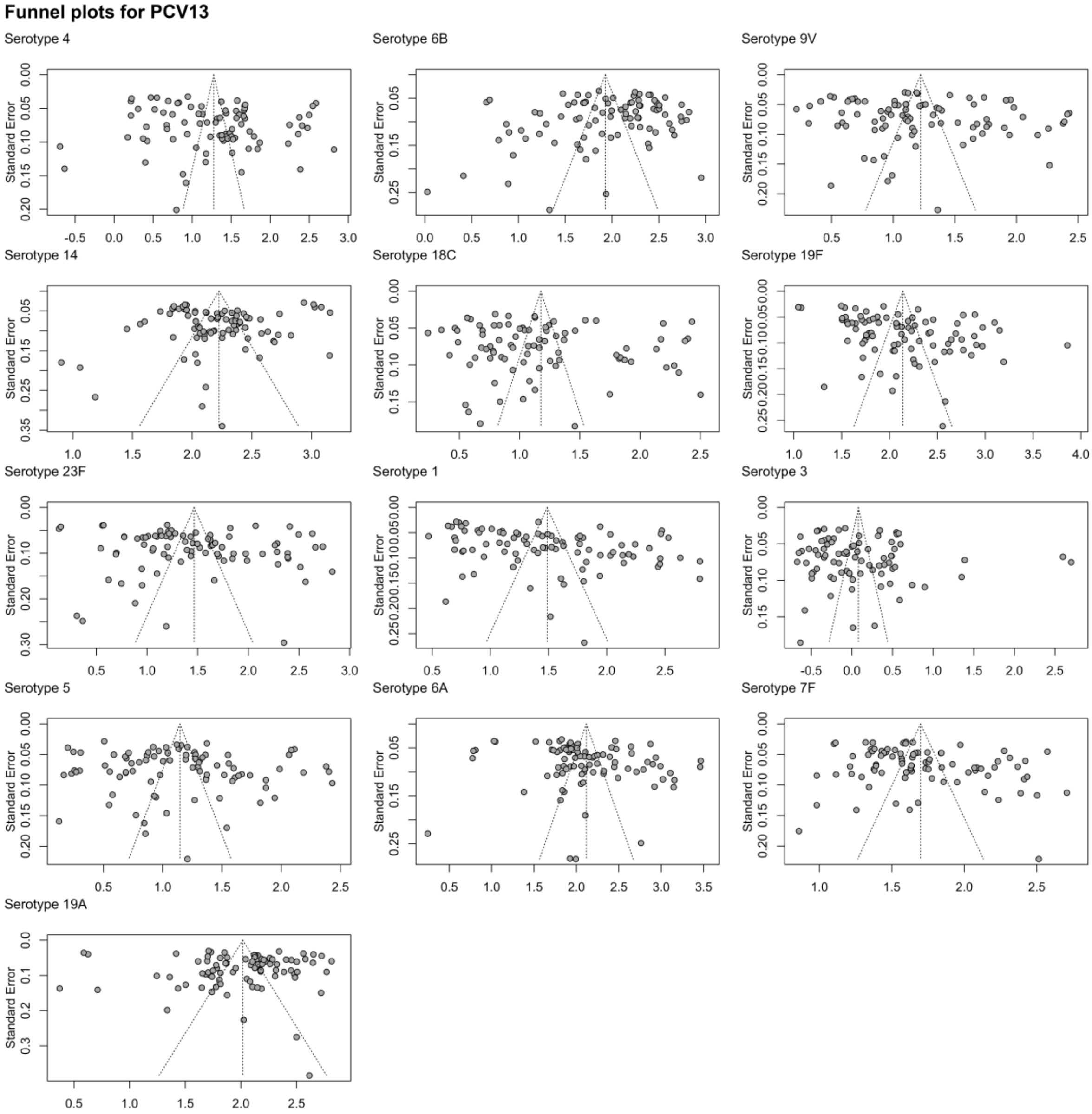

## Supplementary References

1. Koskela M, Leinonen M. Comparison of elisa and ria for measurement of pneumococcal antibodies before and after vaccination with 14-valent pneumococcal capsular polysaccharide vaccine. *J Clin Pathol* **34**,93-8 (1981).
2. Quataert SA, Kirch CS, Wiedl LJ, et al. Assignment of weight-based antibody units to a human antipneumococcal standard reference serum, lot 89-s. *Clin Diagn Lab Immunol* **2**,590-7 (1995).
3. Wernette CM, Frasch CE, Madore D, et al. Enzyme-linked immunosorbent assay for quantitation of human antibodies to pneumococcal polysaccharides. *Clin Diagn Lab Immunol* **10**,514-9 (2003).
4. Concepcion Nelydia F, Frasch Carl E. Pneumococcal type 22f polysaccharide absorption improves the specificity of a pneumococcal-polysaccharide enzyme-linked immunosorbent assay. *Clinical Diagnostic Laboratory Immunology* **8**,266-72 (2001).
5. World Health Organization. Training manual for enzyme linked immunosorbent assay for the quantitation of streptococcus pneumoniae serotype specific igg (pn ps elisa) (89sf version). 2004.
6. World Health Organization. Training manual for enzyme linked immunosorbent assay for the quantitation of streptococcus pneumoniae serotype specific igg (pn ps elisa) (007sp version). 2011.
7. Quataert SA, Rittenhouse-Olson K, Kirch CS, et al. Assignment of weight-based antibody units for 13 serotypes to a human antipneumococcal standard reference serum, lot 89-s(f). *Clin Diagn Lab Immunol* **11**,1064-9 (2004).
8. Poolman JT, Frasch CE, Käyhty H, Lestrate P, Madhi SA, Henckaerts I. Evaluation of pneumococcal polysaccharide immunoassays using a 22f adsorption step with serum samples from infants vaccinated with conjugate vaccines. *Clin Vaccine Immunol* **17**,134-42 (2010).
9. Tan CY, Immermann FW, Sebastian S, et al. Evaluation of a validated luminex-based multiplex immunoassay for measuring immunoglobulin g antibodies in serum to pneumococcal capsular polysaccharides. *mSphere* **3** (2018).
10. Pavliakova D, Giardina PC, Moghazeh S, et al. Development and validation of 13-plex luminex-based assay for measuring human serum antibodies to streptococcus pneumoniae capsular polysaccharides. *mSphere* **3** (2018).
11. Marchese RD, Puchalski D, Miller P, et al. Optimization and validation of a multiplex, electrochemiluminescence-based detection assay for the quantitation of immunoglobulin g serotype-specific antipneumococcal antibodies in human serum. *Clin Vaccine Immunol* **16**,387-96 (2009).
12. Nolan KM, Bonhomme ME, Schier CJ, Green T, Antonello JM, Murphy RD. Optimization and validation of a microcolony multiplexed opsonophagocytic killing assay for 15 pneumococcal serotypes. *Bioanalysis* **12**,1003-20 (2020).
13. Elberse KE, Tcherniaeva I, Berbers GA, Schouls LM. Optimization and application of a multiplex bead-based assay to quantify serotype-specific igg against streptococcus pneumoniae polysaccharides: Response to the booster vaccine after immunization with the pneumococcal 7-valent conjugate vaccine. *Clin Vaccine Immunol* **17**,674-82 (2010).
14. Lal G, Balmer P, Stanford E, Martin S, Warrington R, Borrow R. Development and validation of a nonaplex assay for the simultaneous quantitation of antibodies to nine streptococcus pneumoniae serotypes. *J Immunol Methods* **296**,135-47 (2005).
15. Pickering JW, Martins TB, Greer RW, et al. A multiplexed fluorescent microsphere immunoassay for antibodies to pneumococcal capsular polysaccharides. *Am J Clin Pathol* **117**,589-96 (2002).
16. Rennels MB, Edwards KM, Keyserling HL, et al. Safety and immunogenicity of heptavalent pneumococcal vaccine conjugated to crm197 in united states infants. *Pediatrics* **101**,604-11 (1998).
17. Shinefield HR, Black S, Ray P, et al. Safety and immunogenicity of heptavalent pneumococcal crm197 conjugate vaccine in infants and toddlers. *Pediatr Infect Dis J* **18**,757-63 (1999).
18. Black S, Shinefield H, Fireman B, et al. Efficacy, safety and immunogenicity of heptavalent pneumococcal conjugate vaccine in children. Northern california kaiser permanente vaccine study center group. *Pediatr Infect Dis J* **19**,187-95 (2000).
19. U.S. Food and Drug Administration. Prevnar. 2018.
20. Eskola J, Kilpi T, Palmu A, et al. Efficacy of a pneumococcal conjugate vaccine against acute otitis media. *N Engl J Med* **344**,403-9 (2001).
21. Ekström N, Ahman H, Verho J, et al. Kinetics and avidity of antibodies evoked by heptavalent pneumococcal conjugate vaccines pnccrm and pncompc in the finnish otitis media vaccine trial. *Infect Immun* **73**,369-77 (2005).
22. Ekström N, Väkeväinen M, Verho J, Kilpi T, Käyhty H. Functional antibodies elicited by two heptavalent pneumococcal conjugate vaccines in the finnish otitis media vaccine trial. *Infect Immun* **75**,1794-800 (2007).
23. Tichmann-Schumann I, Soemantri P, Behre U, et al. Immunogenicity and reactogenicity of four doses of diphtheria-tetanus-three-component acellular pertussis-hepatitis b-inactivated polio virus-haemophilus

influenzae type b vaccine coadministered with 7-valent pneumococcal conjugate vaccine. *Pediatr Infect Dis J* **24**,70-7 (2005).
24. Scheifele DW, Halperin SA, Smith B, Ochnio J, Meloff K, Duarte-Monteiro D. Assessment of the compatibility of co-administered 7-valent pneumococcal conjugate, dtap.Ipv/prp-t hib and hepatitis b vaccines in infants 2-7 months of age. *Vaccine* **24**,2057-64 (2006).
25. Scheifele DW, Halperin SA, Ochnio JJ, Mozel M, Duarte-Monteiro D, Wortzman D. Immunologic considerations for the timing of the booster dose of 7-valent pneumococcal conjugate vaccine in young children. *Pediatr Infect Dis J* **26**,387-92 (2007).
26. Knuf M, Habermehl P, Cimino C, Petersen G, Schmitt HJ. Immunogenicity, reactogenicity and safety of a 7-valent pneumococcal conjugate vaccine (pcv7) concurrently administered with a dtpa-hbv-ipv/hib combination vaccine in healthy infants. *Vaccine* **24**,4727-36 (2006).
27. Pichichero ME, Bernstein H, Blatter MM, Schuerman L, Cheuvart B, Holmes SJ. Immunogenicity and safety of a combination diphtheria, tetanus toxoid, acellular pertussis, hepatitis b, and inactivated poliovirus vaccine coadministered with a 7-valent pneumococcal conjugate vaccine and a haemophilus influenzae type b conjugate vaccine. *J Pediatr* **151**,43-9, 49.e1-2 (2007).
28. O'Brien KL, Moisi J, Moulton LH, et al. Predictors of pneumococcal conjugate vaccine immunogenicity among infants and toddlers in an american indian pncrm7 efficacy trial. *J Infect Dis* **196**,104-14 (2007).
29. Millar EV, O'Brien KL, Bronsdon MA, et al. Anticapsular serum antibody concentration and protection against pneumococcal colonization among children vaccinated with 7-valent pneumococcal conjugate vaccine. *Clin Infect Dis* **44**,1173-9 (2007).
30. Li RC, Li FX, Li YP, et al. Safety and immunogenicity of a 7-valent pneumococcal conjugate vaccine (prevenar): Primary dosing series in healthy chinese infants. *Vaccine* **26**,2260-9 (2008).
31. Wyeth. Study evaluating 7-valent pneumococcal conjugate vaccine in healthy infants. https://clinicaltrials.gov/study/NCT00488826 (2010)
32. Li R, Fang KX, Young M, Jr., et al. Long-term antibody persistence study (3 years after last dose) of the 7-valent pneumococcal conjugate vaccine in young children in china. *Vaccine* **34**,5359-65 (2016).
33. Olivier C, Belohradsky BH, Stojanov S, Bonnet E, Petersen G, Liese JG. Immunogenicity, reactogenicity, and safety of a seven-valent pneumococcal conjugate vaccine (pcv7) concurrently administered with a fully liquid dtpa-ipv-hbv-hib combination vaccine in healthy infants. *Vaccine* **26**,3142-52 (2008).
34. Dennehy PH, Bertrand HR, Silas PE, Damaso S, Friedland LR, Abu-Elyazeed R. Coadministration of rix4414 oral human rotavirus vaccine does not impact the immune response to antigens contained in routine infant vaccines in the united states. *Pediatrics* **122**,e1062-6 (2008).
35. Trofa AF, Levin M, Marchant CD, Hedrick J, Blatter MM. Immunogenicity and safety of an inactivated hepatitis a vaccine administered concomitantly with a pneumococcal conjugate vaccine in healthy children 15 months of age. *Pediatr Infect Dis J* **27**,658-60 (2008).
36. GlaxoSmithKline. Immune response & safety of a hepatitis a vaccine given together with a pneumococcal vaccine in healthy children 15 m of age. https://clinicaltrials.gov/study/NCT00197002 (2018)
37. Vesikari T, Wysocki J, Chevallier B, et al. Immunogenicity of the 10-valent pneumococcal non-typeable haemophilus influenzae protein d conjugate vaccine (phid-cv) compared to the licensed 7vcrm vaccine. *Pediatr Infect Dis J* **28**,S66-76 (2009).
38. GlaxoSmithKline. Safety and immunogenicity study of a booster dose of gsk biologicals' 10-valent pneumococcal conjugate vaccine. https://clinicaltrials.gov/study/NCT00370396 (2019)
39. Wysocki J, Tejedor JC, Grunert D, et al. Immunogenicity of the 10-valent pneumococcal non-typeable haemophilus influenzae protein d conjugate vaccine (phid-cv) when coadministered with different neisseria meningitidis serogroup c conjugate vaccines. *Pediatr Infect Dis J* **28**,S77-88 (2009).
40. Bermal N, Szenborn L, Chrobot A, et al. The 10-valent pneumococcal non-typeable haemophilus influenzae protein d conjugate vaccine (phid-cv) coadministered with dtpw-hbv/hib and poliovirus vaccines: Assessment of immunogenicity. *Pediatr Infect Dis J* **28**,S89-96 (2009).
41. Bermal N, Szenborn L, Edison A, et al. Safety and immunogenicity of a booster dose of the 10-valent pneumococcal nontypeable haemophilus influenzae protein d conjugate vaccine coadministered with dtpw-hbv/hib and poliovirus vaccines. *Pediatr Infect Dis J* **30**,69-72 (2011).
42. GlaxoSmithKline. Safety and immunogenicity study of glaxosmithkline (gsk) biologicals' 10-valent pneumococcal conjugate vaccine. https://clinicaltrials.gov/study/NCT00344318 (2018)
43. GlaxoSmithKline. Pneumococcal vaccine booster study in healthy children 12-18 mths old previously primed with the same vaccines. https://clinicaltrials.gov/study/NCT00547248 (2019)
44. Givon-Lavi N, Greenberg D, Dagan R. Immunogenicity of alternative regimens of the conjugated 7-valent pneumococcal vaccine: A randomized controlled trial. *Pediatr Infect Dis J* **29**,756-62 (2010).

45. Dagan R, Givon-Lavi N, Greenberg D, Fritzell B, Siegrist CA. Nasopharyngeal carriage of streptococcus pneumoniae shortly before vaccination with a pneumococcal conjugate vaccine causes serotype-specific hyporesponsiveness in early infancy. *J Infect Dis* **201**,1570-9 (2010).
46. Dagan R, Givon-Lavi N, Porat N, Greenberg D. The effect of an alternative reduced-dose infant schedule and a second year catch-up schedule with 7-valent pneumococcal conjugate vaccine on pneumococcal carriage: A randomized controlled trial. *Vaccine* **30**,5132-40 (2012).
47. Dagan R, Ben-Shimol S, Simell B, et al. A toddler pcv booster dose following 3 infancy priming doses increases circulating serotype-specific igg levels but does not increase protection against carriage. *Vaccine* **36**,2774-82 (2018).
48. Wysocki J, Tansey S, Brachet E, et al. Randomised, controlled trial of concomitant pneumococcal and meningococcal conjugate vaccines. *Vaccine* **28**,7779-86 (2010).
49. Goldblatt D, Southern J, Ashton L, et al. Immunogenicity of a reduced schedule of pneumococcal conjugate vaccine in healthy infants and correlates of protection for serotype 6b in the united kingdom. *Pediatr Infect Dis J* **29**,401-5 (2010).
50. Grimprel E, Wysocki J, Boisnard F, Thomas S, Mwawasi G, Reynolds D. Immunogenicity and safety of fully liquid dtap$_5$-ipv-hib compared with dtap$_3$-ipv/hib when both coadministered with a heptavalent pneumococcal conjugate vaccine (pcv7) at 2, 3, 4, and 12 to 18 months of age: A phase iii, single-blind, randomised, controlled, multicentre study. *Vaccine* **29**,7370-8 (2011).
51. Scott JA, Ojal J, Ashton L, Muhoro A, Burbidge P, Goldblatt D. Pneumococcal conjugate vaccine given shortly after birth stimulates effective antibody concentrations and primes immunological memory for sustained infant protection. *Clin Infect Dis* **53**,663-70 (2011).
52. van den Bergh MR, Spijkerman J, François N, et al. Immunogenicity, safety, and reactogenicity of the 10-valent pneumococcal nontypeable haemophilus influenzae protein d conjugate vaccine and dtpa-ipv-hib when coadministered as a 3-dose primary vaccination schedule in the netherlands: A randomized controlled trial. *Pediatr Infect Dis J* **30**,e170-8 (2011).
53. van den Bergh MR, Spijkerman J, François N, et al. Immunogenicity, safety and reactogenicity of a booster dose of the 10-valent pneumococcal nontypeable h. Influenzae protein d conjugate vaccine coadministered with dtpa-ipv-hib in dutch children: A randomized controlled trial. *Pediatr Infect Dis J* **35**,e206-19 (2016).
54. GlaxoSmithKline. Co-administration of pneumococcal conjugate vaccine with dtpa-ipv-hib versus co-administration with dtpa-hbv-ipv/hib. https://clinicaltrials.gov/study/NCT00652951 (2019)
55. Kim CH, Kim JS, Cha SH, et al. Response to primary and booster vaccination with 10-valent pneumococcal nontypeable haemophilus influenzae protein d conjugate vaccine in korean infants. *Pediatr Infect Dis J* **30**,e235-43 (2011).
56. GlaxoSmithKline. Primary vaccination course in children receiving pneumococcal conjugate vaccine gsk 1024850a or prevenar™ and hiberix™. https://clinicaltrials.gov/study/NCT00680914 (2018)
57. GlaxoSmithKline. Booster vaccination study with a pneumococcal vaccine in children primed with the same vaccine. https://clinicaltrials.gov/study/NCT00911144 (2018)
58. Leonardi M, Bromberg K, Baxter R, et al. Immunogenicity and safety of mmrv and pcv-7 administered concomitantly in healthy children. *Pediatrics* **128**,e1387-94 (2011).
59. Merck Sharp & Dohme LLC. V221 concomitant use study with pneumococcal conjugate vaccine (v221-019). https://clinicaltrials.gov/study/NCT00109343 (2017)
60. Marshall GS, Marchant CD, Blatter M, Friedland LR, Aris E, Miller JM. Co-administration of a novel haemophilus influenzae type b and neisseria meningitidis serogroups c and y-tetanus toxoid conjugate vaccine does not interfere with the immune response to antigens contained in infant vaccines routinely used in the united states. *Hum Vaccin* **7**,258-64 (2011).
61. Blatter MM, Klein NP, Shepard JS, et al. Immunogenicity and safety of two tetravalent (measles, mumps, rubella, varicella) vaccines coadministered with hepatitis a and pneumococcal conjugate vaccines to children twelve to fourteen months of age. *Pediatr Infect Dis J* **31**,e133-40 (2012).
62. GlaxoSmithKline. Immunogenicity and safety of glaxosmithkline biologicals' mmrv vaccine vs. Proquad® in children 12-14 months of age. https://clinicaltrials.gov/study/NCT00578175 (2018)
63. Klein NP, Reisinger KS, Johnston W, et al. Safety and immunogenicity of a novel quadrivalent meningococcal crm-conjugate vaccine given concomitantly with routine vaccinations in infants. *Pediatr Infect Dis J* **31**,64-71 (2012).
64. Novartis Vaccines. A study to evaluate safety and immune response of novartis meningococcal acwy vaccine in infants. https://clinicaltrials.gov/study/NCT00474526 (2014)
65. Tapiéro B, Halperin SA, Dionne M, et al. Safety and immunogenicity of a hexavalent vaccine administered at 2, 4 and 6 months of age with or without a heptavalent pneumococcal conjugate vaccine: A randomized, open-label study. *Pediatr Infect Dis J* **32**,54-61 (2013).

66. Halperin SA, Tapiéro B, Dionne M, et al. Safety and immunogenicity of a toddler dose following an infant series of a hexavalent diphtheria, tetanus, acellular pertussis, inactivated poliovirus, haemophilus influenzae type b, hepatitis b vaccine administered concurrently or at separate visits with a heptavalent pneumococcal conjugate vaccine. *Pediatr Infect Dis J* **33**,73-80 (2014).
67. Vesikari T, Esposito S, Prymula R, et al. Immunogenicity and safety of an investigational multicomponent, recombinant, meningococcal serogroup b vaccine (4cmenb) administered concomitantly with routine infant and child vaccinations: Results of two randomised trials. *Lancet* **381**,825-35 (2013).
68. Novartis Vaccines. Immunogenicity, safety and lot to lot consistency of novartis meningococcal b recombinant vaccine when administered with routine infant vaccinations to healthy infants. https://clinicaltrials.gov/study/NCT00657709 (2017)
69. van Westen E, Rodenburg GD, van Gils EJ, et al. Levels and functionality of antibodies after pneumococcal conjugate vaccine in schedules with different timing of the booster dose. *Vaccine* **31**,5834-42 (2013).
70. Rodenburg GD, van Gils EJ, Veenhoven RH, et al. Comparability of antibody response to a booster dose of 7-valent pneumococcal conjugate vaccine in infants primed with either 2 or 3 doses. *Vaccine* **28**,1391-6 (2010).
71. Yetman RJ, Shepard JS, Duke A, et al. Concomitant administration of hepatitis a vaccine with measles/mumps/rubella/varicella and pneumococcal vaccines in healthy 12- to 23-month-old children. *Hum Vaccin Immunother* **9**,1691-7 (2013).
72. Merck Sharp & Dohme LLC. Concomitant use of hepatitis a vaccine with measles, mumps, rubella and varicella vaccine and pneumococcal 7-valent conjugate vaccine in healthy 12-month-old children (v251-067). https://clinicaltrials.gov/study/NCT00312858 (2017)
73. Prymula R, Esposito S, Zuccotti GV, et al. A phase 2 randomized controlled trial of a multicomponent meningococcal serogroup b vaccine (i). *Hum Vaccin Immunother* **10**,1993-2004 (2014).
74. Esposito S, Prymula R, Zuccotti GV, et al. A phase 2 randomized controlled trial of a multicomponent meningococcal serogroup b vaccine, 4cmenb, in infants (ii). *Hum Vaccin Immunother* **10**,2005-14 (2014).
75. López P, Arguedas Mohs A, Abdelnour Vásquez A, et al. A randomized controlled study of a fully liquid dtap-ipv-hb-prp-t hexavalent vaccine for primary and booster vaccinations of healthy infants and toddlers in latin america. *Pediatr Infect Dis J* **36**,e272-e82 (2017).
76. Sanofi Pasteur. Study of the booster effect of dtap-ipv-hep b-prp~t combined vaccine or infanrix hexa and prevenar in healthy infants. https://clinicaltrials.gov/study/NCT01444781 (2014)
77. Zhao Y, Li G, Xia S, et al. Immunogenicity and safety of a novel 13-valent pneumococcal vaccine in healthy chinese infants and toddlers. *Front Microbiol* **13**,870973 (2022).
78. Pfizer. Open-label, randomized, three-arm, phase iiib clinical study to investigate the safety and immunogenicity of a concomitant administration of group c meningococcal polysaccharide-tetanus toxoid conjugate (menc-tt) vaccine and 7-valent pneumococcal crm197-conjugate vaccine (pcv7) in toddlers previously immunized during infancy with pcv7. https://www.clinicaltrialsregister.eu/ctr-search/search?query=eudract_number:2007-004276-39NCT04382326 (2016)
79. Pfizer. A trial evaluating a 7-valent pneumococcal conjugate vaccine given with diphtheria, tetanus, and acellular pertussis vaccine (dtap) in healthy japanese infants. https://clinicaltrials.gov/study/NCT01250756 (2013)
80. Thisyakorn U, Chokephaibulkit K, Kosalaraksa P, Benjaponpitak S, Pancharoen C, Chuenkitmongkol S. Immunogenicity and safety of 23-valent pneumococcal polysaccharide vaccine as a booster dose in 12- to 18-month-old children primed with 3 doses of 7-valent pneumococcal conjugate vaccine. *Hum Vaccin Immunother* **10**,1859-65 (2014).
81. Grimprel E, Laudat F, Patterson S, et al. Immunogenicity and safety of a 13-valent pneumococcal conjugate vaccine (pcv13) when given as a toddler dose to children immunized with pcv7 as infants. *Vaccine* **29**,9675-83 (2011).
82. Wyeth. Study to evaluate a 13-valent pneumococcal conjugate vaccine in infants (nct00366678). https://clinicaltrials.gov/study/NCT00366678 (2012)
83. Wyeth. Study evaluating antibody response of 13-valent pneumococcal conjugate vaccine (13vpnc) 24 months after toddler dose. https://clinicaltrials.gov/study/NCT01026038 (2012)
84. Sobanjo-ter Meulen A, Vesikari T, Malacaman EA, et al. Safety, tolerability and immunogenicity of 15-valent pneumococcal conjugate vaccine in toddlers previously vaccinated with 7-valent pneumococcal conjugate vaccine. *Pediatr Infect Dis J* **34**,186-94 (2015).
85. Merck Sharp & Dohme LLC. A multicenter, double-blind study of the safety, tolerability, and immunogenicity of pneumococcal conjugate vaccine (v114) compared to prevnar™ in healthy adults and toddlers. https://www.clinicaltrialsregister.eu/ctr-search/search?query=eudract_number:2009-015103-58 (2019)
86. Merck Sharp & Dohme LLC. Safety and tolerability study for the pneumococcal conjugate vaccine v114 versus prevnar™ (v114-001). https://clinicaltrials.gov/study/NCT01215175 (2019)

87. Kieninger DM, Kueper K, Steul K, et al. Safety, tolerability, and immunologic noninferiority of a 13-valent pneumococcal conjugate vaccine compared to a 7-valent pneumococcal conjugate vaccine given with routine pediatric vaccinations in germany. *Vaccine* **28**,4192-203 (2010).
88. Wyeth. Study to evaluate a 13-valent pneumococcal conjugate vaccine in infants. https://clinicaltrials.gov/study/NCT00366340 (2012)
89. Pfizer. A phase 3, randomised, active-controlled, double-blind trial of the safety, tolerability and immunologic noninferiority of a 13-valent pneumococcal conjugate vaccine compared to a 7-valent pneumococcal conjugate vaccine in healthy infants given in a 2-, 3-, 4- and 11- to 12-months schedule with routine pediatric vaccinations. https://www.clinicaltrialsregister.eu/ctr-search/trial/2005-004770-24/results (2016)
90. Esposito S, Tansey S, Thompson A, et al. Safety and immunogenicity of a 13-valent pneumococcal conjugate vaccine compared to those of a 7-valent pneumococcal conjugate vaccine given as a three-dose series with routine vaccines in healthy infants and toddlers. *Clin Vaccine Immunol* **17**,1017-26 (2010).
91. Wyeth. Study evaluating a 13-valent pneumococcal conjugate vaccine in infants. https://clinicaltrials.gov/study/NCT00366899 (2013)
92. Pfizer. A phase 3, randomized, active-controlled, double-blind trial evaluating the safety, tolerability, and immunogenicity of a 13-valent pneumococcal conjugate vaccine in healhy infants given with routine pediatric vaccination in italy. https://www.clinicaltrialsregister.eu/ctr-search/search?query=eudract_number:2005-004771-38 (2016)
93. Rodgers GL, Esposito S, Principi N, et al. Immune response to 13-valent pneumococcal conjugate vaccine with a reduced dosing schedule. *Vaccine* **31**,4765-74 (2013).
94. Snape MD, Klinger CL, Daniels ED, et al. Immunogenicity and reactogenicity of a 13-valent-pneumococcal conjugate vaccine administered at 2, 4, and 12 months of age: A double-blind randomized active-controlled trial. *Pediatr Infect Dis J* **29**,e80-90 (2010).
95. Wyeth. Study to evaluate a 13-valent pneumococcal conjugate vaccine in infants. https://clinicaltrials.gov/study/NCT00384059 (2013)
96. Pfizer. A phase 3, randomized, active-controlled, double-blind trial evaluating the safety, tolerability, and immunogenicity of a 13-valent pneumococcal conjugate vaccine in healthy infants given with routine pediatric vaccinations in the united kingdom. https://www.clinicaltrialsregister.eu/ctr-search/search?query=eudract_number:2005-005130-12 (2016)
97. Bryant KA, Block SL, Baker SA, Gruber WC, Scott DA. Safety and immunogenicity of a 13-valent pneumococcal conjugate vaccine. *Pediatrics* **125**,866-75 (2010).
98. Wyeth. Study evaluating pneumococcal vaccine in healthy infants. https://clinicaltrials.gov/study/NCT00205803 (2012)
99. Yeh SH, Gurtman A, Hurley DC, et al. Immunogenicity and safety of 13-valent pneumococcal conjugate vaccine in infants and toddlers. *Pediatrics* **126**,e493-505 (2010).
100. Pfizer. Study comparing 13-valent pneumococcal conjugate vaccine with 7-valent pneumococcal conjugate vaccine. https://clinicaltrials.gov/study/NCT00373958 (2013)
101. Weckx LY, Thompson A, Berezin EN, et al. A phase 3, randomized, double-blind trial comparing the safety and immunogenicity of the 7-valent and 13-valent pneumococcal conjugate vaccines, given with routine pediatric vaccinations, in healthy infants in brazil. *Vaccine* **30**,7566-72 (2012).
102. Wyeth. Study evaluating 13-valent pneumococcal conjugate vaccine in healthy infants in brazil. https://clinicaltrials.gov/study/NCT00676091 (2011)
103. Huang LM, Lin TY, Juergens C. Immunogenicity and safety of a 13-valent pneumococcal conjugate vaccine given with routine pediatric vaccines in taiwan. *Vaccine* **30**,2054-9 (2012).
104. Wyeth. Study evaluating a 13-valent pneumococcal conjugate vaccine administered to infants in taiwan. https://clinicaltrials.gov/study/NCT00688870 (2022)
105. Amdekar YK, Lalwani SK, Bavdekar A, et al. Immunogenicity and safety of a 13-valent pneumococcal conjugate vaccine in healthy infants and toddlers given with routine vaccines in india. *Pediatr Infect Dis J* **32**,509-16 (2013).
106. Dagan R, Patterson S, Juergens C, et al. Comparative immunogenicity and efficacy of 13-valent and 7-valent pneumococcal conjugate vaccines in reducing nasopharyngeal colonization: A randomized double-blind trial. *Clin Infect Dis* **57**,952-62 (2013).
107. Juergens C, Patterson S, Trammel J, et al. Post hoc analysis of a randomized double-blind trial of the correlation of functional and binding antibody responses elicited by 13-valent and 7-valent pneumococcal conjugate vaccines and association with nasopharyngeal colonization. *Clin Vaccine Immunol* **21**,1277-81 (2014).
108. Dagan R, Jiang Q, Juergens C, Trammel J, Gruber WC, Scott DA. Carrier-induced hyporesponsiveness to pneumococcal conjugate vaccines: Unraveling the influence of serotypes, timing, and previous vaccine dose. *Clin Infect Dis* **72**,448-54 (2021).

109. Kim DS, Shin SH, Lee HJ, et al. Immunogenicity and safety of 13-valent pneumococcal conjugate vaccine given to korean children receiving routine pediatric vaccines. *Pediatr Infect Dis J* **32**,266-73 (2013).
110. Wyeth. Study evaluating a 13-valent pneumococcal conjugate vaccine administered to infants in korea. https://clinicaltrials.gov/study/NCT00689351 (2011)
111. Diez-Domingo J, Gurtman A, Bernaola E, et al. Evaluation of 13-valent pneumococcal conjugate vaccine and concomitant meningococcal group c conjugate vaccine in healthy infants and toddlers in spain. *Vaccine* **31**,5486-94 (2013).
112. Wyeth. Study to evaluate a 13-valent pneumococcal conjugate vaccine in infants (nct00368966). https://clinicaltrials.gov/study/NCT00368966 (2012)
113. Payton T, Girgenti D, Frenck RW, et al. Immunogenicity, safety and tolerability of 3 lots of 13-valent pneumococcal conjugate vaccine given with routine pediatric vaccinations in the united states. *Pediatr Infect Dis J* **32**,871-80 (2013).
114. Wyeth. Study evaluating 13-valent pneumococcal conjugate vaccine in healthy infants. https://clinicaltrials.gov/study/NCT00444457 (2012)
115. Togashi T, Okada K, Yamaji M, et al. Immunogenicity and safety of a 13-valent pneumococcal conjugate vaccine given with dtap vaccine in healthy infants in japan. *Pediatr Infect Dis J* **34**,1096-104 (2015).
116. Pfizer. Trial evaluating a 13-valent pneumococcal conjugate vaccine given with diphtheria, tetanus, and acellular pertussis vaccine (dtap) in healthy japanese infants. https://clinicaltrials.gov/study/NCT01200368 (2018)
117. Zhu F, Hu Y, Li J, et al. Immunogenicity and safety of 13-valent pneumococcal conjugate vaccine compared with 7-valent pneumococcal conjugate vaccine among healthy infants in china. *Pediatr Infect Dis J* **35**,999-1010 (2016).
118. Zhu F, Hu Y, Li J, et al. Immunogenicity and safety of the 13-valent pneumococcal conjugate vaccine administered in a 3 + 1 versus 2 + 1 dose schedule among infants in china. *Pediatr Infect Dis J* **38**,1150-58 (2019).
119. Wyeth. Study evaluating 13-valent pneumococcal conjugate vaccine in healthy infants in india. https://clinicaltrials.gov/study/NCT00452790 (2011)
120. Gadzinowski J, Albrecht P, Hasiec B, et al. Phase 3 trial evaluating the immunogenicity, safety, and tolerability of manufacturing scale 13-valent pneumococcal conjugate vaccine. *Vaccine* **29**,2947-55 (2011).
121. Wyeth. Wyeth study to evaluate a 13-valent pneumococcal conjugate vaccine in infants. https://clinicaltrials.gov/study/NCT00464945 (2012)
122. Pfizer. A phase 3, randomized, active-controlled, double-blind trial evaluating the safety, tolerability, and immunogenicity of manufacturing scale 13-valent pneumococcal conjugate vaccine. https://www.clinicaltrialsregister.eu/ctr-search/trial/2006-006204-11/results (2016)
123. Vanderkooi OG, Scheifele DW, Girgenti D, et al. Safety and immunogenicity of a 13-valent pneumococcal conjugate vaccine in healthy infants and toddlers given with routine pediatric vaccinations in canada. *Pediatr Infect Dis J* **31**,72-7 (2012).
124. Wyeth. Study evaluating 13-valent pneumococcal conjugate vaccine in healthy infants. https://clinicaltrials.gov/study/NCT00475033 (2011)
125. Spijkerman J, Veenhoven RH, Wijmenga-Monsuur AJ, et al. Immunogenicity of 13-valent pneumococcal conjugate vaccine administered according to 4 different primary immunization schedules in infants: A randomized clinical trial. *Jama* **310**,930-7 (2013).
126. van Westen E, Knol MJ, Wijmenga-Monsuur AJ, et al. Serotype-specific igg antibody waning after pneumococcal conjugate primary series vaccinations with either the 10-valent or the 13-valent vaccine. *Vaccines (Basel)* **6** (2018).
127. Martinón-Torres F, Gimenez-Sanchez F, Gurtman A, et al. 13-valent pneumococcal conjugate vaccine given with meningococcal c-tetanus toxoid conjugate and other routine pediatric vaccinations: Immunogenicity and safety. *Pediatr Infect Dis J* **31**,392-9 (2012).
128. Wyeth. Study evaluating a 13-valent pneumococcal conjugate vaccine in healthy infants. https://clinicaltrials.gov/study/NCT00474539 (2013)
129. Pfizer. A phase 3, randomized, active-controlled, double-blind trial evaluating the safety, tolerability and immunogenicity of a 13-valent pneumococcal conjugate vaccine in healthy infants given with a meningococcal c-tetanus toxoid conjugate vaccine and other routine pediatric vaccinations in spain. https://www.clinicaltrialsregister.eu/ctr-search/search?query=2007-000304-32 (2016)
130. Gadzinowski J, Tansey SP, Wysocki J, et al. Safety and immunogenicity of a 13-valent pneumococcal conjugate vaccine manufactured with and without polysorbate 80 given to healthy infants at 2, 3, 4 and 12 months of age. *Pediatr Infect Dis J* **34**,180-5 (2015).
131. Wyeth. Study to evaluate a 13-valent pneumococcal conjugate vaccine in infants (nct00373958). https://clinicaltrials.gov/study/NCT00366548 (2012)

132. Iro MA, Khatami A, Marshall AS, et al. Immunological effect of administration of sequential doses of haemophilus influenzae type b and pneumococcal conjugate vaccines in the same versus alternating limbs in the routine infant immunisation schedule: An open-label randomised controlled trial. *Lancet Infect Dis* **15**,172-80 (2015).
133. Trück J, Jawad S, Goldblatt D, et al. The antibody response following a booster with either a 10- or 13-valent pneumococcal conjugate vaccine in toddlers primed with a 13-valent pneumococcal conjugate vaccine in early infancy. *Pediatr Infect Dis J* **35**,787-93 (2016).
134. Block SL, Shepard J, Garfield H, et al. Immunogenicity and safety of a 3- and 4-dose vaccination series of a meningococcal acwy conjugate vaccine in infants: Results of a phase 3b, randomized, open-label trial. *Pediatr Infect Dis J* **35**,e48-59 (2016).
135. Prymula R, Szenborn L, Silfverdal SA, et al. Safety, reactogenicity and immunogenicity of two investigational pneumococcal protein-based vaccines: Results from a randomized phase ii study in infants. *Vaccine* **35**,4603-11 (2017).
136. GlaxoSmithKline. Safety & immunogenicity of pneumococcal vaccine 2189242a co-administered with dtpa-hbv-ipv/hib in healthy infants. https://clinicaltrials.gov/study/NCT01204658 (2019)
137. Vesikari T, Rivera L, Korhonen T, et al. Immunogenicity and safety of primary and booster vaccination with 2 investigational formulations of diphtheria, tetanus and haemophilus influenzae type b antigens in a hexavalent dtpa-hbv-ipv/hib combination vaccine in comparison with the licensed infanrix hexa. *Hum Vaccin Immunother* **13**,1505-15 (2017).
138. GlaxoSmithKline. Safety and immunogenicity of new formulations of glaxosmithkline biologicals' dtpa-hbv-ipv/hib vaccine (gsk217744). https://clinicaltrials.gov/study/NCT01248884 (2018)
139. Idoko OT, Mboizi RB, Okoye M, et al. Immunogenicity and safety of 13-valent pneumococcal conjugate vaccine (pcv13) formulated with 2-phenoxyethanol in multidose vials given with routine vaccination in healthy infants: An open-label randomized controlled trial. *Vaccine* **35**,3256-63 (2017).
140. Pfizer. 13vpnc multidose vial safety, tolerability and immunogenicity study in healthy infants. https://clinicaltrials.gov/study/NCT01964716 (2015)
141. Pfizer. A phase 3, randomized, open-label trial to evaluate the safety, tolerability, and immunogenicity of 13-valent pneumococcal conjugate vaccine formulated in multidose vials given with routine pediatric vaccinations in healthy infants. https://www.clinicaltrialsregister.eu/ctr-search/search?query=eudract_number:2012-000482-21 (2015)
142. Wysocki J, Center KJ, Brzostek J, et al. A randomized study of fever prophylaxis and the immunogenicity of routine pediatric vaccinations. *Vaccine* **35**,1926-35 (2017).
143. Pfizer. Study assessing the effect of medications to prevent fever on prevenar 13. https://clinicaltrials.gov/study/NCT01392378 (2014)
144. Pfizer. A phase 4, randomized, open-label trial to assess the impact of prophylactic antipyretic medication on the immunogenicity of 13-valent pneumococcal conjugate vaccine given with routine pediatric vaccinations in healthy infants. https://www.clinicaltrialsregister.eu/ctr-search/search?query=eudract_number:2010-022303-22 (2016)
145. Cutland CL, Nolan T, Halperin SA, et al. Immunogenicity and safety of one or two doses of the quadrivalent meningococcal vaccine menacwy-tt given alone or with the 13-valent pneumococcal conjugate vaccine in toddlers: A phase iii, open-label, randomised study. *Vaccine* **36**,1908-16 (2018).
146. Pfizer. Immunogenicity and safety study of 1 and 2 doses of glaxosmithkline (gsk) biologicals' meningococcal vaccine menacwy-tt (gsk134612) in toddlers, persistence up to 5 years after vaccination and co-administration with pfizer's prevenar 13 vaccine. https://clinicaltrials.gov/study/NCT01939158 (2021)
147. Prymula R, Kieninger D, Feroldi E, Jordanov E, B'Chir S, DaCosta X. Immunogenicity and safety of primary and booster vaccinations of a fully liquid dtap-ipv-hb-prp-t hexavalent vaccine in healthy infants and toddlers in germany and the czech republic. *Pediatr Infect Dis J* **37**,823-30 (2018).
148. Pasteur S. Immunogenicity and safety study of a hexavalent dtap-ipv-hb-hib combined vaccine in a 3-dose primary series in healthy infants in europe. https://www.clinicaltrialsregister.eu/ctr-search/trial/2012-001055-39/results (2016)
149. SA SP. Booster effect and safety of a dtap-ipv-hib combined vaccine, with or without hep b, in healthy subjects 11 to 18 months of age who received a hexavalent or hexavalent/pentavalent combined vaccine during the primary series. https://www.clinicaltrialsregister.eu/ctr-search/search?query=eudract_number:2012-001042-18 (2017)
150. Goldblatt D, Southern J, Andrews NJ, et al. Pneumococcal conjugate vaccine 13 delivered as one primary and one booster dose (1 + 1) compared with two primary doses and a booster (2 + 1) in uk infants: A multicentre, parallel group randomised controlled trial. *Lancet Infect Dis* **18**,171-79 (2018).
151. University of Oxford CTaRGC. Assessment of post booster antibody responses in uk infants given a reduced priming schedule of meningococcal serogroup b and 13 valent pneumococcal conjugate vaccines. https://www.clinicaltrialsregister.eu/ctr-search/search?query=eudract_number:2015-000817-32 (2022)

152. Temple B, Toan NT, Dai VTT, et al. Immunogenicity and reactogenicity of ten-valent versus 13-valent pneumococcal conjugate vaccines among infants in ho chi minh city, vietnam: A randomised controlled trial. *Lancet Infect Dis* **19**,497-509 (2019).
153. Moïsi JC, Yaro S, Kroman SS, et al. Immunogenicity and reactogenicity of 13-valent pneumococcal conjugate vaccine among infants, toddlers, and children in western burkina faso: Results from a clinical trial of alternative immunization schedules. *J Pediatric Infect Dis Soc* **8**,422-32 (2019).
154. Carmona Martinez A, Prymula R, Miranda Valdivieso M, et al. Immunogenicity and safety of 11- and 12-valent pneumococcal non-typeable haemophilus influenzae protein d-conjugate vaccines (11vphid-cv, 12vphid-cv) in infants: Results from a phase ii, randomised, multicentre study. *Vaccine* **37**,176-86 (2019).
155. GlaxoSmithKline. Immunogenicity and safety of two formulations of gsk biologicals' pneumococcal vaccine (2830929a and 2830930a) when administered in healthy infants. https://clinicaltrials.gov/study/NCT01616459 (2019)
156. Odutola A, Ota MOC, Antonio M, et al. Immunogenicity of pneumococcal conjugate vaccine formulations containing pneumococcal proteins, and immunogenicity and reactogenicity of co-administered routine vaccines - a phase ii, randomised, observer-blind study in gambian infants. *Vaccine* **37**,2586-99 (2019).
157. GlaxoSmithKline. Impact of gsk biologicals' 2189242a vaccine on nasopharyngeal carriage, safety & immunogenicity in children & infants. https://clinicaltrials.gov/study/NCT01262872 (2019)
158. Klein NP, Abu-Elyazeed R, Baine Y, Cheuvart B, Silerova M, Mesaros N. Immunogenicity and safety of the haemophilus influenzae type b and neisseria meningitidis serogroups c and y-tetanus toxoid conjugate vaccine co-administered with human rotavirus, hepatitis a and 13-valent pneumococcal conjugate vaccines: Results from a phase iii, randomized, multicenter study in infants. *Hum Vaccin Immunother* **15**,327-38 (2019).
159. GlaxoSmithKline. Immunogenicity, safety and reactogenicity study of glaxosmithkline (gsk) biologicals' hib-mency-tt (menhibrix®) vaccine compared to merck & co, inc. Pedvaxhib vaccine in healthy infants and toddlers 12 to 15 months of age. https://clinicaltrials.gov/study/NCT01978093 (2018)
160. Madhi SA, Mutsaerts EA, Izu A, et al. Immunogenicity of a single-dose compared with a two-dose primary series followed by a booster dose of ten-valent or 13-valent pneumococcal conjugate vaccine in south african children: An open-label, randomised, non-inferiority trial. *Lancet Infect Dis* **20**,1426-36 (2020).
161. Mutsaerts E, van Cranenbroek B, Madhi SA, et al. Impact of nutritional status on vaccine-induced immunity in children living in south africa: Investigating the b-cell repertoire and metabolic hormones. *Vaccine* **42**,3337-45 (2024).
162. Shin J, Teeratakulpisarn J, Puthanakit T, et al. Immunogenicity and safety of a 12-valent pneumococcal conjugate vaccine in infants aged 6-10 weeks: A randomized double-blind active-controlled trial. *Clin Exp Pediatr* **63**,265-71 (2020).
163. Leach AJ, Mulholland EK, Santosham M, et al. Interchangeability, immunogenicity and safety of a combined 10-valent pneumococcal haemophilus influenzae protein d conjugate vaccine (synflorix) and 13-valent-pcv (prevenar13) schedule at 1-2-4-6 months: Previx_combo, a 3-arm randomised controlled trial. *Vaccine X* **7**,100086 (2021).
164. Leach AJ, Wilson N, Arrowsmith B, et al. Immunogenicity, otitis media, hearing impairment, and nasopharyngeal carriage 6-months after 13-valent or ten-valent booster pneumococcal conjugate vaccines, stratified by mixed priming schedules: Previx_combo and previx_boost randomised controlled trials. *Lancet Infect Dis* **22**,1374-87 (2022).
165. Lalwani SK, Ramanan PV, Sapru A, et al. Safety and immunogenicity of a multidose vial formulation of 13-valent pneumococcal conjugate vaccine administered with routine pediatric vaccines in healthy infants in india: A phase 4, randomized, open-label study. *Vaccine* **39**,6787-95 (2021).
166. Pfizer. Study to describe the safety, tolerability, and immunogenicity of 13- valent pneumococcal conjugate vaccine formulated in multidose vials when given with routine pediatric vaccines in healthy infants in india. https://clinicaltrials.gov/study/NCT03548337 (2020)
167. Dhingra MS, Namazova-Baranova L, Arredondo-Garcia JL, et al. Immunogenicity and safety of a quadrivalent meningococcal tetanus toxoid-conjugate vaccine administered concomitantly with other paediatric vaccines in toddlers: A phase iii randomised study. *Epidemiol Infect* **149**,e90 (2021).
168. Pasteur S. Immunogenicity and safety of a meningococcal conjugate vaccine given concomitantly with other vaccines in toddlers. https://clinicaltrials.gov/study/NCT03205371 (2022)
169. Wang W, Liang Q, Zhu J, et al. Immunogenicity and safety of a 13-valent pneumococcal conjugate vaccine administered in a prime-boost regimen among chinese infants: A randomized, double blind phase iii clinical trial. *Hum Vaccin Immunother* **18**,2019498 (2022).
170. Kawade A, Dayma G, Apte A, et al. Effect of reduced two-dose (1+1) schedule of 10 and 13-valent pneumococcal conjugate vaccines (synflorix(tm) and prevenar13(tm))) on nasopharyngeal carriage and serotype-specific immune response in the first two years of life: Results from an open-labelled randomized controlled trial in indian children. *Vaccine* **41**,3066-79 (2023).

171. Sanchez L, Rungmaitree S, Kosalaraksa P, et al. Immunogenicity and safety of a hexavalent dtwp-ipv-hb-prp~t vaccine versus separate dtwp-hb-prp~t, bopv, and ipv vaccines administered at 2, 4, 6 months of age concomitantly with rotavirus and pneumococcal conjugate vaccines in healthy infants in thailand. *Pediatr Infect Dis J* **42**,711-18 (2023).
172. Rajan M, Marchevsky N, Sinclair G, et al. A randomized trial assessing the immunogenicity and reactogenicity of two hexavalent infant vaccines concomitantly administered with group b meningococcal vaccine. *Pediatr Infect Dis J* **42**,66-73 (2023).
173. Simon MW, Bataille R, Caldwell NS, et al. Safety and immunogenicity of a multivalent pneumococcal conjugate vaccine given with 13-valent pneumococcal conjugate vaccine in healthy infants: A phase 2 randomized trial. *Hum Vaccin Immunother* **19**,2245727 (2023).
174. Pfizer. Study to evaluate the safety and immunogenicity of a multivalent pneumococcal vaccine given with prevnar 13 in healthy infants. https://clinicaltrials.gov/study/NCT03550313 (2021)
175. Temple B, Tran HP, Dai VTT, et al. Efficacy against pneumococcal carriage and the immunogenicity of reduced-dose (0 + 1 and 1 + 1) pcv10 and pcv13 schedules in ho chi minh city, viet nam: A parallel, single-blind, randomised controlled trial. *Lancet Infect Dis* **23**,933-44 (2023).
176. Xie Z, Li J, Wang X, et al. The safety and immunogenicity of a 13-valent pneumococcal polysaccharide conjugate vaccine (crm197/tt) in infants: A double-blind, randomized, phase iii trial. *Vaccines (Basel)* **12** (2024).
177. Matur RV, Thuluva S, Gunneri S, et al. Immunogenicity and safety of a 14-valent pneumococcal polysaccharide conjugate vaccine (pneubevax 14™) administered to 6-8 weeks old healthy indian infants: A single blind, randomized, active-controlled, phase-iii study. *Vaccine* **42**,3157-65 (2024).
178. Gallagher KE, Lucinde R, Bottomley C, et al. Fractional doses of pneumococcal conjugate vaccine - a noninferiority trial. *N Engl J Med* **391**,2003-13 (2024).
179. Borys D, Rupp R, Smulders R, et al. Safety, tolerability and immunogenicity of a novel 24-valent pneumococcal vaccine in toddlers: A phase 1 randomized controlled trial. *Vaccine* **42**,2560-71 (2024).
180. GlaxoSmithKline. Study of glaxosmithkline biologicals' gsk2202083a vaccine in healthy infants. https://clinicaltrials.gov/study/NCT01090453 (2018)
181. GlaxoSmithKline. This study will evaluate the immunogenicity, reactogenicity and safety of the routine infant vaccines pediarix®, hiberix® and prevenar 13® when co-administered with glaxosmithkline (gsk) biologicals' liquid human rotavirus vaccine (hrv) as compared to gsk's licensed lyophilized vaccine. https://clinicaltrials.gov/study/NCT03207750 (2020)
182. Bili A, Dobson S, Quinones J, et al. A phase 3, multicenter, randomized, double-blind study to evaluate the interchangeability of v114, a 15-valent pneumococcal conjugate vaccine, and pcv13 with respect to safety, tolerability, and immunogenicity in healthy infants (pneu-direction). *Vaccine* **41**,657-65 (2023).
183. Merck Sharp & Dohme LLC. A study to evaluate the interchangeability of v114 and prevnar 13 in healthy infants (v114-027/pneu-direction). https://clinicaltrials.gov/study/NCT03620162 (2023)
184. Merck Sharp & Dohme LLC. A phase 3, multicenter, randomized, double-blind study to evaluate the interchangeability of v114 and prevnar 13™ with respect to safety, tolerability, and immunogenicity in healthy infants (pneu-direction). https://www.clinicaltrialsregister.eu/ctr-search/search?query=eudract_number:2018-001151-12 (2021)
185. Pfizer. Safety and immunogenicity of 20vpnc in toddlers with 2 prior doses of prevenar 13. https://clinicaltrials.gov/study/NCT05408429 (2024)
186. Pfizer. A phase 3, randomized, partially double-blind trial to evaluate the safety and immunogenicity of 20-valent pneumococcal conjugate vaccine (20-vpnc) in healthy toddlers 12 through 23 months of age with 2 prior infant doses of prevenar 13. https://www.clinicaltrialsregister.eu/ctr-search/search?query=eudract_number:2021-006624-41 (2023)
187. Greenberg D, Hoover PA, Vesikari T, et al. Safety and immunogenicity of 15-valent pneumococcal conjugate vaccine (pcv15) in healthy infants. *Vaccine* **36**,6883-91 (2018).
188. Merck Sharp & Dohme LLC. A study of pneumococcal conjugate vaccine (v114) compared to a marketed vaccine (v114-003). https://clinicaltrials.gov/study/NCT01215188 (2019)
189. Merck Sharp & Dohme LLC. Immunogenicity and safety study of a hexavalent dtap-ipv-hb-hib combined vaccine in a 3-dose primary series in healthy infants in europe. https://www.clinicaltrialsregister.eu/ctr-search/search?query=eudract_number:2010-019775-29 (2019)
190. Rupp R, Hurley D, Grayson S, et al. A dose ranging study of 2 different formulations of 15-valent pneumococcal conjugate vaccine (pcv15) in healthy infants. *Hum Vaccin Immunother* **15**,549-59 (2019).
191. Merck Sharp & Dohme LLC. A study to evaluate the safety, tolerability and immunogenicity of v114 in healthy adults and infants (v114-005). https://clinicaltrials.gov/study/NCT02531373 (2019)
192. Platt HL, Greenberg D, Tapiero B, et al. A phase ii trial of safety, tolerability and immunogenicity of v114, a 15-valent pneumococcal conjugate vaccine, compared with 13-valent pneumococcal conjugate vaccine in healthy infants. *Pediatr Infect Dis J* **39**,763-70 (2020).

193. Merck Sharp & Dohme LLC. A study to evaluate the safety, tolerability, and immunogenicity of two lots of v114 in healthy infants (v114-008). https://clinicaltrials.gov/study/NCT02987972 (2019)
194. Banniettis N, Wysocki J, Szenborn L, et al. A phase iii, multicenter, randomized, double-blind, active comparator-controlled study to evaluate the safety, tolerability, and immunogenicity of catch-up vaccination regimens of v114, a 15-valent pneumococcal conjugate vaccine, in healthy infants, children, and adolescents (pneu-plan). *Vaccine* **40**,6315-25 (2022).
195. Merck Sharp & Dohme LLC. A phase 3, multicenter, randomized, double-blind, active comparator-controlled study to evaluate the safety, tolerability, and immunogenicity of catch-up vaccination regimens of v114 in healthy infants, children, and adolescents (pneu-plan). https://www.clinicaltrialsregister.eu/ctr-search/trial/2018-003706-88/results (2021)
196. Merck Sharp & Dohme LLC. Safety and immunogenicity of catch-up vaccination regimens of v114 (v114-024) (pneu-plan). https://clinicaltrials.gov/study/NCT03885934 (2023)
197. Martinon-Torres F, Wysocki J, Szenborn L, et al. A phase iii, multicenter, randomized, double-blind, active comparator-controlled study to evaluate the safety, tolerability, and immunogenicity of v114 compared with pcv13 in healthy infants (pneu-ped-eu-1). *Vaccine* **41**,3387-98 (2023).
198. Merck Sharp & Dohme LLC. Safety, tolerability, and immunogenicity of v114 in healthy infants (v114-025) (pneu-ped-eu-1). https://clinicaltrials.gov/study/NCT04031846 (2023)
199. Benfield T, Rämet M, Valentini P, et al. Safety, tolerability, and immunogenicity of v114 pneumococcal vaccine compared with pcv13 in a 2+1 regimen in healthy infants: A phase iii study (pneu-ped-eu-2). *Vaccine* **41**,2456-65 (2023).
200. Merck Sharp & Dohme LLC. Safety, tolerability, and immunogenicity of a 3-dose regimen of v114 in healthy infants (pneu-ped-eu-2/v114-026) (pneu-ped-eu-2). https://clinicaltrials.gov/study/NCT04016714?tab=results#outcome-measures (2023)
201. Suzuki H, Fujita H, Iwai K, et al. Safety and immunogenicity of 15-valent pneumococcal conjugate vaccine in japanese healthy infants: A phase iii study (v114-033). *Vaccine* **41**,4933-40 (2023).
202. Merck Sharp & Dohme LLC. Study to evaluate the safety, tolerability, and immunogenicity of v114 in healthy japanese infants (v114-033). https://clinicaltrials.gov/study/NCT04384107 (2023)
203. Ishihara Y, Kuroki H, Hidaka H, et al. Safety and immunogenicity of a 15-valent pneumococcal conjugate vaccine in japanese healthy infants: A phase i study (v114-028). *Hum Vaccin Immunother* **19**,2180973 (2023).
204. Wan K, Shirakawa M, Sawata M. Descriptive analysis of safety and immunogenicity profiles of a 15-valent pneumococcal conjugate vaccine between subcutaneous and intramuscular administration in a phase 1 study of healthy japanese infants (v114-028). *J Infect Chemother* **31**,102539 (2025).
205. Merck Sharp & Dohme LLC. Safety, tolerability, and immunogenicity of v114 in healthy japanese infants (v114-028). https://clinicaltrials.gov/study/NCT03848065 (2025)
206. Lupinacci R, Rupp R, Wittawatmongkol O, et al. A phase 3, multicenter, randomized, double-blind, active-comparator-controlled study to evaluate the safety, tolerability, and immunogenicity of a 4-dose regimen of v114, a 15-valent pneumococcal conjugate vaccine, in healthy infants (pneu-ped). *Vaccine* **41**,1142-52 (2023).
207. Merck Sharp & Dohme LLC. Safety, tolerability, and immunogenicity of v114 in healthy infants (v114-029) (pneu-ped). https://clinicaltrials.gov/study/NCT03893448 (2023)
208. Senders S, Klein NP, Lamberth E, et al. Safety and immunogenicity of a 20-valent pneumococcal conjugate vaccine in healthy infants in the united states. *The Pediatric Infectious Disease Journal* **40** (2021).
209. Pfizer. Trial to evaluate the safety and immunogenicity of a multivalent pneumococcal vaccine in healthy infants. https://clinicaltrials.gov/study/NCT03512288 (2021)
210. Korbal P, Wysocki J, Jackowska T, et al. Phase 3 safety and immunogenicity study of a three-dose series of twenty-valent pneumococcal conjugate vaccine in healthy infants and toddlers. *Pediatr Infect Dis J* **43**,587-95 (2024).
211. Pfizer. 20-valent pneumococcal conjugate vaccine safety and immunogenicity study of a 3-dose series in healthy infants. https://clinicaltrials.gov/study/NCT04546425 (2020)
212. Ishihara Y, Fukazawa M, Enomoto S, et al. A phase 3 randomized study to evaluate safety and immunogenicity of 20-valent pneumococcal conjugate vaccine in healthy japanese infants. *Int J Infect Dis* **141**,106942 (2024).
213. Pfizer. 20-valent pneumococcal conjugate vaccine safety and immunogenicity study in healthy japanese infants. https://clinicaltrials.gov/study/NCT04530838 (2023)
214. Senders S, Klein NP, Tamimi N, et al. A phase three study of the safety and immunogenicity of a four-dose series of 20-valent pneumococcal conjugate vaccine in healthy infants. *Pediatr Infect Dis J* **43**,596-603 (2024).
215. Pfizer. 20-valent pneumococcal conjugate vaccine safety and immunogenicity study of a 4-dose series in healthy infants. https://clinicaltrials.gov/study/NCT04382326 (2023)

216. Pfizer. A phase 3, randomized, double-blind trial to evaluate the safety and immunogenicity of a 20-valent pneumococcal conjugate vaccine in healthy infants. https://www.clinicaltrialsregister.eu/ctr-search/search?query=eudract_number:2019-003305-10 (2023)
217. Clarke E, Bashorun AO, Okoye M, et al. Safety and immunogenicity of a novel 10-valent pneumococcal conjugate vaccine candidate in adults, toddlers, and infants in the gambia-results of a phase 1/2 randomized, double-blinded, controlled trial. *Vaccine* **38**,399-410 (2020).
218. PATH. Safety and immunogenicity of a 10 valent pneumococcal conjugate vaccine (siilpcv10) in healthy adults, toddlers, infants. https://clinicaltrials.gov/study/NCT02308540 (2019)
219. Clarke E, Bashorun A, Adigweme I, et al. Immunogenicity and safety of a novel ten-valent pneumococcal conjugate vaccine in healthy infants in the gambia: A phase 3, randomised, double-blind, non-inferiority trial. *Lancet Infect Dis* **21**,834-46 (2021).
220. PATH. Phase 3 study of 10-valent pneumococcal conjugate vaccine (pneumosil) in healthy infants. https://clinicaltrials.gov/study/NCT03197376 (2020)
221. Adigweme I, Futa A, Saidy-Jah E, et al. Immunogenicity and safety of a 10-valent pneumococcal conjugate vaccine administered as a 2 + 1 schedule to healthy infants in the gambia: A single-centre, double-blind, active-controlled, randomised, phase 3 trial. *Lancet Infect Dis* **23**,609-20 (2023).
222. PATH. Study of 10-valent pneumococcal conjugate vaccine (pneumosil) administered in a 2+1 schedule to healthy infants. https://clinicaltrials.gov/study/NCT03896477 (2022)
223. Nurkka A, Ahman H, Korkeila M, Jäntti V, Käyhty H, Eskola J. Serum and salivary anti-capsular antibodies in infants and children immunized with the heptavalent pneumococcal conjugate vaccine. *Pediatr Infect Dis J* **20**,25-33 (2001).
224. Käyhty H, Ahman H, Eriksson K, Sörberg M, Nilsson L. Immunogenicity and tolerability of a heptavalent pneumococcal conjugate vaccine administered at 3, 5 and 12 months of age. *Pediatr Infect Dis J* **24**,108-14 (2005).
225. Shao PL, Lu CY, Chang LY, et al. Safety and immunogenicity of heptavalent pneumococcal conjugate vaccine booster in taiwanese toddlers. *J Formos Med Assoc* **105**,542-9 (2006).
226. Kim NH, Lee J, Lee SJ, et al. Immunogenicity and safety of pneumococcal 7-valent conjugate vaccine (diphtheria crm(197) protein conjugate; prevenar ) in korean infants: Differences that are found in asian children. *Vaccine* **25**,7858-65 (2007).
227. Lee H, Nahm MH, Burton R, Kim KH. Immune response in infants to the heptavalent pneumococcal conjugate vaccine against vaccine-related serotypes 6a and 19a. *Clin Vaccine Immunol* **16**,376-81 (2009).
228. Li R, Huang L, Mo S, et al. Safety, tolerability, and immunogenicity of 7-valent pneumococcal conjugate vaccine in older infants and young children in china who are naive to pneumococcal vaccination: Results of a phase 4 open-label trial. *Vaccine* **33**,3580-5 (2015).
229. Togashi T, Yamaji M, Thompson A, et al. Immunogenicity and safety of a 13-valent pneumococcal conjugate vaccine in healthy infants in japan. *Pediatr Infect Dis J* **32**,984-9 (2013).
230. Wyeth. Study evaluating 13-valent pneumococcal conjugate vaccine in healthy japanese infants. https://clinicaltrials.gov/study/NCT00574795 (2012)
231. Gutiérrez Brito M, Thompson A, Girgenti D, et al. Immunogenicity and safety of 13-valent pneumococcal conjugate vaccine in mexico. *Rev Panam Salud Publica* **33**,414-21 (2013).
232. Wyeth. Study evaluating 13-valent pneumococcal conjugate vaccine in healthy infants in mexico. https://clinicaltrials.gov/study/NCT00708682 (2011)
233. Singleton R, Wenger J, Klejka JA, et al. The 13-valent pneumococcal conjugate vaccine for invasive pneumococcal disease in alaska native children: Results of a clinical trial. *Pediatr Infect Dis J* **32**,257-63 (2013).
234. Wijmenga-Monsuur AJ, van Westen E, Knol MJ, et al. Direct comparison of immunogenicity induced by 10- or 13-valent pneumococcal conjugate vaccine around the 11-month booster in dutch infants. *PLoS One* **10**,e0144739 (2015).
235. van Westen E, Wijmenga-Monsuur AJ, van Dijken HH, et al. Differential b-cell memory around the 11-month booster in children vaccinated with a 10- or 13-valent pneumococcal conjugate vaccine. *Clin Infect Dis* **61**,342-9 (2015).
236. Martinón-Torres F, Czajka H, Center KJ, et al. 13-valent pneumococcal conjugate vaccine (pcv13) in preterm versus term infants. *Pediatrics* **135**,e876-86 (2015).
237. Martinón-Torres F, Wysocki J, Center KJ, et al. Circulating antibody 1 and 2 years after vaccination with the 13-valent pneumococcal conjugate vaccine in preterm compared with term infants. *Pediatr Infect Dis J* **36**,326-32 (2017).
238. Pfizer. Study evaluating a 13-valent pneumococcal conjugate vaccine in preterm compared to term infants. https://clinicaltrials.gov/study/NCT01193335 (2017)
239. Chu K, Hu Y, Pan H, et al. A randomized, open-label, phase 3 study evaluating safety and immunogenicity of 13-valent pneumococcal conjugate vaccine in chinese infants and children under 6 years of age. *Hum Vaccin Immunother* **19**,2235926 (2023).

240. Pfizer. A study assessing 13-valent pneumococcal conjugate vaccine in healthy chinese infants and young children. https://clinicaltrials.gov/study/NCT03574389 (2024)
241. Urbancikova I, Prymula R, Goldblatt D, Roalfe L, Prymulova K, Kosina P. Immunogenicity and safety of a booster dose of the 13-valent pneumococcal conjugate vaccine in children primed with the 10-valent or 13-valent pneumococcal conjugate vaccine in the czech republic and slovakia. *Vaccine* **35**,5186-93 (2017).
242. Maestri A, Park SE, Fernandes F, et al. A phase 3, single-arm, open-label study to evaluate the safety, tolerability, and immunogenicity of a 15-valent pneumococcal conjugate vaccine, v114, in a 3+1 regimen in healthy infants in south korea (pneu-ped-kor). *Hum Vaccin Immunother* **20**,2321035 (2024).
243. Merck Sharp & Dohme LLC. Safety and immunogenicity of v114 in healthy infants in south korea (v114-036) (pneu-ped-kor). https://clinicaltrials.gov/study/NCT04633226 (2024)
244. O'Brien KL, Swift AJ, Winkelstein JA, et al. Safety and immunogenicity of heptavalent pneumococcal vaccine conjugated to crm(197) among infants with sickle cell disease. Pneumococcal conjugate vaccine study group. *Pediatrics* **106**,965-72 (2000).
245. Esposito S, Pugni L, Bosis S, et al. Immunogenicity, safety and tolerability of heptavalent pneumococcal conjugate vaccine administered at 3, 5 and 11 months post-natally to pre- and full-term infants. *Vaccine* **23**,1703-8 (2005).
246. Osendarp SJ, Prabhakar H, Fuchs GJ, et al. Immunization with the heptavalent pneumococcal conjugate vaccine in bangladeshi infants and effects of zinc supplementation. *Vaccine* **25**,3347-54 (2007).
247. Vesikari T, Karvonen A, Prymula R, et al. Immunogenicity and safety of the human rotavirus vaccine rotarix co-administered with routine infant vaccines following the vaccination schedules in europe. *Vaccine* **28**,5272-9 (2010).
248. Moss SJ, Fenton AC, Toomey J, et al. Immunogenicity of a heptavalent conjugate pneumococcal vaccine administered concurrently with a combination diphtheria, tetanus, five-component acellular pertussis, inactivated polio, and haemophilus influenzae type b vaccine and a meningococcal group c conjugate vaccine at 2, 3, and 4 months of age. *Clin Vaccine Immunol* **17**,311-6 (2010).
249. Moss SJ, Fenton AC, Toomey JA, Grainger AJ, Smith J, Gennery AR. Responses to a conjugate pneumococcal vaccine in preterm infants immunized at 2, 3, and 4 months of age. *Clin Vaccine Immunol* **17**,1810-6 (2010).
250. Whelan J, Hahné S, Berbers G, van der Klis F, Wijnands Y, Boot H. Immunogenicity of a hexavalent vaccine co-administered with 7-valent pneumococcal conjugate vaccine. Findings from the national immunisation programme in the netherlands. *Hum Vaccin Immunother* **8**,743-8 (2012).
251. Jones SA, Groome M, Koen A, et al. Immunogenicity of seven-valent pneumococcal conjugate vaccine administered at 6, 14 and 40 weeks of age in south african infants. *PLoS One* **8**,e72794 (2013).
252. Madhi SA, Izu A, Violari A, et al. Immunogenicity following the first and second doses of 7-valent pneumococcal conjugate vaccine in hiv-infected and -uninfected infants. *Vaccine* **31**,777-83 (2013).
253. Madhi SA, Adrian P, Cotton MF, et al. Effect of hiv infection status and anti-retroviral treatment on quantitative and qualitative antibody responses to pneumococcal conjugate vaccine in infants. *J Infect Dis* **202**,355-61 (2010).
254. Madhi SA, Izu A, Violari A, et al. Effect of hiv-exposure and timing of antiretroviral treatment initiation in children living with hiv on antibody persistence and memory responses to haemophilus influenzae type b and pneumococcal polysaccharide-protein conjugate vaccines. *Vaccine* **38**,2651-59 (2020).
255. Ladhani SN, Andrews NJ, Waight P, et al. Interchangeability of meningococcal group c conjugate vaccines with different carrier proteins in the united kingdom infant immunisation schedule. *Vaccine* **33**,648-55 (2015).
256. Ladhani SN, Andrews NJ, Southern J, et al. Antibody responses after primary immunization in infants born to women receiving a pertussis-containing vaccine during pregnancy: Single arm observational study with a historical comparator. *Clin Infect Dis* **61**,1637-44 (2015).
257. Maertens K, Burbidge P, Van Damme P, Goldblatt D, Leuridan E. Pneumococcal immune response in infants whose mothers received tetanus, diphtheria and acellular pertussis vaccination during pregnancy. *Pediatr Infect Dis J* **36**,1186-92 (2017).
258. Madhi SA, Koen A, Cutland CL, et al. Antibody kinetics and response to routine vaccinations in infants born to women who received an investigational trivalent group b streptococcus polysaccharide crm197-conjugate vaccine during pregnancy. *Clin Infect Dis* **65**,1897-904 (2017).
259. Zimmermann P, Perrett KP, Ritz N, et al. Biological sex influences antibody responses to routine vaccinations in the first year of life. *Acta Paediatr* **109**,147-57 (2020).
260. Zimmermann P, Perrett KP, Berbers G, Curtis N. Persistence of pneumococcal antibodies after primary immunisation with a polysaccharide-protein conjugate vaccine. *Arch Dis Child* **104**,680-84 (2019).
261. Zimmermann P, Perrett KP, Messina NL, et al. The effect of maternal immunisation during pregnancy on infant vaccine responses. *EClinicalMedicine* **13**,21-30 (2019).

262. Perrett KP, Halperin SA, Nolan T, et al. Impact of tetanus-diphtheria-acellular pertussis immunization during pregnancy on subsequent infant immunization seroresponses: Follow-up from a large randomized placebo-controlled trial. *Vaccine* **38**,2105-14 (2020).
263. Martinón-Torres F, Halperin SA, Nolan T, et al. Impact of maternal diphtheria-tetanus-acellular pertussis vaccination on pertussis booster immune responses in toddlers: Follow-up of a randomized trial. *Vaccine* **39**,1598-608 (2021).
264. GlaxoSmithKline. Immunogenicity and safety study of infanrix hexa in healthy infants born to mothers vaccinated with boostrix™ during pregnancy or immediately post-delivery. https://clinicaltrials.gov/study/NCT02422264 (2019)
265. GlaxoSmithKline. Evaluation of immunogenicity and safety of a booster dose of infanrix hexa™ in healthy infants born to mothers vaccinated with boostrix™ during pregnancy or immediately post-delivery. https://clinicaltrials.gov/study/NCT02853929 (2020)
